# Effect of Processing, Cultivar, and Crop Year on Volatile Composition in Pulses and Pulse Flours Analyzed by Headspace Solid‐Phase Microextraction Gas Chromatography–Mass Spectrometry

**DOI:** 10.1111/1750-3841.70608

**Published:** 2025-10-15

**Authors:** Kaveri Ponskhe, Aubrey DuBois, Randolph Beaudry, Sharon Hooper, Karen Cichy, Emily J. Mayhew

**Affiliations:** ^1^ Department of Food Science and Human Nutrition Michigan State University East Lansing Michigan USA; ^2^ Department of Horticulture Michigan State University East Lansing Michigan USA; ^3^ Department of Plant, Soil and Microbial Sciences Michigan State University East Lansing Michigan USA; ^4^ Sugarbeet and Bean Research Unit USDA‐ARS East Lansing Michigan USA

## Abstract

**ABSTRACT:**

Low pulse consumption in the United States is linked to barriers such as lengthy cooking times, limited preparation knowledge, and undesirable taste or texture. Incorporating pulse flours into convenience products is a promising approach; however, volatile organic compounds responsible for off‐flavors hinder their broader acceptance. This study aimed to investigate and quantify the variation in the volatile composition of eight pulse cultivars due to the effects of cultivar, harvest year, and processing (roasting and boiling) using targeted headspace solid‐phase microextraction gas chromatography–mass spectrometry. Eight pulse varieties (Navy, Otebo, White Kidney, Great Northern, Cranberry, Mayacoba, Manteca, Chickpea) were produced by boiling whole or milling into flour, with a subset roasted before milling. These flours were also cooked into model products (porridge: roasted and nonroasted) to assess volatile changes due to roasting and subsequent cooking. Results showed significant differences in total estimated volatile concentration across processing treatments and harvest years. Boiling resulted in the lowest total volatile concentration (10.9 nmol/L), whereas nonroasted product exhibited the highest concentration (351 nmol/L), followed by roasted product (106 nmol/L), milled roasted flour (103 nmol/L), and milled nonroasted flour (53.3 nmol/L). Hierarchical clustering and principal component analysis revealed that samples clustered by harvest year with distinct volatile profiles across cultivars, suggesting that environmental conditions may influence volatile composition. These findings highlight the influence of cultivar selection, harvest year, and trade‐offs due to processing on pulse volatile profiles, providing insights that can mitigate off‐flavor formation and support the development of more widely accepted pulse‐based products.

**Practical Applications:**

The impact of harvest year, cultivar, and processing on volatile flavor chemistry of pulse flours is not well understood. This study shows how thermal treatments with wet or dry heat alter volatile composition across pulse genotypes. These insights can help the food industry to improve flavor of pulse flour and increase consumer consumption of underutilized, nutrient dense, sustainable pulses.

## 1 Introduction

Pulses are dry, edible seeds from leguminous crops such as dry beans, peas, chickpeas, and lentils. Their incorporation into food products is rising due to growing interest in plant‐based diets, driven by health and sustainability concerns (Chigwedere et al. 2022). Pulses are rich in protein (17%–30%), complex carbohydrates (60%–67%), and dietary fiber, while also providing essential vitamins and minerals (Boye et al. 2010; Campos‐Vega et al. 2010; Tosh and Yada 2010; Vaz Patto et al. 2015; Wang and Daun 2004). They also contribute to sustainable food systems by improving soil health through nitrogen fixation and promoting biodiversity through their genetic adaptability to adverse environments (Nulik et al. 2013; Russel 2015).

Milling pulses into flour enables their use in diverse cereal‐based products, improving nutritional quality and promoting environmental sustainability (Sadohara et al. 2022). However, off‐flavors limit their wider acceptance. These less acceptable off‐flavors have been described as “beany,” “grassy,” or “bitter” in products containing pinto bean, lupin, and cowpea flour (Nawaz et al. 2022; Simons and Hall 2018). As the use of pulse‐based ingredients grows, improving their sensory quality remains essential to consumer acceptance.

Volatile organic compounds (VOCs), especially those with low odor thresholds, contribute to pulse flavor. These volatiles mainly arise from three pathways: (1) oxidation of free fatty acids like linoleic and linolenic acids via lipoxygenase (LOX; Clemente et al. 2000; Karolkowski et al. 2021), (2) degradation of free amino acids via enzymatic or Maillard reactions (Bader et al. 2009; Jakobsen et al. 1998; Rizzi 1990; Spinnler 2011), and (3) carotenoid breakdown into terpenes (Maccarrone et al. 1994). The resulting compounds—aldehydes, alcohols, ketones, aromatics, terpenes, pyrazines, and sulfurs—are key drivers of pulse aroma. The perception of green, grassy, and beany notes is mainly attributed to aldehydes, alcohols, and ketones, while bitterness and astringency are linked to glycosylated compounds like saponins and both glycosylated and aglycone forms of phenolics, including flavonols and phenolic acids (Damodaran and Arora 2013; MacLeod et al. 1988; Roland et al. 2017; Ong and Liu 2018). These volatile profiles vary based on cultivar, year, and processing (Azarnia et al. 2011; Ma et al. 2016). For example, Azarnia et al. (2011) observed year‐to‐year differences in volatiles of pea cultivars, and Rajhi et al. (2021) reported volatile chemical class variation among pulses, such as high concentrations of aldehydes, alcohols, ketones, phenols, and hydrocarbons in faba beans, lentils, and chickpeas, while elevated oxygenated monoterpenes were observed in black and red beans. Characterizing volatiles across varying environmental conditions and bean cultivars is essential for improving flavor and identifying varieties best suited for targeted food applications.

Processing significantly influences pulse flavor by altering volatile composition. Techniques such as soaking, blanching, and dry heating can reduce off‐flavor compounds by inactivating LOX and other enzymes (Roland et al. 2017). Additionally, treatments like roasting may develop new compounds such as pyrazines that can help mask beany flavors (Ma et al. 2016). Although processing plays a key role in shaping flavor development of pulse flour, the changes in volatile compounds that occur during the final cooking step remain insufficiently explored, particularly in dry beans.

Headspace solid‐phase microextraction gas chromatography–mass spectrometry (HS–SPME–GC–MS) is a widely used method for studying VOCs in pulses, such as faba beans (Akkad et al. 2021, 2019; Oomah et al. 2014), peas (Azarnia et al. 2011), chickpeas (Zhao et al. 2021), and dry beans (Oomah et al. 2007), offering precise identification and quantification of volatiles.

Therefore, the specific objectives of this study were to evaluate the effect of: (1) processing (roasting and boiling), (2) cultivar, and (3) crop year on volatile compounds in eight selected pulse cultivars using HS–SPME–GC–MS.

## 2 Materials and Methods

### 2.1 Germplasm Selection and Seed Production

To assess the impact of cultivar and crop year on volatile profiles, seven bean cultivars grown during two different years (2022 and 2023) and one chickpea cultivar obtained from the market were studied. The Kabuli chickpea (Sierra) cultivar grown in 2022, obtained commercially, was chosen because of its importance in commercial production in the western United States, while the other seven bean varieties chosen for their adaptation to Michigan dry bean agricultural conditions, and favorable agronomic characteristics and competitive seed yields. The eight pulse varieties chosen for this study are listed in Table 1.

**TABLE 1:** Market class, abbreviations, and genotypes of the eight pulse varieties included in this study, with genotypes grown during the 2022 and 2023 crop years.

**Market class**	**Abbreviation**	**Genotypes grown in 2022**	**Genotypes grown in 2023**
Chickpea	CHKP	Sierra	—
Cranberry bean	CR	CR 1801‐2‐2	CR 2111‐1
Great Northern bean	GN	Powderhorn	Powderhorn
Manteca bean	MN	Y 1608‐7	Y 1608–14
Mayacoba bean	MY	Y 1802‐9‐1	Y 1802‐11‐2
Navy bean	N	Alpena	Alpena
Otebo bean	O	Samurai	Samurai
White Kidney bean	WK	WK 1601‐1	WK 1601‐1

The seven dry bean varieties were grown at the Michigan State University Montcalm Research Center in Entrican, Michigan in 2022 and 2023. The seeds were planted in a randomized complete block design with three field replications on June 10, 2022, and June 14, 2023, respectively. The plot consisted of four rows that were each 6.1 m long, with the center two rows containing the experimental lines and the outer two rows a standard kidney bean border. Recommended field maintenance practice was followed for weed and insect control and fertilization. Supplemental overhead irrigation was provided when needed. On September 29, 2022, and on October 11, 2023, respectively, the seeds were directly harvested with a Hege 140 plot combine harvester. Seed samples were cleaned by hand to remove gravel and damaged or foreign seeds, and cleaned samples were stored in paper bags at room temperature (22°C) until further processing. The light seed coat color of specific market classes such as white‐colored beans—Navy, Otebo, Great Northern, and White kidney were selected due to their potential for easier adoption as flour.

#### 2.1.1 Sample Preparation

**FIGURE 1:**
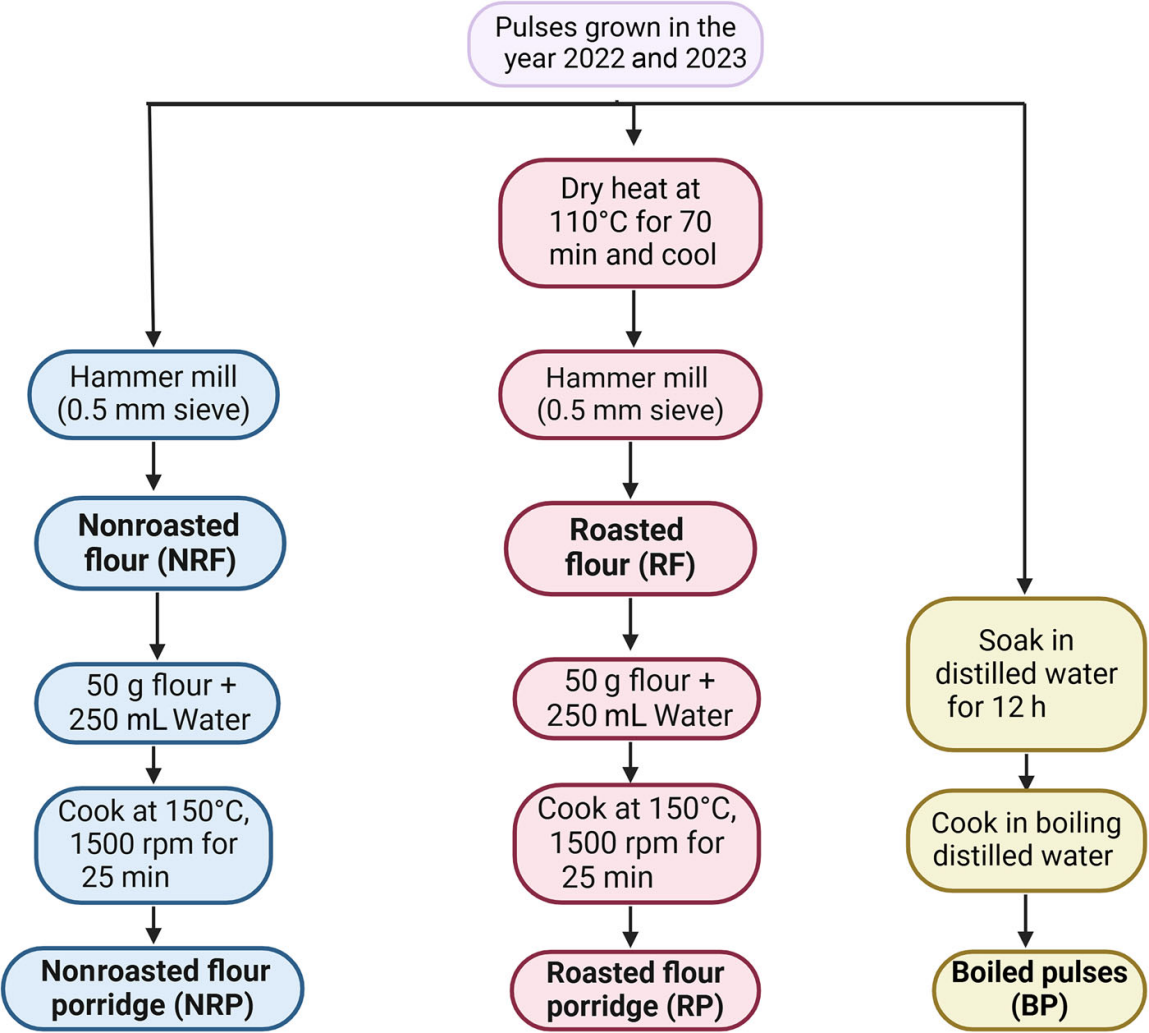
Flowchart of sample preparation methods for five types of samples, namely, BP, NRF, NRP, RF and RP. Each sample type was prepared from each of the seven beans, namely, Cranberry, Great Northern, Manteca, Mayacoba, Navy, Otebo, and White Kidney grown in the years 2022 and 2023, as well as Chickpea (market sample grown in 2022 acquired commercially), for GC–MS analysis.

Five types of samples were prepared for HS–SPME–GC–MS analysis from each of the eight pulses, namely, nonroasted pulse flour (NRF), roasted pulse flour (RF), nonroasted pulse flour porridge (NRP), roasted pulse flour porridge (RP), and boiled pulses (BPs) (Figure 1). To understand the volatile profiles of pulse varieties in their simplest minimally processed matrices, three processing treatments were selected. Boiling was used as a wet heat treatment for whole pulses, roasting served as a dry heat pretreatment prior to milling, and porridge preparation modeled a basic method for impact of cooking pulse flours with water. This design allowed for comparisons across thermal treatments (wet vs. dry heat) and processing formats (whole vs. flour) to assess the effects of processing on volatile compound profiles of eight pulse varieties across two harvest years. All sample quantities were determined through preliminary piloting and method development to ensure optimal peak resolution across sample types for GC–MS analysis.

The dry pulses were cleaned by rinsing under distilled water and then laid out on a sheet tray lined with a paper towel. A portion of the sample for each pulse type was subjected to dry heat roasting in an oven (Isotemp Gravity Oven, 100 L; Fisher Scientific, Waltham, MA, USA) at 110°C for 70 min, followed by a 4‐h cooling period. Both the nonroasted and roasted pulses were milled into flour using a hammer mill (Polymix Laboratory Grinding Mills, PX‐MFC 90 D, Kinematica; Bohemia, NY, USA) with a 0.5 mm sieve to obtain NRF and RF, respectively.

The NRF and RF samples were stored in resealable polyethylene plastic bags under refrigeration at 2°C to minimize volatile loss (Akkad et al. 2022). For pulses harvested in September 2022, milling into flour occurred in March 2023 (6 months postharvest), and GC–MS analysis was conducted in April 2024 (18 months postharvest). Similarly, for pulses harvested in October 2023, milling into flour occurred in April 2024 (6 months postharvest), with GC–MS analysis performed in September 2024 (12 months postharvest). The NRF and RF samples were transferred from refrigeration to room temperature (22°C) 30 min prior to porridge preparation and analysis by HS–SPME–GC–MS.

To investigate the effects of cooking on volatile compounds of pulse flours, model products in the form of porridges were prepared from both NRF and RF samples using a standardized procedure. Porridges were selected as the model system due to their simplicity, as water is the only added ingredient. For porridge preparation, 50 g of NRF or RF flour was mixed with 250 mL and stirred for 7 min on a magnetic hotplate stirrer (PRO 4‐Channel LCD Digital Magnetic Stirrer; MSE Supplies LLC, Tucson, AZ, USA) to ensure uniform dispersion of the flour in the water and to prevent the formation of lumps. Subsequently, up to 300 mL of distilled water was added, and the mixture was cooked at an average temperature of 150°C and 1500 rpm for approximately 25 min. This resulted in two types of well‐mixed porridges: nonroasted porridge (NRP) and roasted porridge (RP).

In addition to the porridge samples, cleaned pulses were cooked by boiling to create BP samples. Pulses were soaked in distilled water for 12 h at room temperature using a 1:3 seed‐to‐water ratio. After draining the soaking water, pulses were boiled in distilled water, maintaining the same seed‐to‐water ratio (1:3), using a portable induction cooktop (1800 W Portable Induction Cooktop; Duxtop, Durham, NC, USA). Cooking times varied by cultivar: Otebo (16 min), Navy (24 min), Great Northern (23 min), White Kidney (30 min), Chickpea (45 min), Manteca (20 min), Mayacoba (33 min), and Cranberry (50 min) and were determined using a Mattson pin drop cooker (Department of Physics and Astronomy Machine Shop, Michigan State University, East Lansing, MI, USA).

### 2.2 Solid‐Phase Microextraction

NRP, RP, and BP samples were prepared fresh on the day of analysis, immediately transferred to 20 mL glass headspace vials to minimize volatile loss, and analyzed within 8 h of preparation. The following sample quantities for each of the eight pulses were individually placed into vials: 2 g of NRF, 2 g of RF, 5 g of mashed BP, 5 g of NRP (mixed with 1 g NaCl), and 5 g of RP (mixed with 1 g NaCl). Flour samples (NRF and RF) were analyzed as dry powders without the addition of water or salt, as methodology development indicated that this approach yielded better peak resolution. However, adding salt to the porridges during SPME induces a “salting‐out” effect, which lowers the partitioning coefficient (*K*) for some analytes and increases their concentration in the headspace, thereby enhancing extraction efficiency for polar compounds and organic volatiles (Westland 2021). For increased volatilization of compounds, the samples were first held at 50°C for 30 min in a water bath. Following this period, the SPME fiber (carboxen/polydimethylsiloxane/divinylbenzene [CAR/PDMS/DVB]) 2 cm, 30/50 µm (Supelco, Sigma–Aldrich; St. Louis, MO, USA) was introduced into the headspace of each sample and exposed for an additional 30 min at 50°C in the same water bath.

#### 2.2.1 Gas Chromatography–Mass Spectrometry Analysis

A gas chromatograph and mass spectrometer were used to separate and detect the headspace aroma compounds and to collect detection frequency data on separated aroma compounds. Absorbed volatiles were desorbed for 20 s from the fiber coating by inserting the SPME fiber through a predrilled septum (Thermogreen LB‐2, Supelco Co., Bellefonte, PA, USA) and into a glass‐lined, split/splitless injector port (200°C) of a gas chromatograph (Agilent 6890 Gas Chromatograph, Hewlett‐Packard Co., Wilmington, DE, USA) set at a splitless front inlet mode. Volatiles were separated on a 30 m × 0.25 mm i.d. capillary column (HP‐5, Hewlett‐Packard) having a film thickness of 0.25 µm. Ultra‐purified helium (99.999%) was used as carrier gas at a ramped flow with an initial flow rate of 1.2 mL/min held for 1 min and then increased at a rate of 1 mL/min to a final flow rate of 1.8 mL/min. The initial linear velocity was 44 cm/s. The initial temperature of the GC oven was 32°C; it was held for 0.25 min, increased to 60°C at a rate of 20°C/min, and again increased to 150°C at a rate of 50°C/min, and finally increased to 280°C at a rate of 70°C/min, and held for 2 min. The total analysis time was 7.4 min and transfer line temperature was 225°C.

Volatile detection was done using time of flight mass spectrometry (TOFMS) with an electron ionization source (LECO Pegasus III Mass Spec, Leco Corp, St. Joseph, MI, USA). For detection with mass spectrometry, the ion source was held at 200°C with electron energy at 70 eV and a scan range of 29–400 mass units; the scan rate was 20 spectra per s with an acquisition voltage of 1500–1600 V. Preliminary identification of volatiles was performed by comparison of their mass spectra with those of authenticated chemical standards. During the experiment, each volatile compound of interest was identified either by the National Institute of Standards and Technology (NIST) database (V.05) through a mass spectra library search or by comparing retention times (RTs) and the mass spectra of the compounds with those of the pure commercial standards (as listed in the following section).

The identified volatile compounds were classified into eight chemical classes: aldehydes, alkanes, alcohols, ketones, terpenoids, sulfurous compounds, nitrogenous compounds, and aromatic compounds. Volatile compound identification was performed using two levels of annotation. Level 1 identification involved comparing the RTs of volatile compounds with those of authentic chemical standards. Level 2 identification involved putative annotation of metabolites based on spectral similarity to public or commercial spectral libraries without the use of chemical reference standards (Sumner et al. 2007).

The metabolites identified by level 2 annotation were quantified by calculating the peak areas of each volatile based on the average area under the curve (AUC) from triplicate measurements and reported for a single *m*/*z* (mass‐to‐charge ratio) using the unique mass. The quantification of volatiles identified by level 1 annotation was achieved by estimating the volatile concentration of each compound in a sample using the area ratio method. The AUC of volatiles in a sample was compared to the peak areas of a 25‐component external standard mixture prepared at 0.2 µL in 4.4 L (Park et al. 2024). For a volatile compound with known density *ρ* in g/mL and molar mass M in g/mol, the molar concentration of the standard $C_{\mathrm{standard}}\;$ in mol/L was calculated as:

$$C_{\mathrm{standard}}=\frac{\rho \;\times \;0.2\;\mu L}{M\;\times \;4.4\;L\times 25}$$

The estimated concentration of the volatile in the sample $C_{\mathrm{sample}}$ in mol/L was determined by the area ratio of the sample to the standard as follows:


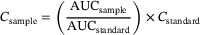




The final estimated concentration of a volatile compound was calculated by taking the average of the triplicate estimated volatile concentrations in a sample.

#### 2.2.2 Standards

Authenticated pure commercial standards of 2‐butanone, 2‐methyl butanal, butanol, 2‐ethylfuran, 3‐methylbutanol, dimethyl disulfide, 1‐pentanol, hexanal, (E)‐2‐hexenal, 1‐hexanol, o‐xylene, 2‐heptanone, styrene, heptanal, methional, 2,5‐dimethyl pyrazine, benzaldehyde, 1‐octen‐3‐ol, 6‐methyl‐5‐hepten‐2‐one, octanal, decane, L‐limonene, nonanal, decanal, and geosmin purchased from Sigma‐Aldrich (St. Louis, MO, USA) were combined in equal volume aliquots to create a twenty five‐component mixture. Every week, 0.2 µL of the mixture was injected on a glass microfiber filter and placed in a glass volumetric flask of 4.4 L fitted with a specially made ground glass stopper containing a gastight Mininert valve (Alltech Associates, Inc., Deerfield, IL, USA). The flask was held at 22°C until the liquid standards were fully volatilized (Song et al. 1998).

### 2.3 Statistical Analysis

The GC–MS AUC and estimated volatile concentration data were analyzed using R statistical computing software (version 4.2.2; R Core Team 2022) to assess sample differences. All analyses were conducted in triplicate for each sample, and a blank run was performed after each sample run. Analysis of variance (ANOVA) was conducted using the agricolae v. 1.3.5 (de Mendiburu 2021) package, followed by least significant difference (LSD) post hoc multiple comparisons tests (*α* = 0.05). For multivariate analysis, principal component analysis (PCA) and hierarchical cluster analysis (HCA) were conducted using the FactoMineR v. 2.8 (Lê et al. 2008) package. Visualization of PCA results was carried out using ggplot2 v. 3.5.1 (Wickham 2016). Heatmaps were generated using the pheatmap v. 1.0.12 (Kolde 2018) package.

A three‐factor ANOVA was used to evaluate the effects of cultivar, processing, and year on the total estimated volatile concentrations of all pulse samples from harvest years 2022 and 2023. The model included two‐way interactions (cultivar:year, cultivar:processing, and year:processing) and a three‐way interaction (cultivar:year:processing), enabling the evaluation of how these factors and their interplay influenced volatile profiles. For all statistical tests, an *α* of 0.05 was used to determine statistical significance.

To further evaluate the impact of processing treatments on volatile concentrations across key chemical classes, a separate ANOVA model was applied. The volatiles were grouped by their chemical class for each pulse type from its respective harvest year, and the total estimated volatile concentration per class was calculated by summing all volatiles for each of the chemical classes. One‐way ANOVA was conducted with processing as the independent variable, followed by an LSD post hoc test to identify pairwise differences. To identify significant differences in volatile concentrations across samples within a given year, a one‐way ANOVA was conducted for each targeted volatile compound, followed by a LSD post hoc test to determine pairwise differences. Results from the 2022 and 2023 harvest years are reported in Tables 2 and S1, respectively.

**TABLE 2:** Summary of analysis of variance (ANOVA) results in a three‐way ANOVA evaluating the effects of cultivar, year, processing, and their interactions on the total volatile concentrations from HS–SPME–GC–MS analysis.

**Interaction**	**Df**	**SS**	**MS**	***F*‐value**	***p*‐value**
Cultivar	7	1.2E−13	1.7E−14	1.7	0.113
Year	1	5.2E−13	5.2E−13	50.8	3.3E−11
Processing	4	1.1E−12	2.8E−13	27.5	2.2E−17
Cultivar:year	7	1.7E−13	2.4E−14	2.4	2.5E−02
Cultivar:processing	28	7.6E−13	2.7E−14	2.6	8.1E−05
Year:processing	4	6.0E−13	1.5E−13	14.6	3.4E−10
Cultivar:year:processing	28	8.7E−13	3.1E−14	3.0	7.0E−06
Residuals	160	1.6E−12	1.0E−14		

Abbreviations: Df, degrees of freedom; SS, sum of squares; MS, mean sum of squares.

To investigate broader patterns in volatile content across two growing seasons from 2022 and 2023, estimated volatile concentration data were mean‐centered and normalized prior to PCA analysis. HCA analysis was also applied to cluster samples into subgroups with shared volatile profiles.

For heatmap visualizations, the peak areas of volatiles from the 2022 harvest year reported in Table S2, identified through level 1 and level 2 annotation was log‐transformed to emphasize differences in volatile compound profiles across cultivars in NRF and NRP samples (Zhang et al. 2025).

## 3 Results and Discussion

**FIGURE 2:**
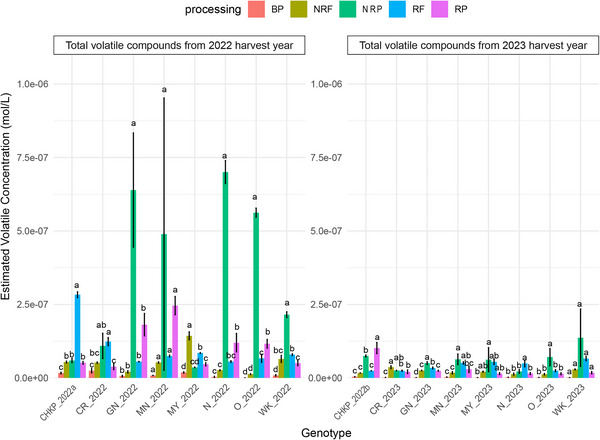
Total estimated volatile concentration of a market sample of Chickpea (CHKP) obtained commercially, harvested in 2022 and 7 bean varieties grown in Michigan during the harvest years 2022 and 2023, namely, Cranberry (CR), Great Northern (GN), Manteca (MN), Mayacoba (MY), Navy (N), Otebo (O), and White Kidney (WK). Samples marked 2022 and 2022a were analyzed in April 2024; samples marked 2023 and 2022b were analyzed from August through September 2024. Volatile concentrations are presented for five processing treatments: boiled pulses (BP), nonroasted flour (NRF), nonroasted porridge (NRP), roasted flour (RF), and roasted porridge (RP). Results represent the average values from triplicate measurements. Mean values for each pulse type that do not share a letter are significantly different (*p* < 0.05) as determined by the LSD post hoc comparison test.

**FIGURE 3:**
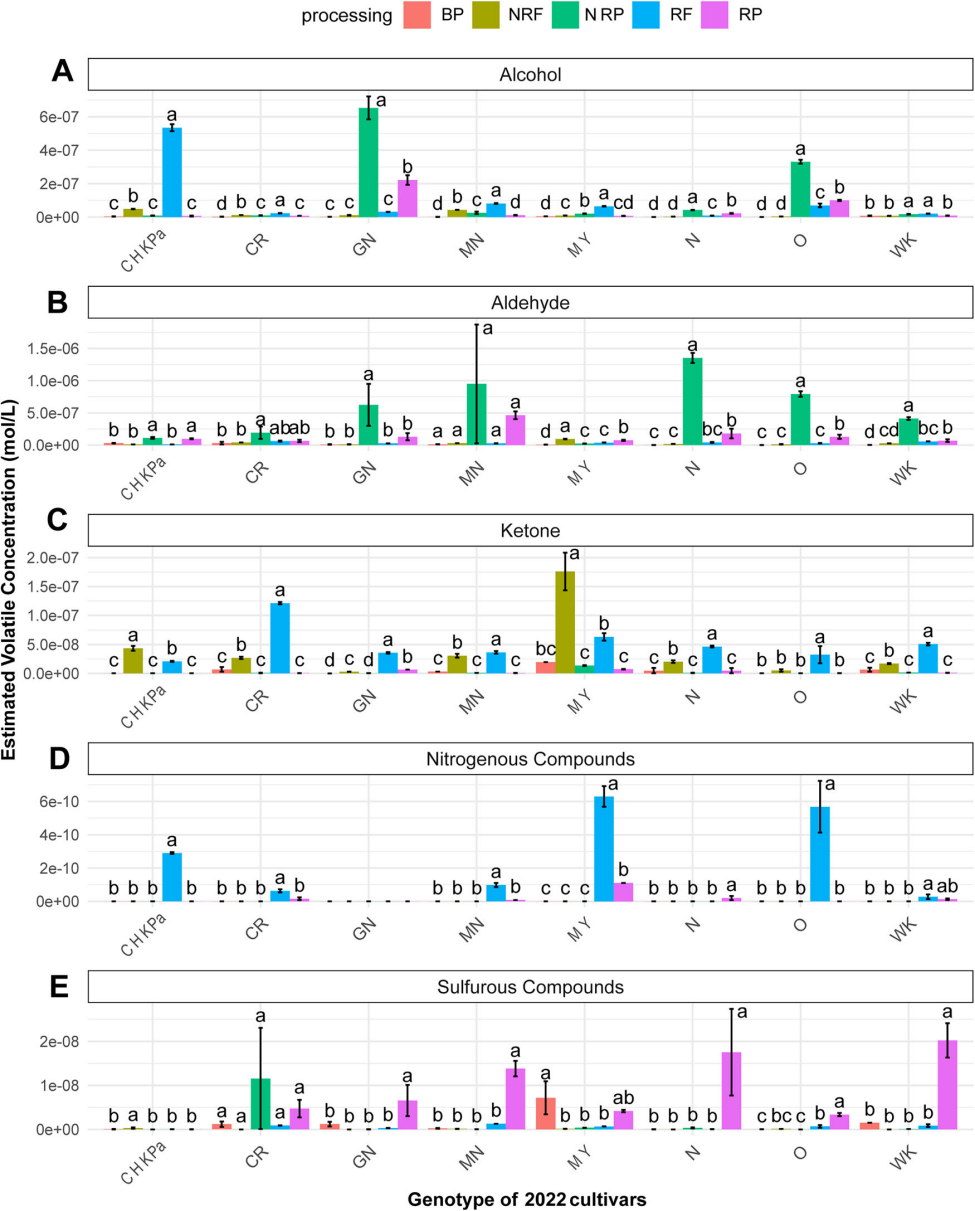
Effect of processing (roasting; boiling) on the estimated volatile concentration of (A) alcohols; (B) aldehyde; (C) ketone; (D) nitrogenous compound; (E) sulfurous compound on a market sample‐Chickpea (CHKPa) obtained commercially and seven bean varieties grown in Michigan and all harvested in 2022, namely, Cranberry (CR), Great Northern (GN), Manteca (MN), Mayacoba (MY), Navy (N), Otebo (O), and White Kidney (WK) in boiled pulses (BP), nonroasted flour (NRF), nonroasted porridge (NRP), roasted flour (RF), and roasted porridge (RP). These samples were analyzed in April 2024. Results are the average value from triplicates. For each type of pulse, mean values that do not share a letter are significantly different (*p* < 0.05) as per the LSD post hoc comparison test.

A total of 32 volatile compounds were identified across the pulse samples, 25 of which were annotated as level 1 and quantified using authentic chemical standards and visualized in Figures 2, 3, and 5. (E)‐2‐Hexenal and 1‐hexanol were the most abundant volatiles. The identified volatiles included alcohols (5), aldehydes (8), ketones (3), aromatics (4), terpenoids (1), alkanes (1), nitrogenous compounds (1), and sulfurous compounds (2). Consistent with previous studies on legumes (Khrisanapant et al. 2022; Mishra et al. 2017; Oomah et al. 2007), targeted GC–MS identified alcohols (18.2%), aldehydes (51.7%), and ketones (21.6%) as the most abundant chemical classes as an average across all samples. While volatile profiles varied significantly across samples, processing treatment had the most significant impact (*p* < 0.001), followed by the effect of harvest year. In contrast, cultivar alone did not significantly affect overall volatile concentrations (Table 2).

### 3.1 Effect of Processing on Volatile Profiles

**FIGURE 4:**
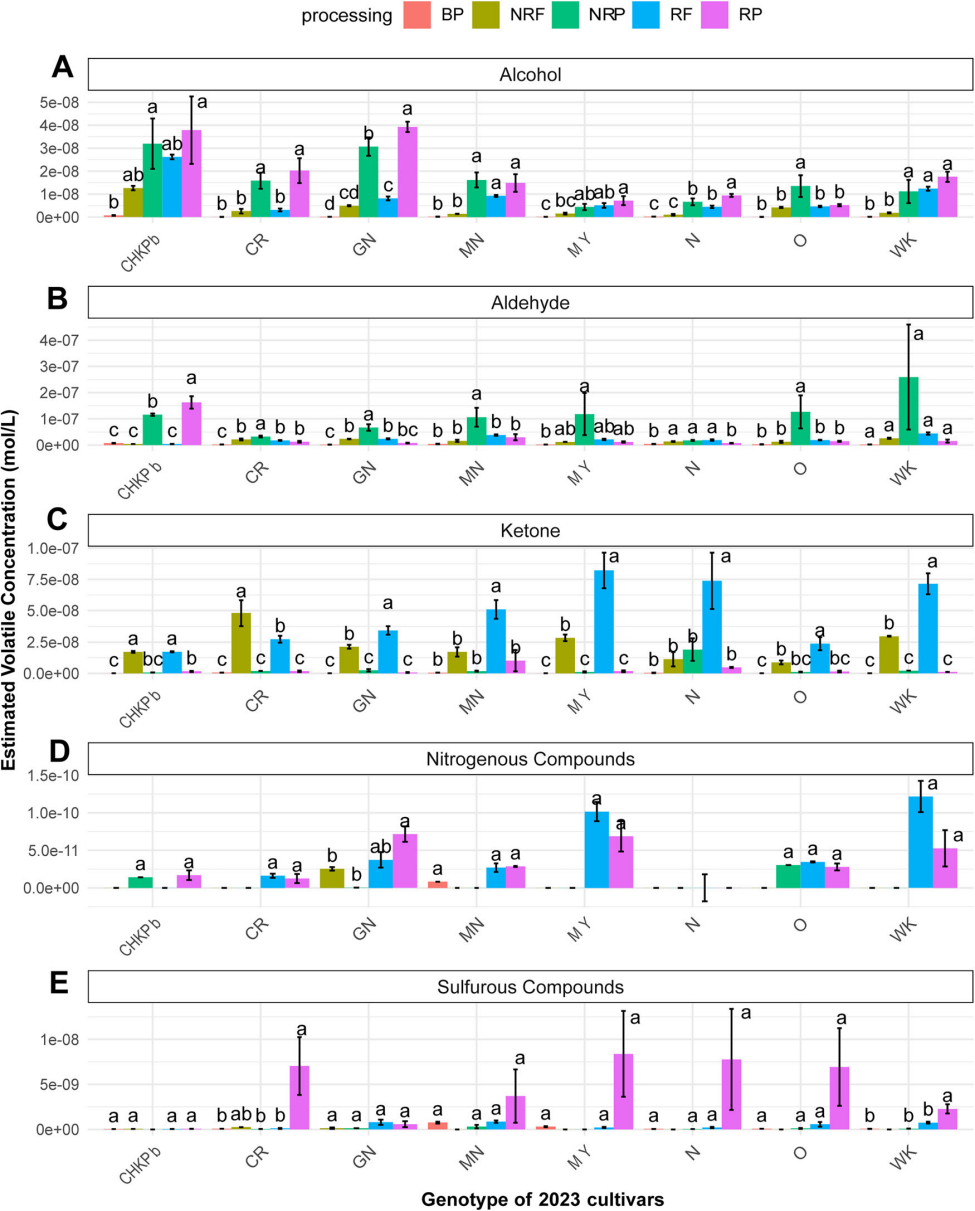
Effect of processing (roasting; boiling) on the estimated volatile concentration of (A) alcohols; (B) aldehyde; (C) ketone; (D) nitrogenous compound; (E) sulfurous compound on a market sample‐Chickpea (CHKPb) obtained commercially and seven bean varieties grown in Michigan and all harvested in 2023, namely, Cranberry (CR), Great Northern (GN), Manteca (MN), Mayacoba (MY), Navy (N), Otebo (O), and White Kidney (WK) in boiled pulses (BP), nonroasted flour (NRF), nonroasted porridge (NRP), roasted flour (RF), and roasted porridge (RP). These samples were analyzed August through September 2024. Results are the average value from triplicates. For each type of pulse, mean values that do not share a letter are significantly different (*p* < 0.05) as per the LSD post hoc comparison test.

Figures 3 and 4 illustrate how the thermal treatment of pulses affects the distribution of volatile compounds across the harvest years 2022 and 2023, respectively. The ANOVA results indicated that processing significantly affected total volatile concentration (*p* < 0.05; Table 2).

#### 3.1.1 Effect of Roasting

##### 3.1.1.1 Roasted Flour

Alcohols and ketones were the most abundant volatiles in roasted flour, accounting for an average of 37% and 30% of the total estimated volatile content, respectively. In both harvest years generally, ketones and alcohols increased slightly, and pyrazines increased substantially in RF compared to NRF, depending on cultivar type (Figures 3A,C,E and  4A,C,E). Although, a significant increase in the total estimated volatile concentration after roasting was only observed in RF samples from the 2022 harvest of (CHKP, CR) and white‐colored (O) pulse cultivars compared to NRF (Figure 2).

Interestingly, this increase could be attributed to the formation of a new group of volatile compounds, characterized by a significantly higher concentration of pyrazines due to roasting, in RF samples (Figure 3D). These compounds were absent from NRF samples, indicating that pyrazines are primarily formed during roasting. In harvest years 2022 and 2023, roasting significantly increased the concentration of nitrogenous compounds in RF samples of yellow‐colored (MN, MY), white‐colored (O, WK), and other (CHKP, CR), pulse cultivars, compared to NRF (Figures 3D and 4D). A fivefold increase in nitrogenous compounds was observed in CHKP, MY, and O cultivars (Table 3). Nitrogenous compounds, particularly pyrazines, often impart chocolate, roasted nutty, and sharp flavors to pulses (Azarnia et al. 2011). Particularly, 2,5‐dimethyl pyrazine, characterized by a nutty, roasted, musty, and grassy aroma was the most abundant in RF samples of yellow‐colored (MN, MY), white‐colored (O, WK), and other pulses (CHKP, CR; Tables 3 and S1; The Good Scents Company 2025).

**TABLE 3:** Estimated concentration in nmol/L of volatiles quantified using authentic chemical standards across nonroasted flour (NRF), roasted flour (RF), nonroasted porridge (NRP), roasted porridge (RP), and boiled pulses (BP) from the seven bean varieties (Great Northern, White Kidney, Navy, Otebo, Manteca, Mayacoba, and Cranberry) grown in Michigan with a harvest year from 2022 and a market sample of Chickpea obtained commercially (harvested in 2022).

**White‐colored beans (estimated volatile concentration in nmol/L)**											
	**Great northern bean**					**White kidney bean**					
**Compound name**	**NRF**	**RF**	**NRP**	**RP**	**BP**	**NRF**	**RF**	**NRP**	**RP**	**BP**	Odor profiles from The Good Scents Company (2025)
**Aldehyde**											
2‐Methyl butanal	1.6 ghijkl	3.9 efgh	1.0 hijkl	2.6 fghijkl	nd	1.2 ghijkl	11.0 bc	170.0 a	1.7 ghijkl	0.53 jkl	Malty, musty, fermented
Hexanal	0.89 klm	4.8 efghi	6.2 defgh	0.34 m	0.32 m	5.0 efghi	7.4 cdefg	9.1 bcd	0.47 m	0.12 m	Vegetable, aldehydic, clean
(E)‐2‐Hexenal	1.5 f	2.2 f	300.0 bcd	60.0 ef	3.2 f	3.2 f	3.9 f	22.0 ef	31.0 ef	nd	Sweet, vegetable, bitter almond
Heptanal	0.14 fghijk	0.32 def	0.2 fghi	0.039 ijk	nd	0.42 cde	0.98 a	0.42 cde	0.039 ijk	nd	Aldehydic, fatty, herbal
Benzaldehyde	0.18 qrs	0.5 klmno	1.5 c	0.58 ijklmn	0.58 ijklmn	0.52 klmno	0.91 ef	0.68 ghijk	0.4 mnopq	0.21 pqrs	Sweet, cherry, nutty
Octanal	0.17 ghijk	0.26 defgh	0.066 kl	0.02 kl	nd	0.39 cde	0.78 b	0.061 kl	0.026 kl	0.0074 L	Aldehydic, fatty, herbal
Nonanal	1.1 gh	1.1 g	0.37 lmno	0.14 no	0.16 mno	2.1 d	3.1 ab	0.59 ijkl	0.16 mno	0.079 o	Aldehydic, fatty, rose
Decanal	0.081 b	0.097 b	0.05 b	0.021 b	0.029 b	0.24 b	0.33 b	0.064 b	0.019 b	nd	Sweet, aldehydic, floral
**Alcohol**											
Butanol	0.84 fg	0.7 fg	5.4 e	nd	nd	1.1 fg	1.0 fg	5.1 e	0.32 fg	1.2 fg	Sweet, fermented, oily
3‐Methylbutanol	1.4 fghi	12.0 a	5.4 c	3.4 d	0.31 hij	0.82 ghij	7.2 b	0.87 ghij	0.6 hij	0.6 hij	Musty, vegetable, cocoa
1‐Pentanol	2.1 c	1.8 cde	0.7 gh	0.095 jk	nd	1.1 fg	1.4 ef	0.34 hijk	0.019 k	0.041 jk	Sweet, fermented, yeasty
1‐Hexanol	0.95 h	0.54 h	310.0 a	110.0 d	0.38 h	0.44 h	0.35 h	1.7 h	2.3 gh	1.7 h	Sweet, pungent, herbal
1‐Octen‐3‐ol	0.13 lmn	0.16 lmn	2.0 ab	0.57 hijkl	0.057 mn	0.11 lmn	0.083 mn	0.4 ijklmn	1.1 efg	0.17 klmn	Vegetable, mushroom, chicken
**Ketone**											
2‐Butanone	1.3 kl	17.0 defg	0.12 L	3.2 jkl	nd	8.1 hijkl	25.0 cd	0.72 L	0.47 L	3.1 jkl	Camphoreous, acetone, fruity
2‐Heptanone	0.099 fghi	0.31 b	0.0082 j	nd	nd	0.2 cde	0.44 a	nd	0.0091 j	0.077 ghij	Sweet, spicy, banana
6‐Methyl‐5‐hepten‐2‐one	0.021 cdef	nd	nd	nd	nd	0.041 cdef	0.046 cdef	nd	0.016 cdef	nd	Musty, banana, fruity
**Aromatic compounds**											
2‐Ethylfuran	0.066 gh	0.29 efgh	0.13 fgh	0.014 h	0.35 efgh	0.42 efgh	1.5 cd	0.36 efgh	0.21 fgh	0.17 fgh	Malty, cocoa, nutty
o‐Xylene	7.6 cde	8.2 cde	nd	nd	0.41 e	37.0 a	13.0 bc	nd	1.4 e	nd	Geranium
Styrene	0.71 c	0.61 cd	nd	nd	nd	1.9 a	1.2 b	nd	nd	nd	Sweet, plastic, floral
Geosmin	nd	nd	nd	nd	nd	nd	nd	nd	nd	nd	Musty, earthy, fresh
**Terpenoids**											
L‐Limonene	0.078 cd	0.0077 e	nd	nd	nd	0.012 e	0.014 e	nd	nd	nd	Camphoreous, herbal, terpenic
**Alkanes**											
Decane	0.054 a	nd	nd	nd	nd	0.0034 efgh	0.0019 fgh	nd	0.002 efgh	0.0062 cde	Unknown
**Sulfur compounds**											
Dimethyl disulfide	nd	0.15 e	0.0062 e	3.3 cde	0.6 e	nd	0.43 e	0.03 e	10.0 a	0.76 e	Vegetable, onion, cabbage
Methional	nd	nd	nd	nd	nd	nd	nd	nd	0.033 a	nd	Cabbage, pungent
**Nitrogen compounds**											
2,5‐Dimethyl pyrazine	nd	nd	nd	nd	nd	nd	0.014 de	0.0 e	0.0066 e	nd	Nutty, peanut, musty

**White‐colored beans (estimated volatile concentration in nmol/L)**											
	**Navy bean**					**Otebo bean**					
**Compound name**	**NRF**	**RF**	**NRP**	**RP**	**BP**	**NRF**	**RF**	**NRP**	**RP**	**BP**	Odor profiles from The Good Scents Company (2025)
**Aldehyde**											
2‐Methyl butanal	1.7 ghijkl	4.0 efg	0.058 L	0.0075 L	nd	2.6 fghijkl	2.5 fghijkl	3.6 fghi	0.1 L	0.0 L	Malty, musty, fermented
Hexanal	4.3 ghijkl	11.0 bc	3.2 hijklm	0.3 m	0.25 m	1.6 ijklm	6.2 defgh	4.9 efghi	1.6 ijklm	0.18 m	Vegetable, aldehydic, clean
(E)‐2‐Hexenal	nd	nd	670.0 a	89.0 def	nd	0.32 f	0.3 f	390.0 bc	61.0 ef	0.0 f	Sweet, vegetable, bitter almond
Heptanal	0.49 cd	1.1 a	0.26 efgh	0.022 ijk	0.047 ijk	0.19 fghij	1.1 a	0.31 defg	0.13 hijk	0.0094 jk	Aldehydic, fatty, herbal
Benzaldehyde	0.34 opqr	0.81 fgh	0.43 lmnop	0.25 pqrs	0.092 s	0.16 rs	0.9 efg	0.82 fgh	0.8 fghi	0.057 s	Sweet, cherry, nutty
Octanal	0.36 cdef	0.64 b	0.027 kl	0.0089 kl	0.061 kl	0.14 ghijkl	0.27 defg	0.11 ghijkl	0.086 ijkl	nd	Aldehydic, fatty, herbal
Nonanal	nd	nd	nd	nd	nd	nd	nd	nd	nd	nd	Aldehydic, fatty, rose
Decanal	0.086 b	0.19 b	0.028 b	0.0099 b	0.012 b	0.069 b	0.1 b	0.036 b	0.045 b	0.008 b	Sweet, aldehydic, floral
**Alcohol**											
Butanol	0.44 fg	0.4 fg	nd	0.058 g	0.018 g	0.37 fg	21.0 a	nd	0.13 g	nd	Sweet, fermented, oily
3‐Methylbutanol	nd	2.3 def	0.24 ij	1.9 efg	nd	0.52 hij	1.9 efg	0.065 j	0.17 ij	nd	Musty, vegetable, cocoa
1‐Pentanol	0.54 hij	1.1 fg	0.32 hijk	0.21 hijk	nd	0.25 hijk	0.25 hijk	0.3 hijk	0.046 jk	nd	Sweet, fermented, yeasty
1‐Hexanol	0.13 h	0.17 h	19.0 fg	8.3 fgh	0.084 h	0.35 h	11.0 fgh	160.0 c	49.0 e	nd	Sweet, pungent, herbal
1‐Octen‐3‐ol	0.062 mn	0.21 jklmn	1.2 efg	0.63 hijk	0.035 mn	0.071 mn	0.8 ghi	1.3 def	0.65 hij	nd	Vegetable, mushroom, chicken
**Ketone**											
2‐Butanone	10.0 ghij	23.0 cde	0.33 L	2.3 jkl	2.4 jkl	2.4 jkl	16.0 efgh	nd	0.13 L	0.13 L	Camphoreous, acetone, fruity
2‐Heptanone	0.12 efgh	0.17 cdef	0.019 ij	0.0048 j	nd	0.0099 j	0.24 bc	0.0091 j	nd	nd	Sweet, spicy, banana
6‐Methyl‐5‐hepten‐2‐one	0.02 cdef	0.07 c	nd	nd	0.0016 f	nd	nd	nd	nd	nd	Musty, banana, fruity
**Aromatic compounds**											
2‐Ethylfuran	0.14 fgh	0.33 efgh	2.2 b	0.43 efgh	0.067 gh	0.085 fgh	1.1 d	0.16 fgh	0.034 h	0.029 h	Malty, cocoa, nutty
o‐Xylene	5.8 cde	6.9 cde	nd	7.7 cde	nd	2.8 e	0.048 e	nd	nd	nd	Geranium
Styrene	0.34 ef	0.32 efg	nd	nd	nd	0.0046 i	0.46 de	nd	nd	nd	Sweet, plastic, floral
Geosmin	nd	nd	nd	nd	nd	nd	nd	nd	nd	nd	Musty, earthy, fresh
**Terpenoids**											
L‐Limonene	0.054 cde	0.013 e	nd	nd	nd	nd	0.024 de	nd	0.042 de	nd	Camphoreous, herbal, terpenic
**Alkanes**											
Decane	nd	nd	nd	nd	nd	nd	0.03 b	nd	nd	nd	Unknown
**Sulfur compounds**											
Dimethyl disulfide	nd	0.027 e	0.17 e	8.8 ab	0.0071 e	0.05 e	0.34 e	0.0025 e	1.7 e	0.0089 e	Vegetable, onion, cabbage
Methional	nd	nd	nd	0.0012 c	nd	nd	0.0043 bc	nd	nd	nd	Cabbage, pungent
**Nitrogen compounds**											
2,5‐Dimethyl pyrazine	nd	nd	nd	0.0099 e	nd	nd	0.28 a	nd	nd	nd	Nutty, peanut, musty

**Yellow‐colored beans (estimated volatile concentration in nmol/L)**											
	**Manteca**					**Mayacoba**					
**Compound name**	**NRF**	**RF**	**NRP**	**RP**	**BP**	**NRF**	**RF**	**NRP**	**RP**	**BP**	Odor profiles from The Good Scents Company (2025)
**Aldehyde**											
2‐Methyl butanal	2.6 fghijkl	4.0 efg	0.77 ijkl	1.6 ghijkl	1.2 ghijkl	12.0 b	5.3 ef	1.3 ghijkl	6.6 de	0.5 kl	Malty, musty, fermented
Hexanal	9.0 bcd	6.3 defgh	4.5 fghijk	2.0 ijklm	1.8 ijklm	4.7 fghij	8.5 bcde	1.9 ijklm	0.84 klm	0.72 lm	Vegetable, aldehydic, clean
(E)‐2‐Hexenal	0.25 f	0.14 f	470.0 ab	230.0 cde	0.54 f	25.0 ef	0.44 f	5.0 f	26.0 ef	0.0 f	Sweet, vegetable, bitter almond
Heptanal	0.69 b	0.49 cd	0.13 ghijk	0.13 ghijk	0.15 fghijk	0.48 cd	1.1 a	0.08 hijk	0.093 hijk	0.099 hijk	Aldehydic, fatty, herbal
Benzaldehyde	nd	nd	nd	nd	nd	nd	nd	nd	nd	nd	Sweet, cherry, nutty
Octanal	0.23 fghij	0.25 efgh	0.051 kl	0.059 kl	0.12 ghijkl	2.1 a	0.43 c	0.24 efghi	0.41 cd	0.077 jkl	Aldehydic, fatty, herbal
Nonanal	1.0 ghi	1.3 fg	0.28 lmno	0.39 lmno	0.98 ghij	1.2 fg	1.7 de	0.14 no	0.31 lmno	0.6 ijkl	Aldehydic, fatty, rose
Decanal	0.043 b	0.11 b	0.032 b	0.046 b	0.08 b	0.06 b	0.12 b	0.014 b	0.043 b	0.035 b	Sweet, aldehydic, floral
**Alcohol**											
Butanol	8.9 d	18.0 b	2.6 f	nd	0.033 g	2.1 fg	15.0 c	0.33 fg	0.87 fg	0.35 fg	Sweet, fermented, oily
3‐Methylbutanol	0.12 j	4.6 c	0.77 ghij	1.2 fghij	nd	0.2 ij	2.9 de	1.5 fgh	0.35 hij	0.59 hij	Musty, vegetable, cocoa
1‐Pentanol	0.13 jk	0.38 hijk	0.17 ijk	0.056 jk	nd	0.13 jk	0.27 hijk	0.35 hijk	0.041 jk	0.12 jk	Sweet, fermented, yeasty
1‐Hexanol	9.9 fgh	16.0 fgh	7.2 fgh	3.0 gh	0.24 h	1.6 h	13.0 fgh	6.2 fgh	1.5 h	1.0 h	Sweet, pungent, herbal
1‐Octen‐3‐ol	2.3 a	0.75 ghi	1.6 bcde	1.2 efg	0.11 lmn	0.41 ijklmn	1.5 cde	1.7 bcd	0.49 ijklm	0.15 lmn	Vegetable, mushroom, chicken
**Ketone**											
2‐Butanone	15.0 efghi	18.0 defg	0.34 L	0.27 L	1.6 jkl	88.0 a	31.0 c	6.7 ijkl	3.6 jkl	9.8 ghijk	Camphoreous, acetone, fruity
2‐Heptanone	0.18 cdef	0.23 bcd	0.013 j	0.014 ij	nd	0.025 ij	0.24 bc	0.022 ij	0.013 j	0.045 hij	Sweet, spicy, banana
6‐Methyl‐5‐hepten‐2‐one	nd	nd	0.059 cde	0.021 cdef	nd	0.027 cdef	nd	nd	nd	nd	Musty, banana, fruity
**Aromatic compounds**											
2‐Ethylfuran	0.66 e	1.7 c	0.35 efgh	0.31 efgh	0.26 efgh	2.3 b	1.3 cd	6.8 a	0.53 ef	0.27 efgh	Malty, cocoa, nutty
o‐Xylene	0.04 e	0.021 e	1.5 e	1.8 e	nd	1.6 e	0.035 e	nd	0.045 e	nd	Geranium
Styrene	0.59 cd	0.11 ghi	nd	nd	nd	0.019 i	0.25 fgh	nd	nd	nd	Sweet, plastic, floral
Geosmin	nd	nd	nd	nd	nd	nd	nd	nd	nd	nd	Musty, earthy, fresh
**Terpenoids**											
L‐Limonene	0.11 bc	0.018 de	nd	nd	nd	0.14 b	0.026 de	nd	nd	nd	Camphoreous, herbal, terpenic
**Alkanes**											
Decane	0.0 h	0.01 c	nd	nd	nd	0.0045 efg	nd	0.0051 defg	nd	0.0055 def	Unknown
**Sulfur compounds**											
Dimethyl disulfide	0.065 e	0.64 e	0.011 e	6.9 abc	0.11 e	0.066 e	0.34 e	0.19 e	2.1 de	3.6 cde	Vegetable, onion, cabbage
Methional	nd	0.0031 bc	nd	nd	nd	nd	nd	nd	0.0081 b	nd	Cabbage, pungent
**Nitrogen compounds**											
2,5‐Dimethyl pyrazine	nd	0.049 cd	nd	0.0044 e	nd	nd	0.31 a	nd	0.056 c	nd	Nutty, peanut, musty

**Other pulses (estimated volatile concentration in nmol/L)**											
	**Chickpea 2022^a^**					**Cranberry**					
**Compound name**	**NRF**	**RF**	**NRP**	**RP**	**BP**	**NRF**	**RF**	**NRP**	**RP**	**BP**	Odor profiles from The Good Scents Company (2025)
**Aldehyde**											
2‐Methyl butanal	3.1 fghijk	1.7 ghijkl	3.4 fghij	nd	0.087 L	1.8 ghijkl	9.2 cd	0.2 kl	0.44 kl	1.2 ghijkl	Malty, musty, fermented
Hexanal	0.71 lm	1.6 ijklm	49.0 a	45.0 a	0.85 klm	8.2 cdef	12.0 b	0.97 jklm	0.47 m	3.7 hijklm	Vegetable, aldehydic, clean
(E)‐2‐Hexenal	nd	nd	0.5 f	0.44 f	nd	5.1 f	3.8 f	94.0 def	30.0 ef	9.7 ef	Sweet, vegetable, bitter almond
Heptanal	nd	0.2 fghi	0.45 cd	0.47 cd	0.12 hijk	0.51 bc	0.69 b	0.024 ijk	0.028 ijk	0.044 ijk	Aldehydic, fatty, herbal
Benzaldehyde	0.64 hijkl	0.64 hijkl	0.43 lmnop	0.38 nopqr	0.35 nopqr	0.55 jklmno	0.63 hijklm	0.34 opqr	0.4 nopq	0.2 qrs	Sweet, cherry, nutty
Octanal	0.15 ghijkl	0.099 hijkl	0.14 ghijkl	0.14 ghijkl	0.055 kl	0.62 b	0.65 b	0.013 kl	0.018 kl	0.033 kl	Aldehydic, fatty, herbal
Nonanal	0.08 o	0.6 ijkl	1.5 ef	1.6 ef	0.25 lmno	2.9 bc	3.6 a	0.64 hijkl	0.66 hijkl	0.42 lmno	Aldehydic, fatty, rose
Decanal	0.0099 b	0.054 b	0.045 b	0.054 b	13.0 a	0.27 b	0.34 b	0.014 b	0.022 b	0.021 b	Sweet, aldehydic, floral
**Alcohol**											
Butanol	1.5 fg	9.3 d	nd	nd	nd	0.87 fg	0.91 fg	nd	nd	nd	Sweet, fermented, oily
3‐Methylbutanol	0.14 j	0.44 hij	nd	nd	0.96 ghij	1.0 ghij	5.9 c	1.0 ghij	1.0 ghij	0.28 ij	Musty, vegetable, cocoa
1‐Pentanol	0.65 ghi	1.4 def	2.9 b	1.9 cd	0.079 jk	3.1 b	4.2 a	0.36 hijk	0.034 k	nd	Sweet, fermented, yeasty
1‐Hexanol	22.0 f	260.0 b	0.57 h	0.63 h	0.51 h	0.78 h	0.37 h	1.4 h	1.4 h	0.32 h	Sweet, pungent, herbal
1‐Octen‐3‐ol	0.37 ijklmn	0.97 fgh	0.66 hij	0.44 ijklmn	0.12 lmn	0.15 lmn	0.11 lmn	1.8 bc	1.1 efg	0.23 jklmn	Vegetable, mushroom, chicken
**Ketone**											
2‐Butanone	22.0 def	10.0 ghij	nd	nd	nd	13.0 fghi	60.0 b	0.29 L	0.19 L	3.4 jkl	Camphoreous, acetone, fruity
2‐Heptanone	0.015 ij	0.15 defg	0.055 hij	0.075 ghij	0.036 hij	nd	0.15 defg	0.012 j	nd	0.036 hij	Sweet, spicy, banana
6‐Methyl‐5‐hepten‐2‐one	0.0024 ef	0.23 b	nd	nd	0.0089 def	0.059 cd	0.87 a	0.027 cdef	0.017 cdef	nd	Musty, banana, fruity
**Aromatic compounds**											
2‐Ethylfuran	0.51 efg	0.44 efgh	0.12 fgh	0.11 fgh	0.033 h	0.42 efgh	0.44 efgh	0.52 ef	0.13 fgh	1.3 d	Malty, cocoa, nutty
o‐Xylene	2.8 e	0.035 e	nd	nd	nd	12.0 bcd	18.0 b	1.3 e	nd	3.2 de	Geranium
Styrene	0.025 i	0.11 hi	nd	nd	nd	0.48 de	1.8 a	nd	nd	0.0079 i	Sweet, plastic, floral
Geosmin	nd	nd	nd	nd	nd	nd	nd	nd	nd	nd	Musty, earthy, fresh
**Terpenoids**											
L‐Limonene	0.018 de	0.032 de	nd	nd	nd	0.013 e	0.36 a	nd	nd	0.022 de	Camphoreous, herbal, terpenic
**Alkane**											
Decane	0.00094 gh	nd	nd	nd	nd	nd	0.0093 cd	0.0014 fgh	nd	nd	Unknown
**Sulfur compounds**											
Dimethyl disulfide	0.15 e	0.011 e	nd	0.0065 e	0.03 e	nd	0.44 e	5.8 bcd	2.4 de	0.59 e	Vegetable, onion, cabbage
Methional	nd	nd	nd	nd	nd	nd	nd	nd	nd	nd	Cabbage, pungent
**Nitrogen compounds**											
2,5‐Dimethyl pyrazine	0.0 e	0.14 b	nd	nd	nd	nd	0.032 cde	nd	0.0079 e	nd	Nutty, peanut, musty

These samples were analyzed in April 2024. Values represent the average of triplicate measurements grouped by chemical class. Pulses are organized by seed coat color, and treatments are grouped into flours, porridges, and boiled pulses to facilitate comparisons across related samples. nd, not detected. Odor profiles reflect the top three odor notes as reported by The Good Scents Company (2025).

Additionally, roasting significantly increased total ketones in RF samples of all pulse cultivars with the exception of CHKP, CR from the 2023 harvest and CHKP, MY from the 2022 harvest compared to their NRF counterparts (Figures 3C and 4C). The identified ketones included 2‐butanone, 2‐heptanone, and 6‐methyl‐5‐hepten‐2‐one. 2‐Butanone (camphoreous, acetone, fruity, ethereal) odor was the most abundant ketone in RF samples across all cultivars except MY, CHKP (Table 3). Additionally, 2‐heptanone, known for its fruity, spicy, cinnamon, green, and banana‐like odor (Burdock 2016) also increased after roasting in the RF samples of all cultivars compared to NRF (Table 3).

Furthermore, roasting also led to a slight but significant increase in total alcohol content across all cultivars from the 2022 harvest except GN and in MN, N, and WK from 2023 harvest (Figures 3A and 4A). The alcohols identified in the pulse samples were 1‐pentanol, 1‐hexanol, 1‐octen‐3‐ol, 1‐butanol, and 3‐methylbutanol. Among these, roasting increased the concentration of 1‐butanol and 3‐methyl butanol in RF samples compared to NRF across all cultivars, with the highest concentration of 1‐butanol in Otebo and 3‐methyl butanol in Great Northern beans (Tables 3 and S1).

Although, in our study, no significant differences in aldehyde concentration were found between NRF and RF samples for any of the pulse cultivars from both harvest years (Figures 3B and 4B), previously, Ma Zhen et al. (2016) reported higher aldehyde concentrations in roasted navy and red kidney bean flours and Lee et al. (2023) reported elevated levels of hexanal and benzaldehyde in roasted soybeans compared to their raw counterparts.

Our results align with Akkad et al. (2023), who reported a higher relative abundance of pyrazines and ketones in heat‐treated faba bean flour crackers than in untreated flour. Similarly, Ma Zhen et al. (2016) found higher alcohol concentrations in roasted green lentils and yellow peas flour than in untreated flour. These findings indicate that roasting fundamentally alters the volatile profile of pulses by inducing several chemical reactions between sugars, proteins, and minerals, alongside the breakdown of hydroxyl amino acids and the degradation of pigments. As a result, roasting leads to the formation of various volatile compounds, including sulfur compounds, pyrazines, pyridines, pyrroles, oxazoles, aldehydes, ketones, phenols, and carbon dioxide (Bhattacharya 2014). Ketones in legumes primarily form through the oxidation of saturated fatty acids at high temperatures and decarboxylation of 3‐oxo‐acids (Grebenteuch et al. 2021). Lee et al. (2023) further reported that 2‐heptanone, absent in untreated soybeans, appears after roasting. This suggests lipid oxidation (Oomah et al. 2014), and Maillard reactions contribute to its formation. Additionally, alcohol dehydrogenase (ADH) activity can convert LOX pathway products, transforming aldehydes or ketones into alcohols (Fischer et al. 2022).

The Maillard reaction, a nonenzymatic browning process driven by amino acids and reducing sugars, is a key driver of pyrazine formation during heat treatment (Yu et al. 2021). A GC‐O study by Bi et al. (2020) found that roasted pea flour contained high levels of pyrazines such as 2‐ethyl‐3,5‐dimethylpyrazine and 2,6‐dimethylpyrazine, which contributed to nutty and caramel‐like aromas. In contrast, raw pea flour was dominated by 3‐methylbutanoic acid and hexanal, which impart fatty, green, and grassy notes. Similarly, Kato et al. (1981) observed that D‐methyl‐ and 2‐ethyl‐5‐methyl‐pyrazines increased in roasted soybeans, masking beany flavors produced from aldehydes and alcohols. This suggests that roasting alters the volatile profile of pulses by generating pyrazines with roasted and nutty aromas that may help mask the grassy, green, and beany notes produced from alcohols and aldehydes.

Overall, roasting significantly increased ketone and alcohol concentrations due to lipid oxidation while also driving pyrazine formation through Maillard reactions in RFs.

##### 3.1.1.2 Roasted Model Product

In the RPs made from RF in both harvest years, alcohols and aldehydes were significantly lower while, sulfur concentrations were significantly higher in white‐colored (GN, N, O, WK) beans compared to NRP (Figures 3 and  4). Additionally, the total volatile concentration of targeted compounds reduced significantly by 70%–80% in the RP samples of white‐colored beans (GN, N, O, WK) compared to their NRP counterparts (Figure 2). Roasting and subsequent cooking into the model product significantly reduced the total alcohol concentration by an average of 57% in RP samples of white‐colored beans (GN, N, O, WK) and by 62% in yellow‐colored beans (MN, MY) compared to their NRP counterparts (Figure 3A). The high standard deviations observed in total volatile concentrations of MN could be attributed to instrumental variation between different days of GC–MS runs and potential inconsistencies in porridge preparation. Aliphatic alcohols such as 1‐hexanol, 1‐octen‐3‐ol, and 1‐pentanol were markedly reduced, while 1‐butanol and 3‐methyl butanol were almost absent in RP samples from the 2022 harvest (Table 3). Several of these alcohols have been identified as key contributors to beany flavors: 3‐methyl‐1‐butanol, which imparts an alcohol‐like odor (Gao et al. 2020); 1‐pentanol and 1‐octen‐3‐ol, associated with grassy, beany, and mushroom‐like odors (Xu et al. 2019). Additionally, 1‐hexanol contributes grassy, green, or leafy odors (Bott and Chambers IV 2006; Vara‐Ubol et al. 2004; Xu et al. 2019). Thus, reducing alcohol content through roasting may help mitigate common off‐flavors in pulses.

The aldehydes detected in pulse samples included 2‐methyl butanal, hexanal, (E)‐2‐hexenal, heptanal, benzaldehyde, octanal, nonanal, and decanal. Among these, (E)‐2‐hexenal, which contributes to grassy, green, and herbal flavors characteristic of the beany flavor in pulses, was the most abundant volatile and exhibited the highest concentration in the NRP samples of GN, N, and O cultivars (Oomah et al. 2014; Park et al. 2011; Sharan et al. 2022; Wang et al. 2020; Table 3). Roasting specifically decreased the concentration of hexanal (grassy, green, and herbal aroma), (E)‐2‐hexenal (green, grassy aroma), benzaldehyde (roasted, hazelnut, and almond odors), 2‐methyl butanal (pungent, fresh, fruity aroma), decanal (bitter gourd), heptanal (fatty, herbal, green odor) and nonanal (fatty, citrus, green aroma) in RP samples compared to their NRP counterparts (Table 3; Burdock 2016; The Good Scents Company 2025; Viana and English 2021). Aldehydes have been previously reported to produce green off‐flavors in peas and beany off‐flavors in soybeans (Roland et al. 2017; Sessa and Rackis 1977). Akkad et al. (2021) identified aldehydes (nonanal, octanal, hexanal, decanal, and 3‐methyl butanal) as key contributors to beany flavors in faba bean flours. Hexanal has also been identified as a source of off‐flavors in peas; the more hexanal was present, the less the peas were liked (Bengtsson and Bosund 1966). Thus, roasting may be a valuable pretreatment method as it significantly reduced (*p* < 0.05) total aldehyde concentrations by an average of 83% in white‐colored (GN, N, O, and WK) beans, particularly for those volatile compounds typically perceived as off‐flavors (Figure 3B). Roasting likely reduced these compounds by inactivating key enzymes involved in lipid oxidation pathways. According to Fischer et al. (2022), enzymatic lipid oxidation in legumes begins with LOX, which catalyzes the formation of fatty acid hydroperoxides. These are then cleaved by hydroperoxide lyase (HPL) to produce aldehydes—compounds strongly associated with beany off‐flavors. ADH and aldehyde dehydrogenase (ALDH) can further convert aldehydes and ketones into less odor‐active compounds such as alcohol and carboxylic acids, which generally have higher odor thresholds. For example, hexanal, known for its strong green odor and low detection threshold (4.5 ppb), is a more potent odorant than its reduced forms, hexanol (0.09–5.2 ppm) or hexanoic acid (3 ppm). Heat treatments like roasting can inactivate the LOX–HPL enzyme system, thereby reducing the formation of aldehydes and their alcohol derivatives and improving the flavor profile of pulse‐based ingredients.

On the other hand, roasting significantly increased (*p* < 0.05) the concentration of sulfurous compounds such as dimethyl disulfide in RP samples of white‐colored (GN, WK, N, O) and yellow‐colored (MN, MY) beans compared to their NRP counterparts (Figure 3E). Additionally, methional exhibited the highest concentration in RP samples of WK, N, and MY cultivars (Table 3). Our targeted GC–MS approach identified a 50‐fold increase in the total sulfur concentration due to roasting and cooking in RP compared to NRP (Table 3). Although, similar trends were observed from the 2023 harvest the results did not exhibit present significant changes (Figure 4E). Previous research has demonstrated that thermal processing leads to sulfur compound formation. Mishra et al. (2017) detected dimethyl sulfide, diethyl sulfide, methanethiol, dimethyl disulfide, and dimethyl sulfone in kidney beans exclusively after cooking (Chin and Lindsay 1994). Similarly, Bi et al. (2020) identified dimethyl sulfide, which imparts cabbage, sulfur, and sickly odors, as unique to roasted pea flour. These sulfurous compounds primarily arise from the degradation of methionine and cysteine amino acids during roasting and cooking. Methionine undergoes Strecker degradation during the final stages of the Maillard reaction, converting into methional, which has a low odor detection threshold (0.2 µg/L) and contributes to sulfurous and beany aromas in cooked kidney beans (Mishra et al. 2019). Further oxidation of methional produces methanethiol, which subsequently forms dimethyl disulfide and dimethyl trisulfide (Chin and Lindsay 1994). While total volatile concentration significantly decreased after roasting, sulfur‐containing compounds significantly increased in RP compared to NRP samples and due to their low odor detection threshold (< 1 µg/kg) they often contribute to sulfurous and objectionable odors especially at higher concentrations (McGorrin 2011).

Our results align with previous studies that have reported reductions in aldehydes, alcohols, and terpenes after cooking, alongside increases in sulfur‐containing compounds and pyrazines (Mishra et al. 2017). Similarly, Shariati‐Ievari (2013) demonstrated that burgers made with nonmicronized chickpea/lentil flours were characterized by higher concentrations of “beany” alcohols and aldehydes such as hexanol, 2‐hexenal, heptanal, hexanal, octanal, and nonanal compared to micronized flour at 130°C.

These shifts indicate that roasting alters the volatile composition and could consequently affect pulse flavor by increasing ketones (fruity), alcohols, and pyrazines (nutty, caramel) in flours, but the subsequent cooking of the flour further modifies these profiles by decreasing alcohol (green, mushroom‐like) and aldehyde (grassy, herbal) content but increasing sulfurous (cabbage, beany‐like) compounds, resulting in a net decrease in total volatile content for RF and RP samples (Mishra et al. 2019; The Good Scents Company 2025).

##### 3.1.1.3 Nonroasted Model Product

**FIGURE 5:**
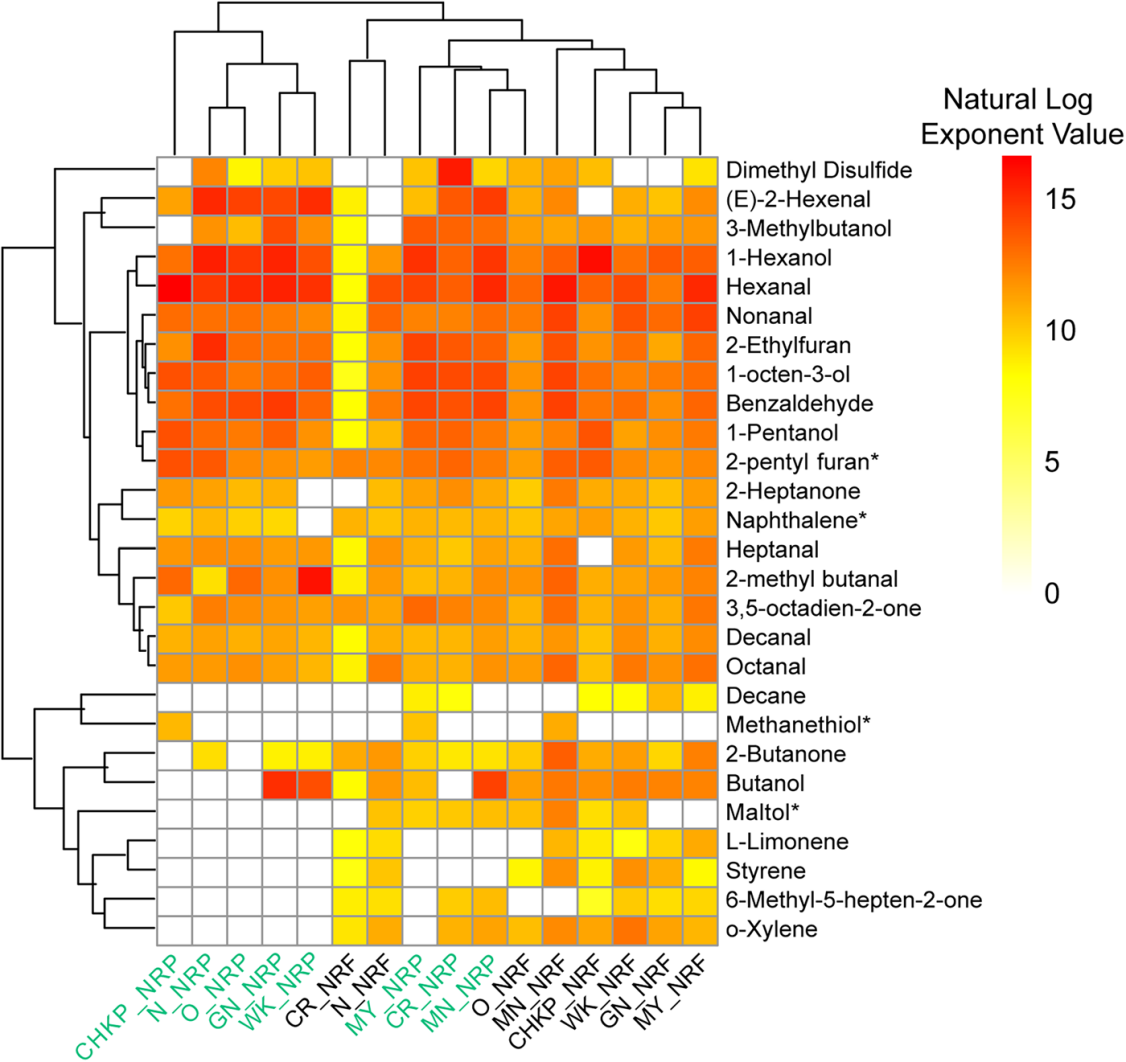
Heatmap of log‐scaled GC–MS peak areas for volatile compounds varying according to pulse variety from the following eight cultivars grown in 2022—Navy (N), Otebo (O), Cranberry (CR), Chickpea (CHKP), Manteca (MN), Mayacoba (MY), White Kidney (WK), and Great Northern (GN) for nonroasted flour (NRF) and nonroasted porridge (NRP) samples. The asterisks (*) refer to volatile compounds not authenticated using chemical standards. Pulse samples and volatile compounds are clustered according to hierarchical clustering analysis.

A heatmap plot (Figure 5) visualizes the variation between NRF and NRP samples on volatile peak areas (Table S2). The flour samples are clustered on the right half of the figure, while nonroasted model products are clustered on the left, illustrating the impact of the final cooking step before consumption on volatile composition of the model product. Volatile compounds were categorized into three distinct clusters based on their response to heat treatment. The top cluster included volatiles that increased after cooking, with higher concentrations in NRP compared to NRF. These compounds—hexanal, nonanal, 2‐pentyl furan, and 2‐heptanone—are known contributors to beany flavor (Akkad et al. 2023; Jiang et al. 2016). In contrast, the middle cluster consisted of long‐chain aldehydes and ketones that showed moderate variations, while the bottom cluster included aromatics and terpenoids that were more abundant in NRF but decreased or disappeared after cooking into NRP.

Cooking NRF into NRP led to the highest significant (*p* < 0.05) increase in total volatile concentration for white‐colored beans (GN, N, O, WK; Figure 2). Unlike the RP samples, cooking NRF into NRP increased the abundance of aldehydes in the top cluster of Figure 5 in white‐colored beans (GN, N, O, WK). Additionally, cooking NRF into NRP also increased alcohols like maltol, 1‐hexanol, and 3‐methyl butanol in all white‐colored (GN, N, O, WK) and yellow‐colored (MY, MN) beans. Cooking also increased the total aromatic concentration in NRP samples of N, CR, and MY compared to NRF samples (Table 3). These aromatics, such as furans, are primarily produced through the Maillard reaction and the thermal degradation of sugars, amino acids, carotenoids, and polyunsaturated fatty acids (PUFAs) like linoleic acid (Izzotti and Pulliero 2014; Min et al. 2003). On the other hand, cooking significantly reduced the concentration of the terpene limonene, which was notably absent in the NRP samples of white‐colored beans (N, O, WK, GN) but present in their NRF counterparts. Mishra et al. (2017) previously reported a significant reduction in terpenes of red kidney beans upon cooking. Similarly, Ma Zhen et al. (2016) observed a reduction in limonene content in navy and red kidney beans after cooking.

Overall cooking NRF into NRP influenced volatile formation pathways by increasing total volatiles, alcohols, and aldehydes while decreasing terpenoids in white‐colored beans.

#### 3.1.2 Boiling

Boiling significantly reduced total volatile concentrations, with BP samples exhibiting the lowest levels compared to NRP in both harvest years (Figure 2). This effect was particularly pronounced in white‐colored pulses (GN, N, O, WK), where boiling led to an average 95% decrease in total volatile concentration, while CHKP and CR showed a 75% reduction. Notably, boiling effectively reduced alcohol and aldehyde content, key contributors to beany flavors in pulses (Gao et al. 2020; Roland et al. 2017; Sessa and Rackis 1977; Xu et al. 2019). Alcohol concentrations dropped significantly (*p* < 0.05) in nearly all cultivars, averaging an 83% reduction (Figure 3B). Likewise, aldehyde concentrations declined by an average of 90% (Figure 3C).

Our findings align with previous research demonstrating significant reductions in volatile compounds during boiling. Ma Zhen et al. (2016) observed an average 61.75% reduction in targeted total volatile concentrations in boiled bean slurries of navy bean, red kidney bean, green lentil, and yellow pea compared to untreated flours. Azarnia et al. (2011) reported significantly reduced volatile concentrations in cooked peas and pea slurries, while Barra et al. (2007) found similar reductions in cooked French beans. Whitfield and Shipton (1966) also reported a decline in volatiles in blanched peas. Similarly, Del Rosario et al. (1984) found decreased alcohol concentrations in soybean and winged bean headspace samples upon heating, and Ma Zhen et al. (2016) reported reduced alcohol and aldehyde content in boiled bean slurries of navy beans, red kidney beans, green lentils, and yellow peas. These findings suggest that boiling and extended thermal treatments cause a loss or reduction of volatile compounds, particularly aldehydes and alcohols. The denaturation of proteins during wet heating exposes active sites in proteins, such as the α‐amino group of lysine and the thiol group of cysteine. These sites bind oxygenated lipid decomposition products, forming stable lipoprotein complexes that reduce the olfactory impact of volatile compounds (Beyeler and Solms 1974). As a result, the overall volatile concentration declines significantly in BP samples (Ma Zhen et al. 2016).

The impact of thermal processing on volatiles varies depending on the processing method (roasting vs. boiling), pulse type, and final product (flour vs. model product vs. boiled whole pulse). While boiling effectively reduced key beany flavor markers (aldehydes, alcohols) in pulses, roasting may offer a more practical pretreatment strategy because it (1) is easier to apply in the production of pulse flour, (2) preserves nutritional quality better than boiling, (3) is more energy‐efficient than other thermal treatments such as boiling and spray drying. For instance, Chukwuma et al. (2016) reported that roasting preserved the nutritional value of quality protein maize by retaining higher lysine and methionine content, while boiling led to greater nutrient loss. Roasted maize also retained significantly higher crude protein, crude fat, crude fiber, ash, and carbohydrate content compared to both boiled and raw maize. From an industrial perspective, manufacturers aim to improve product quality while reducing energy consumption. Okada et al. (1980) found that spray drying was the most energy‐intensive process, requiring 5040 kJ/kg IC, whereas roasting required only 890 kJ/kg IC.

Thus, while boiling significantly reduces key contributors to beany volatiles, roasting, on the other hand, offers a more energy‐efficient and scalable solution for processing pulse flours while preserving nutritional integrity.

### 3.2 Effect of Cultivar on Volatile Profiles

**FIGURE 6:**
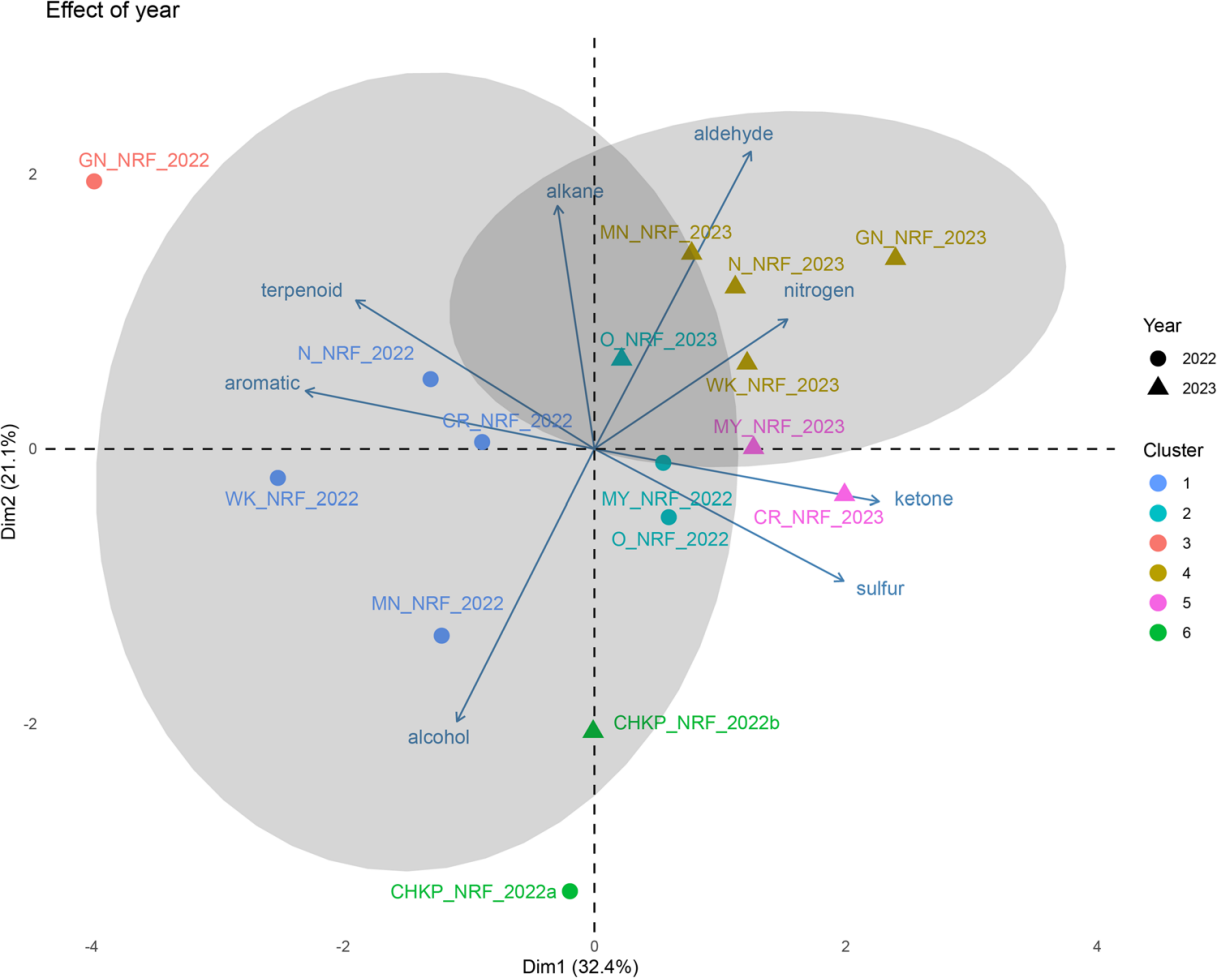
Principal component analysis biplot to visualize the effect of harvest year on concentrations of volatiles grouped by chemical classes in nonroasted flour (NRF) from pulse cultivars: Navy (N), Otebo (O), Cranberry (CR), Manteca (MN), Mayacoba (MY), White Kidney (WK), Great Northern (GN), grown in harvest years (2022 and 2023) from Michigan, and a market sample of Chickpea (CHKP; harvested in 2022). *CHKP_2022a: analyzed in April 2024 with 2022 samples; CHKP_2022b: analyzed in September 2024 with 2023 samples. Harvest samples from 2022 and 2022a are represented by circles (●) while, triangles (▲) denote samples from 2023 and 2022b harvest, respectively. Hierarchical cluster analysis grouped samples into clusters with similar volatile profiles, denoted by color.

Harvest year and seed coat color drove key differences in the volatile profiles of pulse cultivars. PCA revealed that PC1, PC2, and PC3 accounted for 32.4%, 21.1%, and 17.4% of the variance, respectively (Figure 6). HCA further highlighted distinct clustering patterns based on harvest year and pulse type. Cultivars from the 2022 harvest grouped into clusters 1, 2, and 3, while samples from the 2023 harvest showed distinct clustering based on seed coat color such that white‐ (GN, N, O, WK) and yellow‐ (MN, MY) colored beans formed cluster 4, whereas CR and CHKP grouped into clusters 5 and 6, respectively.

Yellow‐colored (MY, MN) beans, CR, and CHKP exhibited higher concentrations of sulfurous compounds compared to white‐colored cultivars (Figure 5). Among NRP samples, CR had the highest total sulfur concentration, particularly dimethyl disulfide. Similarly, CHKP and MY contained the highest levels of methanethiol (Figure 5). These findings align with Ma Zhen et al. (2016) who reported greater concentrations of dimethyl disulfide and methanethiol in untreated red kidney beans than in white‐colored navy beans. Future sensory studies should investigate whether the increased sulfurous concentration in darker colored and pigmented pulses influence their sensory perception compared to lighter colored varieties.

In contrast, white‐colored beans (GN, N, O, WK) contained higher concentrations of aldehydes, and alcohols. They exhibited elevated levels of alcohols like 1‐hexanol, maltol, 3‐methyl butanol and aldehydes such as hexanal, (E)‐2‐hexenal, benzaldehyde, and nonanal compared to CHKP and CR (Figure 5). (E)‐2‐Hexenal and 1‐hexanol were particularly abundant in NRP samples of white‐colored cultivars in GN, N, and O (Table 3). Consequently, among all cultivars, the NRP samples of white‐colored beans (GN, N, O, WK) had the highest total estimated volatile concentration, surpassing both yellow‐colored pulses (MY, MN), CHKP, and CR. Specifically, NRP samples of Navy beans exhibited the greatest volatile concentration, followed by Great Northern, Otebo, and White Kidney beans (Figure 2). Navy beans also had the highest concentration of compounds from the aromatic chemical class among all cultivars, with 2‐ethyl furan and 2‐pentyl furan dominating its NRP sample (Figure 5). These findings align with previous research by Ma Zhen et al. (2016) which reported that untreated navy bean flour contained the highest volatile abundance among Saskatchewan pulse varieties, whereas untreated red kidney bean flour had the lowest.

Previous research suggests that carotenoid degradation contributes to the formation of terpenoids and hydrocarbons (Murray et al. 1976; Wang and Arntfield 2017). Olumide et al. (n.d.) found that pigmented NRF samples of yellow‐colored beans (MY, MN) contained the highest carotenoid concentrations compared to white‐colored beans (GN, N, O, WK). Consequently, our study showed that NRF samples of yellow‐colored beans (MY, MN) exhibited the highest concentrations of terpenoids, particularly limonene, compared to white (GN, N, O, WK) and other (CHKP, CR) pulses (Figure 5). Previous research has also demonstrated that terpene content varies significantly by cultivar in common beans. Pinto beans, for instance, contain approximately 16 times more terpenes than black beans, while dark red kidney bean cultivars contain the lowest terpene content (Karolkowski et al. 2021; Oomah et al. 2007).

Overall, darker colored pulses were characterized by higher concentrations of sulfurous (cabbage, beany‐like) compounds and yellow‐colored beans contained the most terpenoids (citrus, orange) while, white‐colored beans were abundant in alcohols (green, mushroom‐like) and aldehydes (grassy, herbal). These “beany” flavors have been linked to reduced consumer liking and acceptability in pulse‐based products (English et al. 2019; Sanjeewa et al. 2010).

### 3.3 Effect of Year

The ANOVA results demonstrated that harvest year had a significant effect on total volatile concentrations across all samples (*p* < 0.05; Table 2). HCA and PCA further revealed distinct volatile profiles based on harvest year. Specifically, NRF samples from 2022 clustered in quadrants 2 and 3, while those from 2023 grouped in quadrants 1 and 4 (Figure 6). Within these clusters, samples from the 2022 harvest—N, WK, CR, and MN—grouped in cluster 1, while GN formed a distinct cluster 3 in quadrant 2. For the 2023 harvest, N, WK, GN, and MN clustered together (cluster 4) in quadrant 1, while MY and CR formed cluster 5. Notably, CHKP, harvested commercially in 2022 and analyzed at two time points, formed cluster 6 in quadrant 3, while the O cultivar from the 2022 and 2023 harvests grouped in cluster 2. The difference in clustering patterns across harvest years in beans may also be attributed to the varying time intervals between harvest and volatile analysis, as beans harvested in 2022 were analyzed 18 months postharvest, whereas those from 2023 were analyzed 12 months postharvest.

NRP and NRF from the 2022 harvest year exhibited higher total volatile concentrations compared to those from 2023 (Figure 2). This suggests seed maturity due to a prolonged storage period (18 months postharvest) in the mature 2022 harvest year samples could have influenced the accumulation of volatiles compared to the 2023 samples.

The NRF from the 2022 harvest showed higher concentrations of alcohols, ketones, and aromatics such as xylene and styrene across all cultivars (Tables 3 and S1), while NRF from the 2023 harvest exhibited higher concentrations of aldehydes than mature 2022 samples (Figure 6). This contrasts with previous studies by Manouel et al. (2024) where the concentration of hexanal in pea flours followed the order 2018 > 2019 > 2020 > 2022, indicating that increased seed age significantly increased hexanal content.

Interestingly, in our study, hexanal concentrations in NRF followed the order 2023 > 2022, except for CHKP (Tables 3 and S1). Since CHKP was commercially sourced, it was grown and harvested in a different location in 2022 compared to the other dry bean cultivars, although it was processed and analyzed within the same overall time frame. For instance, CHKP_2022a was analyzed after 18 months of storage in the same batch as the 2022 dry bean samples, and CHKP_2022b was analyzed after 30 months, alongside the 2023 dry bean samples. Despite this difference in storage time, both mature CHKP_2022b and newer CHKP_2022a NRF exhibited comparable volatile profiles and concentrations (Tables 3 and S1). This suggests that growing year and environmental conditions a pulse crop endures in a specific harvest year may have a greater influence on volatile profiles than a prolonged storage period alone.

Another possible factor is the temperature at which the pulses were stored. In our study, dry beans and pulses were stored at room temperature (22°C) until processing, and then pulse flours were stored under refrigeration (2°C) until analysis, which may have reduced changes in volatile profile during storage. The influence of storage time and temperature on volatile profiles was studied by Akkad et al. (2022), who observed that volatiles like hexanal and nonanal increased only with prolonged storage at room temperature (22°C) in faba bean flour while storage under refrigeration (4°C) or frozen (−21°C) conditions minimized volatile formation. However, our finding of lower hexanal content with increased seed age in the 2022 harvest NRF suggests that other factors related to harvest year, rather than storage duration, likely contributed to the differences observed.

Environmental conditions such as temperature, light exposure, water availability, and soil composition play a significant role in lipid metabolism, as plants under stress often produce more saturated fatty acids to stabilize cellular membranes. Additionally, genetic and biochemical responses specific to the growing environment may alter the activity of enzymes responsible for fatty acid synthesis, further impacting volatile profiles. Other differences in fatty acid composition due to location, harvest year, and storage duration could further influence the production of volatile compounds (Manouel et al. 2024).

These combined factors likely explain the differences in volatile profiles observed between the 2 years. In contrast to studies that showed an increase in aldehydes during storage, we observed lower aldehyde content in the more mature 2022 pulse samples, possibly due to early inactivation of LOX during roasting or milling and prolonged cold storage, as well as variations due to crop year. These insights are crucial for determining optimal storage time and temperature while considering crop year variations and the environmental and soil conditions during cultivation.

## 4 Conclusion

This study examined how cultivar, harvest year, and processing methods influenced the volatile composition of pulses. Cultivar differences were primarily driven by seed coat color, which played a key role in shaping volatile profiles. Understanding these differences can aid in selecting pulses for targeted food applications. The high relative stability of CHKP flours over time compared to dry bean samples suggests that environmental and growing conditions may have a greater influence on volatile profiles across harvest years than prolonged storage period alone. Processing methods altered VOC composition for both harvest years, with major differences observed between nonroasted and roasted samples. Cooking roasted flour was more effective in reducing key volatiles compared to cooking of nonroasted flours. Since these targeted volatiles have been cited as beany flavor markers, roasting may serve as an effective pretreatment strategy to reduce these flavors in cooked pulse‐based products. However, this decrease may also be attributed to the targeted GC–MS approach used in this study, which primarily quantified alcohols and aldehydes, leading to an overall reduction in the total estimated volatile concentration. Additionally, GC–MS analysis was conducted using sample weights based on a wet basis rather than standardized dry matter content across processing treatments. Specifically, 5 g of NRP, RP, and BP samples contained approximately 1 and 2 g of dry matter, respectively, compared to 2 g of dry matter in NRF and RF samples that were analyzed as dry powders with no added moisture. Despite the lower quantity of dry matter, NRP samples exhibited highest total volatile concentration compared to NRF, while, BP samples showed the lowest volatile concentration. These findings suggest that the observed changes in volatile content across processing methods are likely driven by the effects of thermal treatment rather than differences in dry matter content, although future work should consider dry matter standardization to enhance cross‐treatment comparisons.

Further research is needed to optimize roasting conditions based on seed size and color to minimize sulfur compound formation and to identify specific volatile markers associated with off‐flavors in pulses. Additionally, investigating the role of nitrogenous compounds generated during heat treatment is essential to identify if they mask or intensify off‐flavors in pulses.

Incorporating sensory analysis can give a better understanding of how volatile compounds influence odor perception and acceptability in pulse‐based products. Additionally, developing and validating instrumental methods for rapid profiling could identify volatile markers and predict sensory characteristics in pulse‐based products, ultimately reducing reliance on time‐intensive sensory panels.

## Author Contributions

**Kaveri Ponskhe**: writing – original draft, visualization, data curation, formal analysis, investigation, methodology, validation. **Aubrey DuBois**: formal analysis, writing – review and editing, methodology, visualization, project administration. **Randolph Beaudry**: methodology, supervision, resources, writing – review and editing. **Sharon Hooper**: writing – review and editing, methodology, supervision, investigation. **Karen Cichy**: resources, writing – review and editing, conceptualization, methodology, funding acquisition. **Emily J. Mayhew**: conceptualization, funding acquisition, writing – review and editing, methodology, supervision, visualization, formal analysis.

## Conflicts of Interest

The authors declare no conflicts of interest.

## Supporting information



Supporting Information: jfds70608‐sup‐0001‐SuppMat.docx

## Data Availability

Raw data will be made available upon request. After publication, raw data and data analysis scripts will be shared via a public GitHub repository.
