# Supplementary material for: Effect of Processing, Cultivar, and Crop Year on Volatile Composition in Pulses and Pulse Flours Analyzed by Headspace Solid‐Phase Microextraction Gas Chromatography–Mass Spectrometry
Source: J Food Sci. 2025 Oct 15;90(10):e70608. doi: 10.1111/1750-3841.70608 (PMC12522181; doi:10.1111/1750-3841.70608)
Supplement: Supplementary file 1 — Supporting Information: jfds70608‐sup‐0001‐SuppMat.docx [file JFDS-90-0-s001.docx]

**APPENDIX**

**Table S1:** Estimated concentration in nmol/L of volatiles quantified using authentic chemical standards across non-roasted flour (NRF), roasted flour (RF), non-roasted porridge (NRP), roasted porridge (RP), and boiled pulses (BP) from the 7 bean varieties (Great Northern, White Kidney, Navy, Otebo, Manteca and Mayacoba and Cranberry) grown in Michigan with a harvest year from 2023 and a market sample of Chickpea obtained commercially (harvested in 2022). These samples were analyzed August through September 2024. Values represent the average of triplicate measurements grouped by chemical class. Pulses are organized by seed coat color, and treatments are grouped into flours, porridges, and boiled pulses to facilitate comparisons across related samples. nd: not detected. Odor profiles reflect the top three odor notes as reported by (The Good Scents Company, 2025).

| **White colored beans (estimated volatile concentration in nmol/L)** | | | | | | | | | | | | |
| --- | --- | --- | --- | --- | --- | --- | --- | --- | --- | --- | --- | --- |
|  | **Great northern beans** | | | | | **White Kidney beans** | | | | |  | |
| **Compound Name** | **NRF** | **RF** | **NRP** | **RP** | **BP** | **NRF** | **RF** | **NRP** | **RP** | **BP** | **Odor profiles from (The Good Scents Company, 2025)** |  |
| **ALDEHYDE** | | | | | | | | | | | | |
| 2-Methyl butanal | 1.6 c | 2.8 c | 8.9 c | 1.1 c | nd | 3.2 c | 9.3 c | 98.0 a | 2.5 c | 0.24 c | malty, musty, fermented |  |
| Hexanal | 8.6 def | 7.6 defgh | 17.0 c | 1.3 lmno | 0.46 mno | 8.0 defg | 9.9 de | 11.0 d | 1.4 lmno | 0.27 no | vegetable, aldehydic, clean |  |
| (E)-2-hexenal | 0.21 c | 0.2 c | 5.5 bc | 0.32 c | nd | 0.19 c | 0.28 c | 17.0 a | 1.3 c | nd | sweet, vegetable, bitter almond |  |
| Heptanal | 0.24 ijkl | 0.32 fghij | 0.41 efgh | 0.048 n | 0.045 n | 0.37 fghi | 0.55 de | 0.32 fghij | 0.12 lmn | 0.046 n | aldehydic, fatty, herbal |  |
| Benzaldehyde | 0.2 hijkl | 0.3 ghijkl | 1.2 bc | 0.81 de | 0.06 l | 0.16 ijkl | 0.45 fgh | 1.7 a | 1.4 b | 0.15 ijkl | sweet, cherry, nutty |  |
| Octanal | 0.11 efghi | 0.057 hijklmno | 0.098 fghijk | 0.046 jklmno | 0.028 mno | 0.15 cdef | 0.24 b | 0.19 bc | 0.096 fghijkl | 0.049 ijklmno | aldehydic, fatty, herbal |  |
| Nonanal | 0.67 defghijk | 0.44 ijklmn | 0.47 ijklmn | 0.23 klmn | 0.11 mn | 0.67 defghijk | 1.1 cd | 1.1 cde | 0.62 fghijkl | 0.27 jklmn | aldehydic, fatty, rose |  |
| Decanal | 0.075 cdefgh | 0.049 efgh | 0.069 cdefgh | 0.057 defgh | 0.025 gh | 0.099 abcde | 0.13 abc | 0.15 ab | 0.13 abc | 0.027 gh | sweet, aldehydic, floral |  |
| **ALCOHOL** | | | | | | | | | | | | |
| Butanol | 0.43 bcd | 0.57 bcd | 0.16 cd | 0.1 d | nd | 0.46 bcd | 1.5 ab | 0.11 d | 0.093 d | nd | sweet, fermented, oily |  |
| 3-Methylbutanol | 1.1 e | 2.8 cde | 6.8 bcd | 16.0 a | 0.04 e | 0.075 e | 4.1 cde | 1.5 de | 6.7 bcd | nd | musty, vegetable, cocoa |  |
| 1-Pentanol | 0.33 fgh | 0.42 fgh | 2.0 d | 0.81 efgh | 0.011 h | 0.14 h | 0.28 gh | 1.1 efg | 0.34 fgh | 0.022 h | sweet, fermented, yeasty |  |
| 1-Hexanol | 0.47 hij | 0.18 j | 6.2 b | 2.7 ef | 0.011 j | 0.13 j | 0.15 j | 4.0 cd | 1.1 ghi | 0.012 j | sweet, pungent, herbal |  |
| 1-Octen-3-ol | 0.11 jklmnop | 0.14 ijklmn | 0.16 ijklm | 0.095 klmnopqr | 0.0066 r | 0.11 jklmnopq | 0.18 hijk | 0.54 b | 0.55 b | 0.018 pqr | vegetable, mushroom, chicken |  |
| **KETONE** | | | | | | | | | | | | |
| 2-Butanone | 11.0 cdef | 17.0 bc | 1.1 gh | 0.27 h | nd | 15.0 cd | 35.0 a | 1.0 h | 0.49 h | nd | camphoreous, acetone, fruity |  |
| 2-Heptanone | 0.04 ghijklm | 0.081 cde | 0.099 cd | 0.057 efghi | 0.0034 n | 0.032 hijklmn | 0.11 c | 0.072 def | 0.025 jklmn | 0.0054 n | sweet, spicy, banana |  |
| 6-Methyl-5-hepten-2-one | 0.007 fg | 0.059 cde | 0.017 efg | 0.00019 g | 0.0022 g | 0.023 efg | 0.13 b | 0.0000018 g | 0.046 def | 0.0057 g | musty, banana, fruity |  |
| **AROMATIC COMPOUNDS** | | | | | | | | | | | | |
| 2-Ethylfuran | 0.49 cde | 0.41 defgh | 0.49 cde | 0.052 kl | 0.12 ijkl | 0.45 cdef | 1.4 a | 0.35 efghij | 0.098 ijkl | 0.15 ghijkl | malty, cocoa, nutty |  |
| o-Xylene | 0.064 b | 0.089 a | 0.015 ghi | nd | 0.0057 i | 0.035 defg | 0.051 bcd | nd | nd | 0.016 ghi | geranium |  |
| Styrene | 0.024 bcd | 0.044 a | 0.0071 f | 0.0081 ef | nd | 0.026 bcd | 0.04 a | nd | nd | nd | sweet, plastic, floral |  |
| Geosmin | nd | nd | nd | nd | nd | nd | nd | nd | nd | nd | musty, earthy, fresh |  |
| **TERPENOIDS** | | | | | | | | | | | | |
| L-limonene | 0.016 bc | 0.0097 bc | 0.003 bc | 0.0017 c | nd | 0.012 bc | 0.18 b | 0.0024 c | 0.0039 bc | nd | camphoreous, herbal, terpenic |  |
| **ALKANES** | | | | | | | | | | | | |
| Decane | 0.022 bcde | 0.029 abc | 0.0081 fghi | 0.0071 ghi | 0.0055 hi | 0.02 cde | 0.026 bcd | 0.018 cdefg | 0.0057 hi | 0.0025 i | unknown |  |
| **SULFUR COMPOUNDS** | | | | | | | | | | | | |
| Dimethyl Disulfide | 0.067 c | 0.39 c | 0.065 c | 0.27 c | nd | nd | 0.37 c | 0.04 c | 1.1 bc | 0.032 c | vegetable, onion, cabbage |  |
| Methional | nd | 0.0018 ef | 0.00092 ef | 0.022 a | nd | nd | 0.0059 cdef | nd | 0.011 bc | 0.0016 ef | cabbage, pungent |  |
| **NITROGEN COMPOUNDS** | | | | | | | | | | | | |
| 2,5-Dimethyl pyrazine | 0.013 de | 0.019 cde | 0.00014 e | 0.036 bc | nd | nd | 0.061 a | nd | 0.026 cd | nd | nutty, peanut, musty |  |
| **White colored beans (estimated volatile concentration in nmol/L)** | | | | | | | | | | | | |
|  | **Navy bean** | | | | | **Otebo** | | | | |  | |
| **Compound Name** | **NRF** | **RF** | **NRP** | **RP** | **BP** | **NRF** | **RF** | **NRP** | **RP** | **BP** | **Odor profiles from (The Good Scents Company, 2025)** |  |
| **ALDEHYDE** | | | | | | | | | | | | |
| 2-Methyl butanal | 1.3 c | 3.8 c | 0.23 c | 0.26 c | 0.67 c | 1.8 c | 73.0 ab | 2.0 c | 0.42 c | 1.3 c | malty, musty, fermented |  |
| Hexanal | 4.1 hijklm | 3.7 ijklmno | 3.6 ijklmno | 1.3 lmno | 0.92 lmno | 5.7 fghij | 11.0 d | 2.9 ijklmno | 0.72 lmno | 4.1 hijklm | vegetable, aldehydic, clean |  |
| (E)-2-hexenal | 0.13 c | 0.18 c | 4.5 bc | 1.4 c | nd | 0.15 c | 2.6 bc | 0.98 c | nd | 0.13 c | sweet, vegetable, bitter almond |  |
| Heptanal | 0.22 ijklm | 0.46 defg | 0.054 mn | 0.044 n | 0.028 n | 0.46 defg | 0.3 hijk | 0.1 lmn | 0.037 n | 0.22 ijklm | aldehydic, fatty, herbal |  |
| Benzaldehyde | 0.11 kl | 0.31 fghijkl | 0.25 hijkl | 0.37 fghijk | 0.1 kl | 0.21 hijkl | 0.73 e | 0.81 de | 0.052 l | 0.11 kl | sweet, cherry, nutty |  |
| Octanal | 0.08 hijklm | 0.12 defgh | 0.036 lmno | 0.039 klmno | 0.019 no | 0.15 cdefg | 0.1 fghij | 0.066 hijklmno | 0.013 o | 0.08 hijklm | aldehydic, fatty, herbal |  |
| Nonanal | 0.76 cdefghi | 0.78 cdefghi | 0.21 klmn | 0.12 mn | 0.12 mn | 1.0 cdefg | 0.44 ijklmn | 0.43 ijklmn | 0.062 n | 0.76 cdefghi | aldehydic, fatty, rose |  |
| Decanal | 0.13 abc | 0.039 efgh | 0.02 gh | 0.019 h | 0.028 fgh | 0.13 abc | 0.044 efgh | 0.044 efgh | 0.017 h | 0.13 abc | sweet, aldehydic, floral |  |
| **ALCOHOL** | | | | | | | | | | | | |
| Butanol | 0.39 bcd | 0.33 bcd | nd | 0.2 bcd | nd | 0.31 bcd | 2.2 a | 0.36 bcd | 0.041 d | 0.39 bcd | sweet, fermented, oily |  |
| 3-Methylbutanol | 0.095 e | 1.5 de | 1.5 de | 3.4 cde | 0.019 e | 1.6 de | 0.59 e | 0.63 e | nd | 0.095 e | musty, vegetable, cocoa |  |
| 1-Pentanol | 0.083 h | 0.24 gh | 0.48 fgh | 0.32 fgh | 0.043 h | 0.26 gh | 0.76 efgh | 0.36 fgh | nd | 0.083 h | sweet, fermented, yeasty |  |
| 1-Hexanol | 0.073 j | 0.066 j | 1.1 ghi | 0.58 hij | 0.023 j | 0.16 j | 2.9 e | 1.1 ghi | 0.025 j | 0.073 j | sweet, pungent, herbal |  |
| 1-Octen-3-ol | 0.076 lmnopqr | 0.17 ijkl | 0.2 ghij | 0.22 fghi | 0.011 qr | 0.12 jklmno | 0.27 efgh | 0.13 ijklmn | 0.0066 r | 0.076 lmnopqr | vegetable, mushroom, chicken |  |
| **KETONE** | | | | | | | | | | | | |
| 2-Butanone | 8.5 defgh | 37.0 a | 9.5 cdefg | 2.3 fgh | 0.19 h | 12.0 cde | 0.45 h | 1.0 gh | nd | 8.5 defgh | camphoreous, acetone, fruity |  |
| 2-Heptanone | 0.015 klmn | 0.041 ghijkl | 0.01 mn | 0.029 ijklmn | 0.0054 n | 0.062 efg | 0.041 ghijkl | 0.033 ghijklmn | 0.0048 n | 0.015 klmn | sweet, spicy, banana |  |
| 6-Methyl-5-hepten-2-one | 0.021 efg | 0.093 bcd | 0.013 fg | 0.017 efg | 0.001 g | 0.012 fg | 0.038 efg | 0.021 efg | 0.0027 g | 0.021 efg | musty, banana, fruity |  |
| **AROMATIC COMPOUNDS** | | | | | | | | | | | | |
| 2-Ethylfuran | 0.12 ijkl | 0.64 cd | 0.36 efghi | 0.42 defg | 0.016 l | 0.45 cdef | 0.23 efghijkl | 0.15 ghijkl | 0.012 l | 0.12 ijkl | malty, cocoa, nutty |  |
| o-Xylene | nd | 0.023 efghi | nd | nd | nd | 0.05 bcde | 0.013 hi | nd | 0.034 defgh | nd | geranium |  |
| Styrene | nd | nd | nd | nd | nd | 0.024 bcd | nd | nd | 0.0036 f | nd | sweet, plastic, floral |  |
| Geosmin | nd | nd | nd | nd | nd | nd | nd | nd | nd | nd | musty, earthy, fresh |  |
| **TERPENOIDS** | | | | | | | | | | | | |
| L-limonene | 0.0062 bc | 0.02 bc | nd | nd | nd | 0.4 a | 0.0022 c | 0.0028 bc | nd | 0.0062 bc | camphoreous, herbal, terpenic |  |
| **ALKANES** | | | | | | | | | | | | |
| Decane | 0.013 efghi | 0.016 defgh | nd | nd | 0.0035 i | 0.025 bcd | 0.0038 hi | 0.0064 hi | 0.006 hi | 0.013 efghi | unknown |  |
| **SULFUR COMPOUNDS** | | | | | | | | | | | | |
| Dimethyl Disulfide | nd | 0.098 c | 0.02 c | 3.9 a | 0.024 c | 0.28 c | 0.051 c | 3.5 ab | 0.04 c | nd | vegetable, onion, cabbage |  |
| Methional | nd | 0.0012 ef | nd | nd | 0.0011 ef | 0.0029 def | 0.0008 ef | 0.0086 bcd | 0.00065 f | nd | cabbage, pungent |  |
| **NITROGEN COMPOUNDS** | | | | | | | | | | | | |
| 2,5-Dimethyl pyrazine | nd | 0.026 cd | nd | nd | nd | 0.017 cde | 0.015 cde | 0.014 de | nd | nd | nutty, peanut, musty |  |
| **Yellow colored beans (estimated volatile concentration in nmol/L)** | | | | | | | | | | | | |
|  | **Manteca beans** | | | | | **Mayacoba beans** | | | | |  | |
| **Compound Name** | **NRF** | **RF** | **NRP** | **RP** | **BP** | **NRF** | **RF** | **NRP** | **RP** | **BP** | **Odor profiles from (The Good Scents Company, 2025)** |  |
| **ALDEHYDE** | | | | | | | | | | | | |
| 2-Methyl butanal | 1.8 c | 5.7 c | 16.0 bc | 6.2 c | 0.4 c | 1.0 c | 2.7 c | 45.0 bc | 2.7 c | 0.13 c | malty, musty, fermented |  |
| Hexanal | 4.5 ghijkl | 11.0 d | 17.0 c | 2.6 ijklmno | 0.67 mno | 4.0 hijklmn | 5.8 fghij | 5.3 fghijk | 0.59 mno | 0.29 no | vegetable, aldehydic, clean |  |
| (E)-2-hexenal | 0.26 c | 0.4 c | 18.0 a | 4.1 bc | 0.17 c | 0.18 c | 0.2 c | 7.4 b | 1.2 c | 0.082 c | sweet, vegetable, bitter almond |  |
| Heptanal | 0.22 ijklm | 0.6 cd | 0.71 bc | 0.23 ijklm | 0.05 n | 0.13 lmn | 0.33 fghij | 0.13 lmn | 0.076 lmn | 0.027 n | aldehydic, fatty, herbal |  |
| Benzaldehyde | 0.13 jkl | 0.36 fghijk | 1.0 cd | 1.2 bc | 0.42 fghi | 0.23 hijkl | 0.41 fghij | 0.58 efg | 0.74 e | 0.24 hijkl | sweet, cherry, nutty |  |
| Octanal | 0.085 hijklm | 0.065 hijklmno | 0.16 cde | 0.076 hijklmn | 0.035 mno | 0.057 hijklmno | 0.17 cd | 0.065 hijklmno | 0.075 hijklmn | 0.016 no | aldehydic, fatty, herbal |  |
| Nonanal | 0.95 cdefgh | 0.66 defghijk | 0.7 defghij | 0.49 hijklmn | 0.27 jklmn | 0.37 ijklmn | 1.0 cdef | 0.35 ijklmn | 0.54 ghijklm | 0.1 mn | aldehydic, fatty, rose |  |
| Decanal | 0.12 abcd | 0.043 efgh | 0.11 abcd | 0.051 defgh | 0.034 fgh | 0.021 gh | 0.052 defgh | 0.058 defgh | 0.1 abcde | 0.028 fgh | sweet, aldehydic, floral |  |
| **ALCOHOL** | | | | | | | | | | | | |
| Butanol | 0.12 d | 0.81 bcd | 0.072 d | 0.062 d | nd | 0.27 bcd | 0.58 bcd | 0.0027 d | 0.18 bcd | nd | sweet, fermented, oily |  |
| 3-Methylbutanol | 0.13 e | 2.9 cde | 3.0 cde | 4.8 cde | nd | 0.39 e | 1.6 de | 0.42 e | 2.2 de | nd | musty, vegetable, cocoa |  |
| 1-Pentanol | 0.11 h | 0.52 fgh | 1.1 ef | 0.42 fgh | nd | 0.084 h | 0.19 gh | 0.2 gh | 0.19 gh | nd | sweet, fermented, yeasty |  |
| 1-Hexanol | 0.26 ij | 0.17 j | 3.6 de | 1.9 fg | 0.041 j | 0.1 j | 0.065 j | 1.2 gh | 0.71 hij | 0.016 j | sweet, pungent, herbal |  |
| 1-Octen-3-ol | 0.063 nopqr | 0.19 ghijk | 0.35 cde | 0.29 defg | 0.029 opqr | 0.079 lmnopqr | 0.14 ijklmn | 0.37 cd | 0.3 def | 0.024 opqr | vegetable, mushroom, chicken |  |
| **KETONE** | | | | | | | | | | | | |
| 2-Butanone | 8.6 defgh | 25.0 b | 0.8 h | 5.0 efgh | 0.24 h | 14.0 cd | 41.0 a | 0.42 h | 0.79 h | nd | camphoreous, acetone, fruity |  |
| 2-Heptanone | 0.023 jklmn | 0.076 de | 0.045 fghij | 0.028 ijklmn | 0.0074 n | 0.016 klmn | 0.042 ghijk | 0.024 jklmn | 0.021 jklmn | 0.0056 n | sweet, spicy, banana |  |
| 6-Methyl-5-hepten-2-one | 0.031 efg | 0.039 defg | 0.031 efg | 0.027 efg | 0.02 efg | 0.013 efg | 0.4 a | 0.1 bc | 0.033 efg | 0.0069 fg | musty, banana, fruity |  |
| **AROMATIC COMPOUNDS** | | | | | | | | | | | | |
| 2-Ethylfuran | 0.29 efghijkl | 1.4 a | 1.0 b | 0.27 efghijkl | 0.071 jkl | 0.27 efghijkl | 0.32 efghijk | 0.27 efghijkl | 0.057 kl | 0.04 l | malty, cocoa, nutty |  |
| o-Xylene | 0.042 cdef | 0.045 cde | nd | nd | 0.0098 hi | nd | 0.022 fghi | 0.018 ghi | nd | 0.0095 i | geranium |  |
| Styrene | 0.033 ab | 0.028 bc | nd | nd | nd | 0.015 cdef | 0.019 cde | 0.006 f | 0.0061 f | nd | sweet, plastic, floral |  |
| Geosmin | nd | nd | nd | nd | nd | nd | nd | nd | nd | nd | musty, earthy, fresh |  |
| **TERPENOIDS** | | | | | | | | | | | | |
| L-limonene | 0.021 bc | 0.012 bc | 0.0015 c | 0.0012 c | 0.0022 c | 0.01 bc | 0.11 bc | 0.0068 bc | 0.0024 c | nd | camphoreous, herbal, terpenic |  |
| **ALKANES** | | | | | | | | | | | | |
| Decane | 0.026 bcd | 0.02 cdef | 0.0072 ghi | 0.0036 hi | 0.0035 i | 0.02 cde | 0.026 bcd | 0.0072 ghi | 0.0057 hi | 0.0066 hi | unknown |  |
| **SULFUR COMPOUNDS** | | | | | | | | | | | | |
| Dimethyl Disulfide | nd | 0.43 c | 0.15 c | 1.8 abc | 0.37 c | nd | 0.11 c | nd | 4.2 a | 0.15 c | vegetable, onion, cabbage |  |
| Methional | nd | 0.0036 def | 0.0013 ef | 0.0075 bcde | 0.0054 cdef | nd | nd | nd | 0.0072 bcde | 0.002 ef | cabbage, pungent |  |
| **NITROGEN COMPOUNDS** | | | | | | | | | | | | |
| 2,5-Dimethyl pyrazine | nd | 0.014 de | nd | 0.014 de | 0.0041 e | nd | 0.051 ab | nd | 0.034 bc | nd | nutty, peanut, musty |  |
| **Other pulses (estimated volatile concentration in nmol/L)** | | | | | | | | | | | | |
|  | **Chickpea 2022^b^** | | | | | **Cranberry beans** | | | | |  | |
| **Compound Name** | **NRF** | **RF** | **NRP** | **RP** | **BP** | **NRF** | **RF** | **NRP** | **RP** | **BP** | **Odor profiles from (The Good Scents Company, 2025)** |  |
| **ALDEHYDE** | | | | | | | | | | | | |
| 2-Methyl butanal | 0.85 c | 1.3 c | 0.019 c | 11.0 c | 0.7 c | 2.1 c | 2.2 c | 0.3 c | 1.3 c | 0.19 c | malty, musty, fermented |  |
| Hexanal | 0.25 o | 0.53 mno | 55.0 b | 64.0 a | 1.7 klmno | 7.9 defg | 5.2 fghijk | 6.3 efghi | 2.3 jklmno | 0.49 mno | vegetable, aldehydic, clean |  |
| (E)-2-hexenal | 0.065 c | nd | 0.67 c | 0.73 c | nd | 0.13 c | 0.17 c | 7.8 b | 0.87 c | nd | sweet, vegetable, bitter almond |  |
| Heptanal | 0.47 def | 0.048 n | 0.76 b | 1.4 a | 0.11 lmn | 0.16 jklmn | 0.31 ghijk | 0.18 jklmn | 0.15 klmn | 0.049 n | aldehydic, fatty, herbal |  |
| Benzaldehyde | 0.13 jkl | 0.17 hijkl | 0.43 fghi | 0.59 ef | 0.22 hijkl | 0.17 hijkl | 0.24 hijkl | 0.82 de | 0.81 de | 0.12 kl | sweet, cherry, nutty |  |
| Octanal | 0.013 o | 0.037 lmno | 0.15 cdef | 0.4 a | 0.088 ghijklm | 0.033 mno | 0.062 hijklmno | 0.1 fghij | 0.087 ghijklm | 0.037 lmno | aldehydic, fatty, herbal |  |
| Nonanal | 0.11 mn | 0.21 klmn | 1.2 c | 2.6 a | 1.0 cdefg | 0.25 jklmn | 0.51 hijklmn | 0.49 hijklmn | 0.62 efghijkl | 0.19 lmn | aldehydic, fatty, rose |  |
| Decanal | 0.025 gh | 0.07 cdefgh | 0.092 abcdef | 0.15 a | 0.034 fgh | nd | 0.057 defgh | 0.13 abc | 0.084 bcdefg | 0.026 gh | sweet, aldehydic, floral |  |
| **ALCOHOL** | | | | | | | | | | | | |
| Butanol | 0.51 bcd | 1.4 abc | 0.045 d | 0.24 bcd | nd | 0.52 bcd | 0.44 bcd | 0.075 d | 0.12 d | nd | sweet, fermented, oily |  |
| 3-Methylbutanol | 0.38 e | 0.98 e | 11.0 ab | 13.0 ab | 0.23 e | 0.56 e | 0.8 e | 5.2 cde | 7.8 bc | nd | musty, vegetable, cocoa |  |
| 1-Pentanol | 1.6 de | 2.9 c | 5.8 a | 4.6 b | 0.037 h | 0.2 gh | 0.23 gh | 0.46 fgh | 0.72 efgh | nd | sweet, fermented, yeasty |  |
| 1-Hexanol | 4.2 cd | 7.8 a | 3.8 cd | 4.7 c | 0.048 j | 0.05 j | 0.12 j | 1.8 g | 1.1 ghi | 0.012 j | sweet, pungent, herbal |  |
| 1-Octen-3-ol | 0.13 ijklmn | 0.024 opqr | 0.63 b | 1.0 a | 0.022 opqr | 0.075 lmnopqr | 0.099 klmnopqr | 0.42 c | 0.36 cde | 0.014 pqr | vegetable, mushroom, chicken |  |
| **KETONE** | | | | | | | | | | | | |
| 2-Butanone | 8.6 defgh | 8.5 defgh | 0.17 h | 0.55 h | nd | 24.0 b | 14.0 cd | 0.9 h | 0.8 h | 0.27 h | camphoreous, acetone, fruity |  |
| 2-Heptanone | nd | 0.058 efgh | 0.26 b | 0.38 a | 0.013 lmn | 0.014 klmn | 0.042 ghijk | 0.031 hijklmn | 0.021 jklmn | 0.0061 n | sweet, spicy, banana |  |
| 6-Methyl-5-hepten-2-one | 0.01 fg | 0.031 efg | 0.022 efg | 0.023 efg | 0.0033 g | 0.011 fg | nd | 0.041 defg | 0.036 efg | 0.0076 fg | musty, banana, fruity |  |
| **AROMATIC COMPOUNDS** | | | | | | | | | | | | |
| 2-Ethylfuran | 0.14 hijkl | 0.25 efghijkl | 0.15 ghijkl | 0.69 cd | 0.073 jkl | 0.7 c | 0.31 efghijkl | 0.35 efghij | 0.17 ghijkl | 0.11 ijkl | malty, cocoa, nutty |  |
| o-Xylene | nd | 0.047 bcde | 0.019 ghi | 0.021 fghi | nd | nd | 0.042 cdef | nd | nd | nd | geranium |  |
| Styrene | nd | 0.02 cde | 0.024 bcd | 0.017 cdef | nd | nd | 0.014 def | nd | nd | nd | sweet, plastic, floral |  |
| Geosmin | nd | nd | nd | nd | nd | nd | nd | nd | nd | nd | musty, earthy, fresh |  |
| **TERPENOIDS** | | | | | | | | | | | | |
| L-limonene | 0.011 bc | 0.031 bc | 0.0049 bc | 0.0068 bc | nd | 0.0057 bc | 0.013 bc | 0.0029 bc | 0.0031 bc | nd | camphoreous, herbal, terpenic |  |
| **ALKANES** | | | | | | | | | | | | |
| Decane | 0.011 efghi | 0.026 bcd | 0.017 cdefgh | 0.038 ab | 0.0036 hi | 0.04 a | 0.026 bcd | 0.004 hi | 0.005 hi | 0.0038 hi | unknown |  |
| **SULFUR COMPOUNDS** | | | | | | | | | | | | |
| Dimethyl Disulfide | 0.029 c | 0.019 c | nd | 0.031 c | 0.015 c | 0.12 c | 0.05 c | 0.017 c | 3.5 ab | 0.037 c | vegetable, onion, cabbage |  |
| Methional | nd | nd | nd | nd | 0.00029 f | nd | 0.0022 def | 0.0053 cdef | 0.013 b | 0.00065 f | cabbage, pungent |  |
| **NITROGEN COMPOUNDS** | | | | | | | | | | | | |
| 2,5-Dimethyl pyrazine | nd | nd | 0.0071 de | 0.0084 de | nd | nd | 0.0081 de | nd | 0.0062 e | nd | nutty, peanut, musty |  |

**Table S2:** Average peak areas of volatiles quantified using means of triplicate measurements from area under the curve and reported for a single m/z (mass-to-charge ratio) using the respective unique mass of volatiles grouped by chemical class across non-roasted flour (NRF), roasted flour (RF), non-roasted porridge (NRP), roasted porridge (RP), and boiled pulses (BP) from a market sample of Chickpea obtained commercially (harvested in 2022) and seven bean varieties- Cranberry, Great Northern, Manteca and Mayacoba, Navy, Otebo and White Kidney grown in Michigan from harvest years 2022 and 2023 respectively. *2022^a^: analyzed in April 2024; *2022^b^: analyzed in September 2024. Volatiles annotated as MS, NIST: compared mass spectrum with National Institute of Standards and Technology (NIST) mass spectra library database (V.05); RT, STD: compared retention time and spectrum of identified compound with those of an authentic compound. by comparisons with the National Institute of Standards and Technology (NIST) mass spectra library database (V.05) and/or by matching retention times of authenticated standards, nd: not detected. Odor profiles reflect the top three odor notes as reported by (The Good Scents Company, 2025).

| **Chickpea cv. ‘Sierra’ (2022^a^ )** | | | | | | | | |
| --- | --- | --- | --- | --- | --- | --- | --- | --- |
|  | **Average Area Counts** | | | | |  |  |  |
| **Compound Name** | **NRF** | **RF** | **NRP** | **RP** | **BP** | **Odor profiles from (The Good Scents Company, 2025)** | **Unique Mass (m/z)** | **Annotation** |
| **ALDEHYDE** | | | | | | | | |
| 2-Methyl butanal | 57980 | 414889 | 546041 | nd | 34004 | malty, musty, fermented | 57 | MS,NIST,RT,STD |
| Hexanal | 687411 | 1428640 | 17152259 | 16002248 | 891104 | vegetable, aldehydic, clean | 57 | MS,NIST,RT,STD |
| (E)-2-hexenal | nd | nd | 88064 | 77540 | nd | sweet, vegetable, bitter almond | 55 | MS,NIST,RT,STD |
| Heptanal | nd | 132085 | 126462 | 132464 | 62000 | aldehydic, fatty, herbal | 70 | MS,NIST,RT,STD |
| Benzaldehyde | 365744 | 1926040 | 396589 | 349750 | 852029 | sweet, cherry, nutty | 77 | MS,NIST,RT,STD |
| Octanal | 32511 | 256267 | 102541 | 102915 | 144297 | aldehydic, fatty, herbal | 44 | MS,NIST,RT,STD |
| Nonanal | 140263 | 1143847 | 485943 | 501537 | 405945 | aldehydic, fatty, rose | 57 | MS,NIST,RT,STD |
| Decanal | 28312 | 154494 | 51606 | 36514 | 64847 | sweet, aldehydic, floral | 41 | MS,NIST,RT,STD |
| **ALCOHOL** | | | | | | | | |
| Butanol | 163276 | 358959 | nd | nd | nd | sweet, fermented, oily | 31 | MS,NIST,RT,STD |
| 3-Methylbutanol | 129553 | 264241 | nd | nd | 185233 | musty, vegetable, cocoa | 42 | MS,NIST,RT,STD |
| 1-Pentanol | 1085834 | 2612451 | 1207651 | 1110644 | 119298 | sweet, fermented, yeasty | 31 | MS,NIST,RT,STD |
| 1-Hexanol | 11044734 | 18362955 | 408313 | 451674 | 330037 | sweet, pungent, herbal | 56 | MS,NIST,RT,STD |
| 1-Octen-3-ol | 436572 | 763191 | 1265637 | 846175 | 273829 | vegetable, mushroom, chicken | 57 | MS,NIST,RT,STD |
| Maltol | 12077 | 319546 | nd | nd | 40925 | sweet, cotton candy, caramellic | 71 | MS,NIST |
| **KETONE** | | | | | | | | |
| 2-Butanone | 63489 | 510591 | nd | nd | nd | camphoreous, acetone, fruity | 72 | MS,NIST,RT,STD |
| 2-Heptanone | 60496 | 262756 | 119953 | 163355 | 71242 | sweet, spicy, banana | 58 | MS,NIST,RT,STD |
| 6-Methyl-5-hepten-2-one | 1431 | 123043 | nd | nd | 7971 | musty, banana, fruity | 108 | MS,NIST,RT,STD |
| 3,5-Octadien-2-one | 47960 | 166099 | 24386 | 24711 | nd | fruity, green, grassy | 95 | MS,NIST |
| **AROMATIC COMPOUNDS** | | | | | | | | |
| 2-Ethylfuran | 133628 | 832930 | 150063 | 133044 | 159925 | malty, cocoa, nutty | 81 | MS,NIST,RT,STD |
| o-Xylene | 76080 | 157882 | nd | nd | nd | geranium | 91 | MS,NIST,RT,STD |
| Styrene | 6527 | 27986 | nd | nd | nd | sweet, plastic, floral | 104 | MS,NIST,RT,STD |
| Geosmin | nd | nd | nd | nd | nd | musty, earthy, fresh | 112 | MS,NIST,RT,STD |
| Naphthalene | 98919 | 155721 | 16911 | 21777 | nd | dry, resinous, pungent | 128 | MS,NIST |
| 2-Pentyl furan | 912195 | 1153821 | 1263940 | 1105033 | 883548 | Fruity, green, earthy beany | 81 | MS,NIST |
| **TERPENOIDS** | | | | | | | | |
| L-limonene | 8189 | 13167 | nd | nd | nd | camphoreous, herbal, terpenic | 136 | MS,NIST,RT,STD |
| **ALKANES** | | | | | | | | |
| Decane | 4125 | nd | nd | nd | nd | unknown | 71 | MS,NIST,RT,STD |
| **SULFUR COMPOUNDS** | | | | | | | | |
| Dimethyl Disulfide | 35450 | 13910 | nd | 18890 | 32782 | vegetable, onion, cabbage | 94 | MS,NIST,RT,STD |
| Methional | nd | nd | nd | nd | nd | cabbage, pungent | 48 | MS,NIST,RT,STD |
| Methanethiol | nd | 79055 | 40563 | 36078 | 69970 | vegetable, sulfurous, eggy | 48 | MS,NIST |
| 1-(Methylthio)-propane | nd | 67203 | nd | nd | nd | garlic, acidic | 61 | MS,NIST |
| **NITROGEN COMPOUNDS** | | | | | | | | |
| 2-butyl-3,5-dimethyl pyrazine | nd | nd | nd | nd | nd | roasted, nut flavor | 122 | MS,NIST |
| 2,5-Dimethyl pyrazine | nd | 101979 | nd | nd | nd | nutty, peanut, musty | 108 | MS,NIST,RT,STD |
| **Cranberry cv. ‘CR1801-2-2’ (2022)** | | | | | | | | |
|  | **Average Area Counts** | | | | |  |  |  |
| **Compound Name** | **NRF** | **RF** | **NRP** | **RP** | **BP** | **Odor profiles from (The Good Scents Company, 2025)** | **Unique Mass (m/z)** | **Annotation** |
| **ALDEHYDE** | | | | | | | | |
| 2-Methyl butanal | 7366 | 605972 | 47990 | 85313 | 176145 | malty, musty, fermented | 57 | MS,NIST,RT,STD |
| Hexanal | 3646 | 3889775 | 805634 | 394220 | 522771 | vegetable, aldehydic, clean | 57 | MS,NIST,RT,STD |
| (E)-2-hexenal | 6668 | 69343 | 921232 | 197656 | 72485 | sweet, vegetable, bitter almond | 55 | MS,NIST,RT,STD |
| Heptanal | 5034 | 187041 | 25166 | 18803 | 26136 | aldehydic, fatty, herbal | 70 | MS,NIST,RT,STD |
| Benzaldehyde | 4138 | 596593 | 1138636 | 1325034 | 670093 | sweet, cherry, nutty | 77 | MS,NIST,RT,STD |
| Octanal | 6173 | 554017 | 49373 | 46005 | 90007 | aldehydic, fatty, herbal | 44 | MS,NIST,RT,STD |
| Nonanal | 5684 | 1795543 | 234609 | 240270 | 712138 | aldehydic, fatty, rose | 57 | MS,NIST,RT,STD |
| Decanal | 4686 | 235197 | 44687 | 66588 | 61279 | sweet, aldehydic, floral | 41 | MS,NIST,RT,STD |
| **ALCOHOL** | | | | | | | | |
| Butanol | 4475 | 242529 | nd | nd | nd | sweet, fermented, oily | 31 | MS,NIST,RT,STD |
| 3-Methylbutanol | 4455 | 392259 | 638756 | 631419 | 103455 | musty, vegetable, cocoa | 42 | MS,NIST,RT,STD |
| 1-Pentanol | 4192 | 330206 | 639093 | 55356 | nd | sweet, fermented, yeasty | 31 | MS,NIST,RT,STD |
| 1-Hexanol | 4869 | 365127 | 634028 | 467811 | 168257 | sweet, pungent, herbal | 56 | MS,NIST,RT,STD |
| 1-Octen-3-ol | 1993 | 246913 | 1510088 | 950002 | 304784 | vegetable, mushroom, chicken | 57 | MS,NIST,RT,STD |
| Maltol | nd | 257260 | 25273 | 78275 | 29985 | sweet, cotton candy, caramellic | 71 | MS,NIST |
| **KETONE** | | | | | | | | |
| 2-Butanone | 63978 | 716197 | 8974 | 6334 | 15579 | camphoreous, acetone, fruity | 72 | MS,NIST,RT,STD |
| 2-Heptanone | nd | 144973 | 158612 | nd | 55297 | sweet, spicy, banana | 58 | MS,NIST,RT,STD |
| 6-Methyl-5-hepten-2-one | 6969 | 485307 | 21805 | 6269 | nd | musty, banana, fruity | 108 | MS,NIST,RT,STD |
| 3,5-Octadien-2-one | 122924 | 100035 | 249504 | 66106 | 27714 | fruity, green, grassy | 95 | MS,NIST |
| **AROMATIC COMPOUNDS** | | | | | | | | |
| 2-Ethylfuran | 3658 | 473033 | 918454 | 225732 | 409060 | malty, cocoa, nutty | 81 | MS,NIST,RT,STD |
| o-Xylene | 9248 | 181061 | 51782 | nd | 93453 | geranium | 91 | MS,NIST,RT,STD |
| Styrene | 2459 | 145276 | nd | nd | 32264 | sweet, plastic, floral | 104 | MS,NIST,RT,STD |
| Geosmin | nd | nd | nd | nd | nd | musty, earthy, fresh | 112 | MS,NIST,RT,STD |
| Azulene/Naphthalene | 48645 | 57671 | 37872 | 34197 | 23163 | dry, resinous, pungent | 128 | MS,NIST |
| 2-Pentyl furan | 233677 | 149113 | 614006 | 281804 | 556938 | Fruity, green, earthy beany | 81 | MS,NIST |
| **TERPENOIDS** | | | | | | | | |
| L-limonene | 3048 | 84152 | nd | nd | 15951 | camphoreous, herbal, terpenic | 136 | MS,NIST,RT,STD |
| **ALKANES** | | | | | | | | |
| Decane | nd | 21315 | 3122 | nd | nd | unknown | 71 | MS,NIST,RT,STD |
| **SULFUR COMPOUNDS** | | | | | | | | |
| Dimethyl Disulfide | nd | 356675 | 7277465 | 2964252 | 120469 | vegetable, onion, cabbage | 94 | MS,NIST,RT,STD |
| Methional | nd | nd | nd | nd | nd | cabbage, pungent | 48 | MS,NIST,RT,STD |
| Methanethiol | nd | 308985 | nd | 152323 | 176395 | vegetable, sulfurous, eggy | 48 | MS,NIST |
| 1-(Methylthio)-propane | nd | 617060 | nd | 123988 | nd | garlic, acidic | 61 | MS,NIST |
| **NITROGEN COMPOUNDS** | | | | | | | | |
| 2-butyl-3,5-dimethyl pyrazine | nd | 32931 | nd | 36962 | nd | roasted, nut flavor | 122 | MS,NIST |
| 2,5-Dimethyl pyrazine | nd | 57031 | nd | 47618 | nd | nutty, peanut, musty | 108 | MS,NIST,RT,STD |
| **Great Northern cv. ‘Powderhorn’ (2022)** | | | | | | | | |
|  | **Average Area Counts** | | | | |  |  |  |
| **Compound Name** | **NRF** | **RF** | **NRP** | **RP** | **BP** | **Odor profiles from (The Good Scents Company, 2025)** | **Unique Mass (m/z)** | **Annotation** |
| **ALDEHYDE** | | | | | | | | |
| 2-Methyl butanal | 107960 | 255189 | 152485 | 397480 | nd | malty, musty, fermented | 57 | MS,NIST,RT,STD |
| Hexanal | 286932 | 1545905 | 5619434 | 310420 | 114120 | vegetable, aldehydic, clean | 57 | MS,NIST,RT,STD |
| (E)-2-hexenal | 27384 | 40069 | 1560337 | 310623 | 557763 | sweet, vegetable, bitter almond | 55 | MS,NIST,RT,STD |
| Heptanal | 38297 | 86449 | 102039 | 20071 | nd | aldehydic, fatty, herbal | 70 | MS,NIST,RT,STD |
| Benzaldehyde | 168272 | 475337 | 2596801 | 1020132 | 526200 | sweet, cherry, nutty | 77 | MS,NIST,RT,STD |
| Octanal | 140995 | 218361 | 91133 | 27252 | nd | aldehydic, fatty, herbal | 44 | MS,NIST,RT,STD |
| Nonanal | 535384 | 549993 | 295342 | 108430 | 51410 | aldehydic, fatty, rose | 57 | MS,NIST,RT,STD |
| Decanal | 55724 | 66589 | 73738 | 30725 | 27487 | sweet, aldehydic, floral | 41 | MS,NIST,RT,STD |
| **ALCOHOL** | | | | | | | | |
| Butanol | 226002 | 187806 | 3712206 | nd | nd | sweet, fermented, oily | 31 | MS,NIST,RT,STD |
| 3-Methylbutanol | 95225 | 818890 | 1539601 | 955558 | 58845 | musty, vegetable, cocoa | 42 | MS,NIST,RT,STD |
| 1-Pentanol | 161559 | 136501 | 742175 | 100868 | nd | sweet, fermented, yeasty | 31 | MS,NIST,RT,STD |
| 1-Hexanol | 934682 | 525992 | 5251450 | 1790015 | 272575 | sweet, pungent, herbal | 56 | MS,NIST,RT,STD |
| 1-Octen-3-ol | 302679 | 381264 | 488263 | 138242 | 109850 | vegetable, mushroom, chicken | 57 | MS,NIST,RT,STD |
| Maltol | nd | 190173 | nd | 34975 | 22650 | sweet, cotton candy, caramellic | 71 | MS,NIST |
| **KETONE** | | | | | | | | |
| 2-Butanone | 15642 | 209978 | 6423 | 102178 | nd | camphoreous, acetone, fruity | 72 | MS,NIST,RT,STD |
| 2-Heptanone | 31521 | 98735 | 53243 | nd | nd | sweet, spicy, banana | 58 | MS,NIST,RT,STD |
| 6-Methyl-5-hepten-2-one | 11900 | nd | nd | nd | nd | musty, banana, fruity | 108 | MS,NIST,RT,STD |
| 3,5-Octadien-2-one | 57375 | 50846 | 127339 | 43395 | 30326 | fruity, green, grassy | 95 | MS,NIST |
| **AROMATIC COMPOUNDS** | | | | | | | | |
| 2-Ethylfuran | 70990 | 308425 | 395844 | 61564 | 433483 | malty, cocoa, nutty | 81 | MS,NIST,RT,STD |
| o-Xylene | 78154 | 84683 | nd | nd | 7023 | geranium | 91 | MS,NIST,RT,STD |
| Styrene | 56635 | 49280 | nd | nd | nd | sweet, plastic, floral | 104 | MS,NIST,RT,STD |
| Geosmin | nd | nd | nd | nd | nd | musty, earthy, fresh | 112 | MS,NIST,RT,STD |
| Azulene/Naphthalene | 25298 | 27943 | 14625 | 15851 | 164797 | dry, resinous, pungent | 128 | MS,NIST |
| 2-Pentyl furan | 115889 | 163351 | 157412 | 97704 | 369884 | Fruity, green, earthy beany | 81 | MS,NIST |
| **TERPENOIDS** | | | | | | | | |
| L-limonene | 18159 | 1804 | nd | nd | nd | camphoreous, herbal, terpenic | 136 | MS,NIST,RT,STD |
| **ALKANES** | | | | | | | | |
| Decane | 41332 | nd | nd | nd | nd | unknown | 71 | MS,NIST,RT,STD |
| **SULFUR COMPOUNDS** | | | | | | | | |
| Dimethyl Disulfide | nd | 122274 | 21397 | 7589305 | 585719 | vegetable, onion, cabbage | 94 | MS,NIST,RT,STD |
| Methional | nd | nd | nd | nd | nd | cabbage, pungent | 48 | MS,NIST,RT,STD |
| Methanethiol | nd | 89141 | nd | nd | 4722199 | vegetable, sulfurous, eggy | 48 | MS,NIST |
| 1-(Methylthio)-propane | nd | 139308 | nd | nd | nd | garlic, acidic | 61 | MS,NIST |
| **NITROGEN COMPOUNDS** | | | | | | | | |
| 2-butyl-3,5-dimethyl pyrazine | nd | nd | nd | nd | nd | roasted, nut flavor | 122 | MS,NIST |
| 2,5-Dimethyl pyrazine | nd | nd | nd | nd | nd | nutty, peanut, musty | 108 | MS,NIST,RT,STD |
| **Manteca cv. ‘Y1608-07’ (2022)** | | | | | | | | |
|  | **Average Area Counts** | | | | |  |  |  |
| **Compound Name** | **NRF** | **RF** | **NRP** | **RP** | **BP** | **Odor profiles from (The Good Scents Company, 2025)** | **Unique Mass (m/z)** | **Annotation** |
| **ALDEHYDE** | | | | | | | | |
| 2-Methyl butanal | 641577 | 995256 | 175534 | 236384 | 65887 | malty, musty, fermented | 57 | MS,NIST,RT,STD |
| Hexanal | 8152275 | 5720436 | 4406276 | 1957748 | 628207 | vegetable, aldehydic, clean | 57 | MS,NIST,RT,STD |
| (E)-2-hexenal | 192588 | 105363 | 2314312 | 1118826 | 93873 | sweet, vegetable, bitter almond | 55 | MS,NIST,RT,STD |
| Heptanal | 458260 | 324769 | 87698 | 85716 | 42888 | aldehydic, fatty, herbal | 70 | MS,NIST,RT,STD |
| Benzaldehyde | 2017294 | 2326193 | 1826027 | 2170174 | 980601 | sweet, cherry, nutty | 77 | MS,NIST,RT,STD |
| Octanal | 584212 | 635472 | 140761 | 162961 | 87930 | aldehydic, fatty, herbal | 44 | MS,NIST,RT,STD |
| Nonanal | 1913750 | 2413253 | 506170 | 701670 | 315002 | aldehydic, fatty, rose | 57 | MS,NIST,RT,STD |
| Decanal | 125114 | 314905 | 99989 | 145333 | 53855 | sweet, aldehydic, floral | 41 | MS,NIST,RT,STD |
| **ALCOHOL** | | | | | | | | |
| Butanol | 341673 | 702868 | 1959916 | nd | 8630 | sweet, fermented, oily | 31 | MS,NIST,RT,STD |
| 3-Methylbutanol | 72552 | 2792408 | 488632 | 757423 | nd | musty, vegetable, cocoa | 42 | MS,NIST,RT,STD |
| 1-Pentanol | 243126 | 694325 | 317468 | 102069 | nd | sweet, fermented, yeasty | 31 | MS,NIST,RT,STD |
| 1-Hexanol | 714820 | 1180427 | 2959073 | 1247426 | 169031 | sweet, pungent, herbal | 56 | MS,NIST,RT,STD |
| 1-Octen-3-ol | 1797541 | 594486 | 1500354 | 1104172 | 215479 | vegetable, mushroom, chicken | 57 | MS,NIST,RT,STD |
| Maltol | 262435 | 928096 | 34242 | 60912 | 79463 | sweet, cotton candy, caramellic | 71 | MS,NIST |
| **KETONE** | | | | | | | | |
| 2-Butanone | 773333 | 917975 | 9777 | 7672 | 15273 | camphoreous, acetone, fruity | 72 | MS,NIST,RT,STD |
| 2-Heptanone | 315825 | 405274 | 66874 | 70534 | nd | sweet, spicy, banana | 58 | MS,NIST,RT,STD |
| 6-Methyl-5-hepten-2-one | nd | nd | 37342 | 20012 | nd | musty, banana, fruity | 108 | MS,NIST,RT,STD |
| 3,5-Octadien-2-one | 480511 | 183886 | 184725 | 109142 | nd | fruity, green, grassy | 95 | MS,NIST |
| **AROMATIC COMPOUNDS** | | | | | | | | |
| 2-Ethylfuran | 1268046 | 3322132 | 681984 | 599096 | 313949 | malty, cocoa, nutty | 81 | MS,NIST,RT,STD |
| o-Xylene | 181182 | 94736 | 80397 | 65433 | nd | geranium | 91 | MS,NIST,RT,STD |
| Styrene | 155883 | 29529 | nd | nd | nd | sweet, plastic, floral | 104 | MS,NIST,RT,STD |
| Geosmin | nd | nd | nd | nd | nd | musty, earthy, fresh | 112 | MS,NIST,RT,STD |
| Azulene/Naphthalene | 73870 | 82094 | 49073 | 57742 | nd | dry, resinous, pungent | 128 | MS,NIST |
| 2-Pentyl furan | 799041 | 547490 | 301726 | 236278 | 216342 | Fruity, green, earthy beany | 81 | MS,NIST |
| **TERPENOIDS** | | | | | | | | |
| L-limonene | 44591 | 7475 | nd | nd | nd | camphoreous, herbal, terpenic | 136 | MS,NIST,RT,STD |
| **ALKANES** | | | | | | | | |
| Decane | nd | 13898 | nd | nd | nd | unknown | 71 | MS,NIST,RT,STD |
| **SULFUR COMPOUNDS** | | | | | | | | |
| Dimethyl Disulfide | 84440 | 833995 | 15875 | 9873385 | 108515 | vegetable, onion, cabbage | 94 | MS,NIST,RT,STD |
| Methional | nd | 8624 | nd | nd | nd | cabbage, pungent | 48 | MS,NIST,RT,STD |
| Methanethiol | 60758 | 687294 | nd | 117765 | 294419 | vegetable, sulfurous, eggy | 48 | MS,NIST |
| 1-(Methylthio)-propane | nd | 222452 | nd | nd | nd | garlic, acidic | 61 | MS,NIST |
| **NITROGEN COMPOUNDS** | | | | | | | | |
| 2-butyl-3,5-dimethyl pyrazine | nd | nd | nd | nd | nd | roasted, nut flavor | 122 | MS,NIST |
| 2,5-Dimethyl pyrazine | nd | 34574 | nd | 21276 | nd | nutty, peanut, musty | 108 | MS,NIST,RT,STD |
| **Mayacoba cv. ‘Y 1802-9-1’ (2022)** | | | | | | | | |
|  | **Average Area Counts** | | | | |  |  |  |
| **Compound Name** | **NRF** | **RF** | **NRP** | **RP** | **BP** | **Odor profiles from (The Good Scents Company, 2025)** | **Unique Mass (m/z)** | **Annotation** |
| **ALDEHYDE** | | | | | | | | |
| 2-Methyl butanal | 230630 | 1307858 | 36863 | 125937 | 26684 | malty, musty, fermented | 57 | MS,NIST,RT,STD |
| Hexanal | 4529413 | 7708738 | 1848296 | 815824 | 255453 | vegetable, aldehydic, clean | 57 | MS,NIST,RT,STD |
| (E)-2-hexenal | 169767 | 338680 | 33770 | 179054 | nd | sweet, vegetable, bitter almond | 55 | MS,NIST,RT,STD |
| Heptanal | 318562 | 739587 | 53215 | 61231 | 27795 | aldehydic, fatty, herbal | 70 | MS,NIST,RT,STD |
| Benzaldehyde | 613117 | 3590426 | 1717543 | 1263318 | 747102 | sweet, cherry, nutty | 77 | MS,NIST,RT,STD |
| Octanal | 446851 | 1107052 | 52429 | 87892 | 55971 | aldehydic, fatty, herbal | 44 | MS,NIST,RT,STD |
| Nonanal | 2086547 | 3328626 | 239492 | 548963 | 193681 | aldehydic, fatty, rose | 57 | MS,NIST,RT,STD |
| Decanal | 172584 | 352404 | 41280 | 123659 | 23689 | sweet, aldehydic, floral | 41 | MS,NIST,RT,STD |
| **ALCOHOL** | | | | | | | | |
| Butanol | 231560 | 561931 | 36021 | 95326 | 91846 | sweet, fermented, oily | 31 | MS,NIST,RT,STD |
| 3-Methylbutanol | 129003 | 1729344 | 970770 | 220270 | 111748 | musty, vegetable, cocoa | 42 | MS,NIST,RT,STD |
| 1-Pentanol | 321763 | 493390 | 584087 | 69477 | 47191 | sweet, fermented, yeasty | 31 | MS,NIST,RT,STD |
| 1-Hexanol | 805099 | 929969 | 3179344 | 749360 | 728408 | sweet, pungent, herbal | 56 | MS,NIST,RT,STD |
| 1-Octen-3-ol | 482459 | 1149006 | 2021131 | 575402 | 280688 | vegetable, mushroom, chicken | 57 | MS,NIST,RT,STD |
| Maltol | nd | 766650 | 19543 | 72544 | 37394 | sweet, cotton candy, caramellic | 71 | MS,NIST |
| **KETONE** | | | | | | | | |
| 2-Butanone | 257447 | 1609235 | 19584 | 10437 | 92954 | camphoreous, acetone, fruity | 72 | MS,NIST,RT,STD |
| 2-Heptanone | 100681 | 421925 | 85759 | 52193 | 96645 | sweet, spicy, banana | 58 | MS,NIST,RT,STD |
| 6-Methyl-5-hepten-2-one | 16081 | nd | nd | nd | nd | musty, banana, fruity | 108 | MS,NIST,RT,STD |
| 3,5-Octadien-2-one | 364292 | 619023 | 570967 | 118622 | nd | fruity, green, grassy | 95 | MS,NIST |
| **AROMATIC COMPOUNDS** | | | | | | | | |
| 2-Ethylfuran | 605985 | 2488932 | 1776885 | 137328 | 328096 | malty, cocoa, nutty | 81 | MS,NIST,RT,STD |
| o-Xylene | 45247 | 161077 | nd | 1252 | nd | geranium | 91 | MS,NIST,RT,STD |
| Styrene | 4948 | 66396 | nd | nd | nd | sweet, plastic, floral | 104 | MS,NIST,RT,STD |
| Geosmin | nd | nd | nd | nd | nd | musty, earthy, fresh | 112 | MS,NIST,RT,STD |
| Azulene/Naphthalene | 97465 | 132576 | 46288 | 42867 | nd | dry, resinous, pungent | 128 | MS,NIST |
| 2-Pentyl furan | 197354 | 760406 | 391568 | 134081 | 558060 | Fruity, green, earthy beany | 81 | MS,NIST |
| **TERPENOIDS** | | | | | | | | |
| L-limonene | 65600 | 10667 | nd | nd | nd | camphoreous, herbal, terpenic | 136 | MS,NIST,RT,STD |
| **ALKANES** | | | | | | | | |
| Decane | 6607 | nd | 7363 | nd | 4604 | unknown | 71 | MS,NIST,RT,STD |
| **SULFUR COMPOUNDS** | | | | | | | | |
| Dimethyl Disulfide | 10171 | 439084 | 28862 | 322389 | 3498334 | vegetable, onion, cabbage | 94 | MS,NIST,RT,STD |
| Methional | nd | nd | nd | 24639 | nd | cabbage, pungent | 48 | MS,NIST,RT,STD |
| Methanethiol | nd | 353266 | 27468 | 362696 | 1729830 | vegetable, sulfurous, eggy | 48 | MS,NIST |
| 1-(Methylthio)-propane | nd | 1147190 | nd | 70490 | nd | garlic, acidic | 61 | MS,NIST |
| **NITROGEN COMPOUNDS** | | | | | | | | |
| 2-butyl-3,5-dimethyl pyrazine | nd | 112765 | nd | nd | nd | roasted, nut flavor | 122 | MS,NIST |
| 2,5-Dimethyl pyrazine | nd | 221711 | nd | 39892 | nd | nutty, peanut, musty | 108 | MS,NIST,RT,STD |
| **Navy cv. ‘Alpena’ (2022)** | | | | | | | | |
|  | **Average Area Counts** | | | | |  |  |  |
| **Compound Name** | **NRF** | **RF** | **NRP** | **RP** | **BP** | **Odor profiles from (The Good Scents Company, 2025)** | **Unique Mass (m/z)** | **Annotation** |
| **ALDEHYDE** | | | | | | | | |
| 2-Methyl butanal | 113106 | 267493 | 11348 | 4395 | nd | malty, musty, fermented | 57 | MS,NIST,RT,STD |
| Hexanal | 1389407 | 3528105 | 2641011 | 355621 | 224302 | vegetable, aldehydic, clean | 57 | MS,NIST,RT,STD |
| (E)-2-hexenal | nd | nd | 4453950 | 587954 | nd | sweet, vegetable, bitter almond | 55 | MS,NIST,RT,STD |
| Heptanal | 134124 | 299721 | 169401 | 20192 | 30116 | aldehydic, fatty, herbal | 70 | MS,NIST,RT,STD |
| Benzaldehyde | 318457 | 765632 | 1443147 | 842859 | 285756 | sweet, cherry, nutty | 77 | MS,NIST,RT,STD |
| Octanal | 304444 | 549158 | 107582 | 34686 | 42280 | aldehydic, fatty, herbal | 44 | MS,NIST,RT,STD |
| Nonanal | 603167 | 1369214 | 444076 | 126541 | 181318 | aldehydic, fatty, rose | 57 | MS,NIST,RT,STD |
| Decanal | 59057 | 132426 | 85007 | 30448 | 34987 | sweet, aldehydic, floral | 41 | MS,NIST,RT,STD |
| **ALCOHOL** | | | | | | | | |
| Butanol | 117532 | 108082 | nd | 123260 | 36093 | sweet, fermented, oily | 31 | MS,NIST,RT,STD |
| 3-Methylbutanol | nd | 155224 | 144508 | 1163442 | nd | musty, vegetable, cocoa | 42 | MS,NIST,RT,STD |
| 1-Pentanol | 41940 | 85353 | 521750 | 333857 | nd | sweet, fermented, yeasty | 31 | MS,NIST,RT,STD |
| 1-Hexanol | 124256 | 166731 | 6329960 | 2738191 | 81589 | sweet, pungent, herbal | 56 | MS,NIST,RT,STD |
| 1-Octen-3-ol | 144811 | 487518 | 978103 | 525501 | 33071 | vegetable, mushroom, chicken | 57 | MS,NIST,RT,STD |
| Maltol | 30662 | 141453 | nd | 71275 | 25676 | sweet, cotton candy, caramellic | 71 | MS,NIST |
| **KETONE** | | | | | | | | |
| 2-Butanone | 120068 | 276747 | 12191 | 171437 | 60887 | camphoreous, acetone, fruity | 72 | MS,NIST,RT,STD |
| 2-Heptanone | 37488 | 83037 | 85055 | 64895 | nd | sweet, spicy, banana | 58 | MS,NIST,RT,STD |
| 6-Methyl-5-hepten-2-one | 11116 | 38888 | nd | nd | 2688 | musty, banana, fruity | 108 | MS,NIST,RT,STD |
| 3,5-Octadien-2-one | 74418 | 139122 | 268990 | 219101 | 20289 | fruity, green, grassy | 95 | MS,NIST |
| **AROMATIC COMPOUNDS** | | | | | | | | |
| 2-Ethylfuran | 149402 | 357234 | 3966078 | 753359 | 124337 | malty, cocoa, nutty | 81 | MS,NIST,RT,STD |
| o-Xylene | 59977 | 71268 | nd | 615655 | nd | geranium | 91 | MS,NIST,RT,STD |
| Styrene | 27007 | 25698 | nd | nd | nd | sweet, plastic, floral | 104 | MS,NIST,RT,STD |
| Geosmin | nd | nd | nd | nd | nd | musty, earthy, fresh | 112 | MS,NIST,RT,STD |
| Azulene/Naphthalene | 28567 | 59148 | 40786 | 38695 | 55765 | dry, resinous, pungent | 128 | MS,NIST |
| 2-Pentyl furan | 194374 | 151859 | 933425 | 475306 | 121657 | Fruity, green, earthy beany | 81 | MS,NIST |
| **TERPENOIDS** | | | | | | | | |
| L-limonene | 12687 | 3137 | nd | nd | nd | camphoreous, herbal, terpenic | 136 | MS,NIST,RT,STD |
| **ALKANES** | | | | | | | | |
| Decane | nd | nd | nd | nd | nd | unknown | 71 | MS,NIST,RT,STD |
| **SULFUR COMPOUNDS** | | | | | | | | |
| Dimethyl Disulfide | nd | 21833 | 210415 | 10994809 | 15910 | vegetable, onion, cabbage | 94 | MS,NIST,RT,STD |
| Methional | nd | nd | nd | 10449 | nd | cabbage, pungent | 48 | MS,NIST,RT,STD |
| Methanethiol | nd | 63703 | nd | 1040960 | 243635 | vegetable, sulfurous, eggy | 48 | MS,NIST |
| 1-(Methylthio)-propane | nd | 194028 | nd | nd | nd | garlic, acidic | 61 | MS,NIST |
| **NITROGEN COMPOUNDS** | | | | | | | | |
| 2-butyl-3,5-dimethyl pyrazine | nd | nd | nd | nd | nd | roasted, nut flavor | 122 | MS,NIST |
| 2,5-Dimethyl pyrazine | nd | nd | nd | 50999 | nd | nutty, peanut, musty | 108 | MS,NIST,RT,STD |
| **Otebo cv. ‘Samurai’ (2022)** | | | | | | | | |
|  | **Average Area Counts** | | | | |  |  |  |
| **Compound Name** | **NRF** | **RF** | **NRP** | **RP** | **BP** | **Odor profiles from (The Good Scents Company, 2025)** | **Unique Mass (m/z)** | **Annotation** |
| **ALDEHYDE** | | | | | | | | |
| 2-Methyl butanal | 136764 | 630763 | 552012 | 16013 | nd | malty, musty, fermented | 57 | MS,NIST,RT,STD |
| Hexanal | 574644 | 5615175 | 4418144 | 1402058 | 161843 | vegetable, aldehydic, clean | 57 | MS,NIST,RT,STD |
| (E)-2-hexenal | 56430 | 229338 | 1989198 | 314557 | nd | sweet, vegetable, bitter almond | 55 | MS,NIST,RT,STD |
| Heptanal | 53323 | 712577 | 161733 | 65019 | 6028 | aldehydic, fatty, herbal | 70 | MS,NIST,RT,STD |
| Benzaldehyde | 142817 | 2713296 | 1453781 | 1413045 | 177428 | sweet, cherry, nutty | 77 | MS,NIST,RT,STD |
| Octanal | 102533 | 690393 | 153462 | 118514 | nd | aldehydic, fatty, herbal | 44 | MS,NIST,RT,STD |
| Nonanal | 286261 | 3935318 | 407815 | 449648 | 77595 | aldehydic, fatty, rose | 57 | MS,NIST,RT,STD |
| Decanal | 46754 | 293342 | 53490 | 65732 | 22515 | sweet, aldehydic, floral | 41 | MS,NIST,RT,STD |
| **ALCOHOL** | | | | | | | | |
| Butanol | 97961 | 795204 | nd | 88868 | nd | sweet, fermented, oily | 31 | MS,NIST,RT,STD |
| 3-Methylbutanol | 98269 | 1131637 | 36545 | 49735 | nd | musty, vegetable, cocoa | 42 | MS,NIST,RT,STD |
| 1-Pentanol | 102295 | 461710 | 318322 | 48799 | nd | sweet, fermented, yeasty | 31 | MS,NIST,RT,STD |
| 1-Hexanol | 253106 | 794733 | 2745521 | 821428 | nd | sweet, pungent, herbal | 56 | MS,NIST,RT,STD |
| 1-Octen-3-ol | 135693 | 630554 | 326447 | 159584 | nd | vegetable, mushroom, chicken | 57 | MS,NIST,RT,STD |
| Maltol | 33921 | 904566 | nd | 47085 | 40810 | sweet, cotton candy, caramellic | 71 | MS,NIST |
| **KETONE** | | | | | | | | |
| 2-Butanone | 23025 | 816949 | nd | 4174 | 3075 | camphoreous, acetone, fruity | 72 | MS,NIST,RT,STD |
| 2-Heptanone | 21462 | 412575 | 38879 | nd | nd | sweet, spicy, banana | 58 | MS,NIST,RT,STD |
| 6-Methyl-5-hepten-2-one | nd | nd | nd | nd | nd | musty, banana, fruity | 108 | MS,NIST,RT,STD |
| 3,5-Octadien-2-one | 48019 | 501769 | 160480 | 62965 | nd | fruity, green, grassy | 95 | MS,NIST |
| **AROMATIC COMPOUNDS** | | | | | | | | |
| 2-Ethylfuran | 104300 | 2183831 | 509727 | 105915 | 53023 | malty, cocoa, nutty | 81 | MS,NIST,RT,STD |
| o-Xylene | 33986 | 217909 | nd | nd | nd | geranium | 91 | MS,NIST,RT,STD |
| Styrene | 5463 | 122899 | nd | nd | nd | sweet, plastic, floral | 104 | MS,NIST,RT,STD |
| Geosmin | nd | nd | nd | nd | nd | musty, earthy, fresh | 112 | MS,NIST,RT,STD |
| Azulene/Naphthalene | 27706 | 105847 | 19158 | 20159 | 70899 | dry, resinous, pungent | 128 | MS,NIST |
| 2-Pentyl furan | 94119 | 509603 | 187385 | 88493 | 106333 | Fruity, green, earthy beany | 81 | MS,NIST |
| **TERPENOIDS** | | | | | | | | |
| L-limonene | nd | 9676 | nd | 18316 | nd | camphoreous, herbal, terpenic | 136 | MS,NIST,RT,STD |
| **ALKANES** | | | | | | | | |
| Decane | nd | 41278 | nd | nd | nd | unknown | 71 | MS,NIST,RT,STD |
| **SULFUR COMPOUNDS** | | | | | | | | |
| Dimethyl Disulfide | 48526 | 442293 | 5704 | 3899889 | 11780 | vegetable, onion, cabbage | 94 | MS,NIST,RT,STD |
| Methional | nd | 11695 | nd | nd | nd | cabbage, pungent | 48 | MS,NIST,RT,STD |
| Methanethiol | nd | 426476 | nd | 86511 | 222485 | vegetable, sulfurous, eggy | 48 | MS,NIST |
| 1-(Methylthio)-propane | nd | 877153 | nd | nd | nd | garlic, acidic | 61 | MS,NIST |
| **NITROGEN COMPOUNDS** | | | | | | | | |
| 2-butyl-3,5-dimethyl pyrazine | nd | nd | nd | nd | nd | roasted, nut flavor | 122 | MS,NIST |
| 2,5-Dimethyl pyrazine | nd | 199851 | nd | nd | nd | nutty, peanut, musty | 108 | MS,NIST,RT,STD |
| **White kidney cv. ‘WK 1601-1’ (2022)** | | | | | | | | |
|  | **Average Area Counts** | | | | |  |  |  |
| **Compound Name** | **NRF** | **RF** | **NRP** | **RP** | **BP** | **Odor profiles from (The Good Scents Company, 2025)** | **Unique Mass (m/z)** | **Annotation** |
| **ALDEHYDE** | | | | | | | | |
| 2-Methyl butanal | 77558 | 722699 | 9204506 | 263943 | 69772 | malty, musty, fermented | 57 | MS,NIST,RT,STD |
| Hexanal | 1607810 | 2390494 | 3210856 | 456766 | 86759 | vegetable, aldehydic, clean | 57 | MS,NIST,RT,STD |
| (E)-2-hexenal | 59637 | 72253 | 3874239 | 152127 | nd | sweet, vegetable, bitter almond | 55 | MS,NIST,RT,STD |
| Heptanal | 113431 | 265367 | 116785 | 26173 | nd | aldehydic, fatty, herbal | 70 | MS,NIST,RT,STD |
| Benzaldehyde | 496110 | 867235 | 617729 | 1524460 | 513599 | sweet, cherry, nutty | 77 | MS,NIST,RT,STD |
| Octanal | 335186 | 666273 | 44742 | 71408 | 28835 | aldehydic, fatty, herbal | 44 | MS,NIST,RT,STD |
| Nonanal | 1073351 | 1587980 | 188414 | 281497 | 125657 | aldehydic, fatty, rose | 57 | MS,NIST,RT,STD |
| Decanal | 165507 | 227961 | 43141 | 60268 | nd | sweet, aldehydic, floral | 41 | MS,NIST,RT,STD |
| **ALCOHOL** | | | | | | | | |
| Butanol | 288984 | 268827 | 1337687 | 235358 | 221446 | sweet, fermented, oily | 31 | MS,NIST,RT,STD |
| 3-Methylbutanol | 54600 | 480609 | 165298 | 383615 | 116249 | musty, vegetable, cocoa | 42 | MS,NIST,RT,STD |
| 1-Pentanol | 86307 | 106190 | 141015 | 34158 | 46531 | sweet, fermented, yeasty | 31 | MS,NIST,RT,STD |
| 1-Hexanol | 427561 | 338580 | 1213484 | 942608 | 1123889 | sweet, pungent, herbal | 56 | MS,NIST,RT,STD |
| 1-Octen-3-ol | 246232 | 193652 | 771812 | 1078474 | 297120 | vegetable, mushroom, chicken | 57 | MS,NIST,RT,STD |
| Maltol | 34596 | 237879 | nd | 54831 | 38362 | sweet, cotton candy, caramellic | 71 | MS,NIST |
| **KETONE** | | | | | | | | |
| 2-Butanone | 97795 | 298842 | 6813 | 13440 | 61729 | camphoreous, acetone, fruity | 72 | MS,NIST,RT,STD |
| 2-Heptanone | 64393 | 138797 | nd | 67032 | 99634 | sweet, spicy, banana | 58 | MS,NIST,RT,STD |
| 6-Methyl-5-hepten-2-one | 22560 | 25790 | nd | 10165 | nd | musty, banana, fruity | 108 | MS,NIST,RT,STD |
| 3,5-Octadien-2-one | 139121 | 54261 | 89535 | 62395 | nd | fruity, green, grassy | 95 | MS,NIST |
| **AROMATIC COMPOUNDS** | | | | | | | | |
| 2-Ethylfuran | 453941 | 1653719 | 436526 | 408338 | 270618 | malty, cocoa, nutty | 81 | MS,NIST,RT,STD |
| o-Xylene | 382658 | 128844 | nd | 52060 | nd | geranium | 91 | MS,NIST,RT,STD |
| Styrene | 151256 | 99248 | nd | nd | nd | sweet, plastic, floral | 104 | MS,NIST,RT,STD |
| Geosmin | nd | nd | nd | nd | nd | musty, earthy, fresh | 112 | MS,NIST,RT,STD |
| Azulene/Naphthalene | 51315 | 52696 | nd | 38453 | nd | dry, resinous, pungent | 128 | MS,NIST |
| 2-Pentyl furan | 189046 | 183585 | 105967 | 114361 | 360181 | Fruity, green, earthy beany | 81 | MS,NIST |
| **TERPENOIDS** | | | | | | | | |
| L-limonene | 2713 | 3216 | nd | nd | nd | camphoreous, herbal, terpenic | 136 | MS,NIST,RT,STD |
| **ALKANES** | | | | | | | | |
| Decane | 4484 | 2055 | nd | 4540 | 8039 | unknown | 71 | MS,NIST,RT,STD |
| **SULFUR COMPOUNDS** | | | | | | | | |
| Dimethyl Disulfide | nd | 345558 | 28750 | 14386294 | 280892 | vegetable, onion, cabbage | 94 | MS,NIST,RT,STD |
| Methional | nd | nd | nd | 39898 | nd | cabbage, pungent | 48 | MS,NIST,RT,STD |
| Methanethiol | nd | 157162 | nd | 32161 | 199754 | vegetable, sulfurous, eggy | 48 | MS,NIST |
| 1-(Methylthio)-propane | nd | 268098 | nd | nd | nd | garlic, acidic | 61 | MS,NIST |
| **NITROGEN COMPOUNDS** | | | | | | | | |
| 2-butyl-3,5-dimethyl pyrazine | nd | 33624 | nd | nd | nd | roasted, nut flavor | 122 | MS,NIST |
| 2,5-Dimethyl pyrazine | nd | 37863 | nd | 32016 | nd | nutty, peanut, musty | 108 | MS,NIST,RT,STD |
| **Chickpea cv. ‘Sierra’ (2023^b^)** | | | | | | | | |
|  | **Average Area Counts** | | | | |  |  |  |
| **Compound Name** | **NRF** | **RF** | **NRP** | **RP** | **BP** | **Odor profiles from (The Good Scents Company, 2025)** | **Unique Mass (m/z)** | **Annotation** |
| **ALDEHYDE** | | | | | | | | |
| 2-Methyl butanal | 59419 | 387145 | 5659 | 3419779 | 210922 | malty, musty, fermented | 57 | MS,NIST,RT,STD |
| Hexanal | 119250 | 895377 | 91852318 | 108170543 | 2862079 | vegetable, aldehydic, clean | 57 | MS,NIST,RT,STD |
| (E)-2-hexenal | 14852 | nd | 753187 | 814523 | nd | sweet, vegetable, bitter almond | 55 | MS,NIST,RT,STD |
| Heptanal | 125978 | 62116 | 998023 | 1853835 | 146996 | aldehydic, fatty, herbal | 70 | MS,NIST,RT,STD |
| Benzaldehyde | 171963 | 884624 | 2201817 | 3000758 | 1141425 | sweet, cherry, nutty | 77 | MS,NIST,RT,STD |
| Octanal | 13490 | 146186 | 608859 | 1572733 | 347284 | aldehydic, fatty, herbal | 44 | MS,NIST,RT,STD |
| Nonanal | 86657 | 645248 | 3656006 | 7880997 | 3013801 | aldehydic, fatty, rose | 57 | MS,NIST,RT,STD |
| Decanal | 36547 | 328642 | 431750 | 698617 | 162417 | sweet, aldehydic, floral | 41 | MS,NIST,RT,STD |
| **ALCOHOL** | | | | | | | | |
| Butanol | 207373 | 2250763 | 71753 | 379667 | nd | sweet, fermented, oily | 31 | MS,NIST,RT,STD |
| 3-Methylbutanol | 140286 | 1372602 | 16123719 | 17968514 | 328326 | musty, vegetable, cocoa | 42 | MS,NIST,RT,STD |
| 1-Pentanol | 1459304 | 7789526 | 15477842 | 12323314 | 98760 | sweet, fermented, yeasty | 31 | MS,NIST,RT,STD |
| 1-Hexanol | 10417620 | 58907473 | 29030667 | 35686466 | 360808 | sweet, pungent, herbal | 56 | MS,NIST,RT,STD |
| 1-Octen-3-ol | 401120 | 378333 | 10077140 | 15981346 | 355819 | vegetable, mushroom, chicken | 57 | MS,NIST,RT,STD |
| Maltol | nd | nd | 1413402 | nd | 57812 | sweet, cotton candy, caramellic | 71 | MS,NIST |
| **KETONE** | | | | | | | | |
| 2-Butanone | 71591 | 452816 | 9244 | 29137 | nd | camphoreous, acetone, fruity | 72 | MS,NIST,RT,STD |
| 2-Heptanone | nd | 482128 | 2191455 | 3181706 | 107716 | sweet, spicy, banana | 58 | MS,NIST,RT,STD |
| 6-Methyl-5-hepten-2-one | 4820 | 65250 | 46441 | 47810 | 6977 | musty, banana, fruity | 108 | MS,NIST,RT,STD |
| 3,5-Octadien-2-one | 76714 | 224076 | 778643 | 645502 | nd | fruity, green, grassy | 95 | MS,NIST |
| **AROMATIC COMPOUNDS** | | | | | | | | |
| 2-Ethylfuran | 96714 | 754410 | 441728 | 2047561 | 217345 | malty, cocoa, nutty | 81 | MS,NIST,RT,STD |
| o-Xylene | nd | 214320 | 84182 | 94730 | nd | geranium | 91 | MS,NIST,RT,STD |
| Styrene | nd | 162664 | 192778 | 138832 | nd | sweet, plastic, floral | 104 | MS,NIST,RT,STD |
| Geosmin | nd | nd | nd | nd | nd | musty, earthy, fresh | 112 | MS,NIST,RT,STD |
| Naphthalene | 23092 | 105110 | 62508 | 104647 | 22305 | dry, resinous, pungent | 128 | MS,NIST |
| 2-Pentyl furan | 551366 | 1883562 | 3414641 | 15348415 | 913048 | Fruity, green, earthy beany | 81 | MS,NIST |
| **TERPENOIDS** | | | | | | | | |
| L-limonene | 2182 | 28289 | 4530 | 6323 | nd | camphoreous, herbal, terpenic | 136 | MS,NIST,RT,STD |
| **ALKANES** | | | | | | | | |
| Decane | 5452 | 92780 | 59372 | 134588 | 12885 | unknown | 71 | MS,NIST,RT,STD |
| **SULFUR COMPOUNDS** | | | | | | | | |
| Dimethyl Disulfide | 16977 | 43645 | nd | 69357 | 34845 | vegetable, onion, cabbage | 94 | MS,NIST,RT,STD |
| Methional | nd | nd | nd | nd | 1833 | cabbage, pungent | 48 | MS,NIST,RT,STD |
| Methanethiol | 36153 | 85171 | nd | 55394 | 50009 | vegetable, sulfurous, eggy | 48 | MS,NIST |
| 1-(Methylthio)-propane | nd | nd | nd | nd | nd | garlic, acidic | 61 | MS,NIST |
| **NITROGEN COMPOUNDS** | | | | | | | | |
| 2-butyl-3,5-dimethyl pyrazine | nd | nd | 50956 | 60826 | nd | roasted, nut flavor | 122 | MS,NIST |
| 2,5-Dimethyl pyrazine | nd | nd | nd | nd | nd | nutty, peanut, musty | 108 | MS,NIST,RT,STD |
| **Cranberry cv. ‘CR 2111-1’ (2023)** | | | | | | | | |
|  | **Average Area Counts** | | | | |  |  |  |
| **Compound Name** | **NRF** | **RF** | **NRP** | **RP** | **BP** | **Odor profiles from (The Good Scents Company, 2025)** | **Unique Mass (m/z)** | **Annotation** |
| **ALDEHYDE** | | | | | | | | |
| 2-Methyl butanal | 146368 | 677176 | 90837 | 401006 | 56531 | malty, musty, fermented | 57 | MS,NIST,RT,STD |
| Hexanal | 3815651 | 8743899 | 10630073 | 3868200 | 829517 | vegetable, aldehydic, clean | 57 | MS,NIST,RT,STD |
| (E)-2-hexenal | 28818 | 187542 | 8677973 | 968374 | nd | sweet, vegetable, bitter almond | 55 | MS,NIST,RT,STD |
| Heptanal | 43625 | 398168 | 236081 | 196207 | 63767 | aldehydic, fatty, herbal | 70 | MS,NIST,RT,STD |
| Benzaldehyde | 226753 | 1238214 | 4209334 | 4154298 | 588656 | sweet, cherry, nutty | 77 | MS,NIST,RT,STD |
| Octanal | 34528 | 242935 | 404913 | 341814 | 145878 | aldehydic, fatty, herbal | 44 | MS,NIST,RT,STD |
| Nonanal | 188076 | 1545982 | 1461520 | 1873293 | 564788 | aldehydic, fatty, rose | 57 | MS,NIST,RT,STD |
| Decanal | nd | 268395 | 597140 | 394847 | 122713 | sweet, aldehydic, floral | 41 | MS,NIST,RT,STD |
| **ALCOHOL** | | | | | | | | |
| Butanol | 209800 | 701367 | 119688 | 187992 | nd | sweet, fermented, oily | 31 | MS,NIST,RT,STD |
| 3-Methylbutanol | 206235 | 1129021 | 7327834 | 10994293 | nd | musty, vegetable, cocoa | 42 | MS,NIST,RT,STD |
| 1-Pentanol | 179607 | 607290 | 1244030 | 1928231 | nd | sweet, fermented, yeasty | 31 | MS,NIST,RT,STD |
| 1-Hexanol | 123723 | 913088 | 13367836 | 8160509 | 93406 | sweet, pungent, herbal | 56 | MS,NIST,RT,STD |
| 1-Octen-3-ol | 233650 | 1583822 | 6682980 | 5797704 | 222684 | vegetable, mushroom, chicken | 57 | MS,NIST,RT,STD |
| Maltol | nd | 180655 | nd | 260243 | nd | sweet, cotton candy, caramellic | 71 | MS,NIST |
| **KETONE** | | | | | | | | |
| 2-Butanone | 200524 | 720971 | 47567 | 42443 | 14442 | camphoreous, acetone, fruity | 72 | MS,NIST,RT,STD |
| 2-Heptanone | 36355 | 350369 | 258833 | 174518 | 50638 | sweet, spicy, banana | 58 | MS,NIST,RT,STD |
| 6-Methyl-5-hepten-2-one | 5139 | nd | 86197 | 74472 | 15912 | musty, banana, fruity | 108 | MS,NIST,RT,STD |
| 3,5-Octadien-2-one | 183351 | 1038233 | 513067 | 578645 | nd | fruity, green, grassy | 95 | MS,NIST |
| **AROMATIC COMPOUNDS** | | | | | | | | |
| 2-Ethylfuran | 496530 | 927825 | 1037369 | 514427 | 316492 | malty, cocoa, nutty | 81 | MS,NIST,RT,STD |
| o-Xylene | nd | 190419 | nd | nd | nd | geranium | 91 | MS,NIST,RT,STD |
| Styrene | nd | 113057 | nd | nd | nd | sweet, plastic, floral | 104 | MS,NIST,RT,STD |
| Geosmin | nd | nd | nd | nd | nd | musty, earthy, fresh | 112 | MS,NIST,RT,STD |
| Azulene/Naphthalene | nd | 25779 | 48889 | 57263 | nd | dry, resinous, pungent | 128 | MS,NIST |
| 2-Pentyl furan | 134637 | 920855 | 807391 | 607253 | 446810 | Fruity, green, earthy beany | 81 | MS,NIST |
| **TERPENOIDS** | | | | | | | | |
| L-limonene | 1100 | 11680 | 2676 | 2869 | nd | camphoreous, herbal, terpenic | 136 | MS,NIST,RT,STD |
| **ALKANES** | | | | | | | | |
| Decane | 19309 | 91701 | 14176 | 17767 | 13639 | unknown | 71 | MS,NIST,RT,STD |
| **SULFUR COMPOUNDS** | | | | | | | | |
| Dimethyl Disulfide | 70483 | 112999 | 39092 | 7966765 | 84995 | vegetable, onion, cabbage | 94 | MS,NIST,RT,STD |
| Methional | nd | 13584 | 33444 | 80181 | 4058 | cabbage, pungent | 48 | MS,NIST,RT,STD |
| Methanethiol | nd | 155654 | 28967 | 74849 | 202849 | vegetable, sulfurous, eggy | 48 | MS,NIST |
| 1-(Methylthio)-propane | 73902 | 380781 | 141181 | 1348363 | 93883 | garlic, acidic | 61 | MS,NIST |
| **NITROGEN COMPOUNDS** | | | | | | | | |
| 2-butyl-3,5-dimethyl pyrazine | nd | 58387 | nd | 44759 | nd | roasted, nut flavor | 122 | MS,NIST |
| 2,5-Dimethyl pyrazine | nd | nd | nd | 38798 | nd | nutty, peanut, musty | 108 | MS,NIST,RT,STD |
| **Great Northern cv. ‘Powderhorn’ (2023)** | | | | | | | | |
|  | **Average Area Counts** | | | | |  |  |  |
| **Compound Name** | **NRF** | **RF** | **NRP** | **RP** | **BP** | **Odor profiles from (The Good Scents Company, 2025)** | **Unique Mass (m/z)** | **Annotation** |
| **ALDEHYDE** | | | | | | | | |
| 2-Methyl butanal | 471245 | 856909 | 2698366 | 338557 | nd | malty, musty, fermented | 57 | MS,NIST,RT,STD |
| Hexanal | 14510778 | 12726072 | 28372235 | 2108937 | 773718 | vegetable, aldehydic, clean | 57 | MS,NIST,RT,STD |
| (E)-2-hexenal | 231958 | 223509 | 6106354 | 360380 | nd | sweet, vegetable, bitter almond | 55 | MS,NIST,RT,STD |
| Heptanal | 313024 | 414626 | 537339 | 62300 | 58219 | aldehydic, fatty, herbal | 70 | MS,NIST,RT,STD |
| Benzaldehyde | 1042122 | 1521356 | 5948839 | 4129106 | 304886 | sweet, cherry, nutty | 77 | MS,NIST,RT,STD |
| Octanal | 421006 | 224899 | 385601 | 180647 | 108720 | aldehydic, fatty, herbal | 44 | MS,NIST,RT,STD |
| Nonanal | 2029467 | 1314958 | 1423558 | 690084 | 332458 | aldehydic, fatty, rose | 57 | MS,NIST,RT,STD |
| Decanal | 355471 | 230282 | 327035 | 270104 | 118547 | sweet, aldehydic, floral | 41 | MS,NIST,RT,STD |
| **ALCOHOL** | | | | | | | | |
| Butanol | 681161 | 911548 | 250785 | 164666 | nd | sweet, fermented, oily | 31 | MS,NIST,RT,STD |
| 3-Methylbutanol | 1571530 | 3933239 | 9518067 | 22441144 | 56043 | musty, vegetable, cocoa | 42 | MS,NIST,RT,STD |
| 1-Pentanol | 871435 | 1117227 | 5340355 | 2170323 | 29647 | sweet, fermented, yeasty | 31 | MS,NIST,RT,STD |
| 1-Hexanol | 3578848 | 1374352 | 47364251 | 20366093 | 85631 | sweet, pungent, herbal | 56 | MS,NIST,RT,STD |
| 1-Octen-3-ol | 1761842 | 2265015 | 2644273 | 1530794 | 105891 | vegetable, mushroom, chicken | 57 | MS,NIST,RT,STD |
| Maltol | nd | 215407 | 292340 | 293137 | nd | sweet, cotton candy, caramellic | 71 | MS,NIST |
| **KETONE** | | | | | | | | |
| 2-Butanone | 559994 | 903702 | 58728 | 14548 | nd | camphoreous, acetone, fruity | 72 | MS,NIST,RT,STD |
| 2-Heptanone | 332439 | 678120 | 823503 | 473487 | 28378 | sweet, spicy, banana | 58 | MS,NIST,RT,STD |
| 6-Methyl-5-hepten-2-one | 14615 | 123291 | 36069 | 408 | 4631 | musty, banana, fruity | 108 | MS,NIST,RT,STD |
| 3,5-Octadien-2-one | 1217585 | 1268645 | 966217 | 441728 | 11440 | fruity, green, grassy | 95 | MS,NIST |
| **AROMATIC COMPOUNDS** | | | | | | | | |
| 2-Ethylfuran | 1452153 | 1216692 | 1455114 | 155988 | 355426 | malty, cocoa, nutty | 81 | MS,NIST,RT,STD |
| o-Xylene | 289964 | 403838 | 68835 | nd | 25890 | geranium | 91 | MS,NIST,RT,STD |
| Styrene | 192652 | 358001 | 57732 | 66003 | nd | sweet, plastic, floral | 104 | MS,NIST,RT,STD |
| Geosmin | nd | nd | nd | nd | nd | musty, earthy, fresh | 112 | MS,NIST,RT,STD |
| Azulene/Naphthalene | 62076 | 29586 | 34015 | 51513 | nd | dry, resinous, pungent | 128 | MS,NIST |
| 2-Pentyl furan | 1171249 | 1225280 | 747533 | 365494 | 306606 | Fruity, green, earthy beany | 81 | MS,NIST |
| **TERPENOIDS** | | | | | | | | |
| L-limonene | 14974 | 8939 | 2791 | 1617 | nd | camphoreous, herbal, terpenic | 136 | MS,NIST,RT,STD |
| **ALKANES** | | | | | | | | |
| Decane | 80044 | 104754 | 28907 | 25333 | 19625 | unknown | 71 | MS,NIST,RT,STD |
| **SULFUR COMPOUNDS** | | | | | | | | |
| Dimethyl Disulfide | 152782 | 896070 | 147972 | 621983 | nd | vegetable, onion, cabbage | 94 | MS,NIST,RT,STD |
| Methional | nd | 11326 | 5736 | 135513 | nd | cabbage, pungent | 48 | MS,NIST,RT,STD |
| Methanethiol | 55926 | 448821 | 26567 | 365823 | 64225 | vegetable, sulfurous, eggy | 48 | MS,NIST |
| 1-(Methylthio)-propane | 51908 | 830358 | 70967 | 457229 | nd | garlic, acidic | 61 | MS,NIST |
| **NITROGEN COMPOUNDS** | | | | | | | | |
| 2-butyl-3,5-dimethyl pyrazine | 91380 | 134123 | 1012 | 257586 | nd | roasted, nut flavor | 122 | MS,NIST |
| 2,5-Dimethyl pyrazine | nd | 58342 | 15987 | 33775 | nd | nutty, peanut, musty | 108 | MS,NIST,RT,STD |
| **Manteca cv. ‘Y 1608-14’ (2023)** | | | | | | | | |
|  | **Average Area Counts** | | | | |  |  |  |
| **Compound Name** | **NRF** | **RF** | **NRP** | **RP** | **BP** | **Odor profiles from (The Good Scents Company, 2025)** | **Unique Mass (m/z)** | **Annotation** |
| **ALDEHYDE** | | | | | | | | |
| 2-Methyl butanal | 553900 | 1737419 | 4819722 | 1876589 | 121495 | malty, musty, fermented | 57 | MS,NIST,RT,STD |
| Hexanal | 7503150 | 18663458 | 28234895 | 4380401 | 1132061 | vegetable, aldehydic, clean | 57 | MS,NIST,RT,STD |
| (E)-2-hexenal | 288323 | 446471 | 19847479 | 4583497 | 191242 | sweet, vegetable, bitter almond | 55 | MS,NIST,RT,STD |
| Heptanal | 288429 | 777076 | 921136 | 303494 | 64634 | aldehydic, fatty, herbal | 70 | MS,NIST,RT,STD |
| Benzaldehyde | 647470 | 1820631 | 5319962 | 6326361 | 2126840 | sweet, cherry, nutty | 77 | MS,NIST,RT,STD |
| Octanal | 334780 | 258183 | 648039 | 297964 | 138816 | aldehydic, fatty, herbal | 44 | MS,NIST,RT,STD |
| Nonanal | 2845788 | 1999023 | 2104420 | 1485481 | 807697 | aldehydic, fatty, rose | 57 | MS,NIST,RT,STD |
| Decanal | 572010 | 204935 | 541346 | 241218 | 162140 | sweet, aldehydic, floral | 41 | MS,NIST,RT,STD |
| **ALCOHOL** | | | | | | | | |
| Butanol | 187196 | 1295253 | 115071 | 98798 | nd | sweet, fermented, oily | 31 | MS,NIST,RT,STD |
| 3-Methylbutanol | 179686 | 4092564 | 4205484 | 6752947 | nd | musty, vegetable, cocoa | 42 | MS,NIST,RT,STD |
| 1-Pentanol | 298121 | 1390146 | 3017103 | 1118826 | nd | sweet, fermented, yeasty | 31 | MS,NIST,RT,STD |
| 1-Hexanol | 1967307 | 1287103 | 27001678 | 14117593 | 310979 | sweet, pungent, herbal | 56 | MS,NIST,RT,STD |
| 1-Octen-3-ol | 1009830 | 3053472 | 5592654 | 4577089 | 471570 | vegetable, mushroom, chicken | 57 | MS,NIST,RT,STD |
| Maltol | nd | 43658 | nd | 57693 | nd | sweet, cotton candy, caramellic | 71 | MS,NIST |
| **KETONE** | | | | | | | | |
| 2-Butanone | 452934 | 1343313 | 42377 | 265107 | 12918 | camphoreous, acetone, fruity | 72 | MS,NIST,RT,STD |
| 2-Heptanone | 194910 | 632730 | 374301 | 231815 | 62034 | sweet, spicy, banana | 58 | MS,NIST,RT,STD |
| 6-Methyl-5-hepten-2-one | 64138 | 81141 | 65766 | 56159 | 42012 | musty, banana, fruity | 108 | MS,NIST,RT,STD |
| 3,5-Octadien-2-one | 987203 | 2405194 | 2266421 | 1237785 | 82686 | fruity, green, grassy | 95 | MS,NIST |
| **AROMATIC COMPOUNDS** | | | | | | | | |
| 2-Ethylfuran | 877423 | 4092299 | 3026965 | 815607 | 212755 | malty, cocoa, nutty | 81 | MS,NIST,RT,STD |
| o-Xylene | 190875 | 201995 | nd | nd | 44336 | geranium | 91 | MS,NIST,RT,STD |
| Styrene | 271230 | 226555 | nd | nd | nd | sweet, plastic, floral | 104 | MS,NIST,RT,STD |
| Geosmin | nd | nd | nd | nd | nd | musty, earthy, fresh | 112 | MS,NIST,RT,STD |
| Azulene/Naphthalene | 86537 | 48103 | 59690 | 67008 | 35443 | dry, resinous, pungent | 128 | MS,NIST |
| 2-Pentyl furan | 997336 | 1417659 | 1114061 | 589801 | 226918 | Fruity, green, earthy beany | 81 | MS,NIST |
| **TERPENOIDS** | | | | | | | | |
| L-limonene | 19609 | 10792 | 1369 | 1104 | 2059 | camphoreous, herbal, terpenic | 136 | MS,NIST,RT,STD |
| **ALKANES** | | | | | | | | |
| Decane | 92106 | 69487 | 25654 | 12700 | 12577 | unknown | 71 | MS,NIST,RT,STD |
| **SULFUR COMPOUNDS** | | | | | | | | |
| Dimethyl Disulfide | nd | 965076 | 337103 | 4190278 | 848147 | vegetable, onion, cabbage | 94 | MS,NIST,RT,STD |
| Methional | nd | 22308 | 7924 | 46897 | 33858 | cabbage, pungent | 48 | MS,NIST,RT,STD |
| Methanethiol | 86743 | 486943 | 75523 | 224705 | 299336 | vegetable, sulfurous, eggy | 48 | MS,NIST |
| 1-(Methylthio)-propane | nd | 718180 | 148799 | 435672 | 196952 | garlic, acidic | 61 | MS,NIST |
| **NITROGEN COMPOUNDS** | | | | | | | | |
| 2-butyl-3,5-dimethyl pyrazine | nd | 97217 | nd | 102882 | 29564 | roasted, nut flavor | 122 | MS,NIST |
| 2,5-Dimethyl pyrazine | nd | 64583 | nd | 78010 | nd | nutty, peanut, musty | 108 | MS,NIST,RT,STD |
| **Mayacoba cv. ‘Y 1802-11-2’ (2023)** | | | | | | | | |
|  | **Average Area Counts** | | | | |  |  |  |
| **Compound Name** | **NRF** | **RF** | **NRP** | **RP** | **BP** | **Odor profiles from (The Good Scents Company, 2025)** | **Unique Mass (m/z)** | **Annotation** |
| **ALDEHYDE** | | | | | | | | |
| 2-Methyl butanal | 71951 | 190985 | 13731725 | 812304 | 38214 | malty, musty, fermented | 57 | MS,NIST,RT,STD |
| Hexanal | 1943143 | 2797521 | 8861167 | 986339 | 490394 | vegetable, aldehydic, clean | 57 | MS,NIST,RT,STD |
| (E)-2-hexenal | 40978 | 46257 | 8281540 | 1350182 | 92038 | sweet, vegetable, bitter almond | 55 | MS,NIST,RT,STD |
| Heptanal | 34057 | 87322 | 163594 | 99760 | 34947 | aldehydic, fatty, herbal | 70 | MS,NIST,RT,STD |
| Benzaldehyde | 297265 | 533784 | 2940451 | 3788604 | 1222531 | sweet, cherry, nutty | 77 | MS,NIST,RT,STD |
| Octanal | 58854 | 174986 | 256636 | 295461 | 64573 | aldehydic, fatty, herbal | 44 | MS,NIST,RT,STD |
| Nonanal | 280306 | 796893 | 1044066 | 1614121 | 312219 | aldehydic, fatty, rose | 57 | MS,NIST,RT,STD |
| Decanal | 30215 | 76159 | 272549 | 484456 | 130924 | sweet, aldehydic, floral | 41 | MS,NIST,RT,STD |
| **ALCOHOL** | | | | | | | | |
| Butanol | 107866 | 234054 | 4245 | 285744 | nd | sweet, fermented, oily | 31 | MS,NIST,RT,STD |
| 3-Methylbutanol | 144299 | 577059 | 589618 | 3066170 | nd | musty, vegetable, cocoa | 42 | MS,NIST,RT,STD |
| 1-Pentanol | 75604 | 172991 | 544845 | 506886 | nd | sweet, fermented, yeasty | 31 | MS,NIST,RT,STD |
| 1-Hexanol | 253589 | 160248 | 8953282 | 5345300 | 118152 | sweet, pungent, herbal | 56 | MS,NIST,RT,STD |
| 1-Octen-3-ol | 246890 | 433550 | 5972118 | 4860514 | 391498 | vegetable, mushroom, chicken | 57 | MS,NIST,RT,STD |
| Maltol | nd | 121347 | nd | 111626 | nd | sweet, cotton candy, caramellic | 71 | MS,NIST |
| **KETONE** | | | | | | | | |
| 2-Butanone | 118486 | 339317 | 22383 | 42067 | nd | camphoreous, acetone, fruity | 72 | MS,NIST,RT,STD |
| 2-Heptanone | 40855 | 108415 | 204585 | 174899 | 46937 | sweet, spicy, banana | 58 | MS,NIST,RT,STD |
| 6-Methyl-5-hepten-2-one | 6283 | 192454 | 210247 | 69353 | 14413 | musty, banana, fruity | 108 | MS,NIST,RT,STD |
| 3,5-Octadien-2-one | 224910 | 566611 | 900971 | 592496 | 37268 | fruity, green, grassy | 95 | MS,NIST |
| **AROMATIC COMPOUNDS** | | | | | | | | |
| 2-Ethylfuran | 191283 | 229858 | 800368 | 169400 | 119035 | malty, cocoa, nutty | 81 | MS,NIST,RT,STD |
| o-Xylene | nd | 39758 | 82768 | nd | 42979 | geranium | 91 | MS,NIST,RT,STD |
| Styrene | 29902 | 39056 | 48946 | 50269 | nd | sweet, plastic, floral | 104 | MS,NIST,RT,STD |
| Geosmin | nd | nd | nd | nd | nd | musty, earthy, fresh | 112 | MS,NIST,RT,STD |
| Azulene/Naphthalene | 22208 | 35154 | 71850 | 70244 | 26197 | dry, resinous, pungent | 128 | MS,NIST |
| 2-Pentyl furan | 161523 | 157842 | 346556 | 183045 | 122260 | Fruity, green, earthy beany | 81 | MS,NIST |
| **TERPENOIDS** | | | | | | | | |
| L-limonene | 2031 | 22113 | 6339 | 2268 | nd | camphoreous, herbal, terpenic | 136 | MS,NIST,RT,STD |
| **ALKANES** | | | | | | | | |
| Decane | 9655 | 12408 | 25623 | 20262 | 23470 | unknown | 71 | MS,NIST,RT,STD |
| **SULFUR COMPOUNDS** | | | | | | | | |
| Dimethyl Disulfide | nd | 62766 | nd | 9508711 | 335844 | vegetable, onion, cabbage | 94 | MS,NIST,RT,STD |
| Methional | nd | nd | nd | 45278 | 12295 | cabbage, pungent | 48 | MS,NIST,RT,STD |
| Methanethiol | nd | 149348 | nd | 137521 | 110307 | vegetable, sulfurous, eggy | 48 | MS,NIST |
| 1-(Methylthio)-propane | nd | 279443 | nd | 434377 | nd | garlic, acidic | 61 | MS,NIST |
| **NITROGEN COMPOUNDS** | | | | | | | | |
| 2-butyl-3,5-dimethyl pyrazine | nd | 81361 | nd | 247420 | nd | roasted, nut flavor | 122 | MS,NIST |
| 2,5-Dimethyl pyrazine | nd | 52167 | nd | 111878 | nd | nutty, peanut, musty | 108 | MS,NIST,RT,STD |
| **Navy cv. ‘Alpena’ (2023)** | | | | | | | | |
|  | **Average Area Counts** | | | | |  |  |  |
| **Compound Name** | **NRF** | **RF** | **NRP** | **RP** | **BP** | **Odor profiles from (The Good Scents Company, 2025)** | **Unique Mass (m/z)** | **Annotation** |
| **ALDEHYDE** | | | | | | | | |
| 2-Methyl butanal | 93920 | 268291 | 15849 | 18415 | 203053 | malty, musty, fermented | 57 | MS,NIST,RT,STD |
| Hexanal | 1961877 | 1792488 | 1727300 | 647291 | 1555866 | vegetable, aldehydic, clean | 57 | MS,NIST,RT,STD |
| (E)-2-hexenal | 29476 | 41500 | 1028453 | 316016 | nd | sweet, vegetable, bitter almond | 55 | MS,NIST,RT,STD |
| Heptanal | 59937 | 122543 | 14383 | 11740 | 36564 | aldehydic, fatty, herbal | 70 | MS,NIST,RT,STD |
| Benzaldehyde | 150669 | 412850 | 324497 | 483277 | 518842 | sweet, cherry, nutty | 77 | MS,NIST,RT,STD |
| Octanal | 83045 | 119506 | 37135 | 40181 | 76585 | aldehydic, fatty, herbal | 44 | MS,NIST,RT,STD |
| Nonanal | 581674 | 593941 | 161203 | 89963 | 363440 | aldehydic, fatty, rose | 57 | MS,NIST,RT,STD |
| Decanal | 184470 | 58031 | 28693 | 28199 | 132051 | sweet, aldehydic, floral | 41 | MS,NIST,RT,STD |
| **ALCOHOL** | | | | | | | | |
| Butanol | 158613 | 133903 | nd | 80017 | nd | sweet, fermented, oily | 31 | MS,NIST,RT,STD |
| 3-Methylbutanol | 35157 | 561857 | 552651 | 1245662 | 26768 | musty, vegetable, cocoa | 42 | MS,NIST,RT,STD |
| 1-Pentanol | 74345 | 217622 | 430769 | 287398 | 116362 | sweet, fermented, yeasty | 31 | MS,NIST,RT,STD |
| 1-Hexanol | 182195 | 163773 | 2823254 | 1440020 | 171952 | sweet, pungent, herbal | 56 | MS,NIST,RT,STD |
| 1-Octen-3-ol | 237986 | 520133 | 623070 | 673866 | 180020 | vegetable, mushroom, chicken | 57 | MS,NIST,RT,STD |
| Maltol | nd | 94640 | nd | 28138 | 36052 | sweet, cotton candy, caramellic | 71 | MS,NIST |
| **KETONE** | | | | | | | | |
| 2-Butanone | 70716 | 307757 | 79093 | 19579 | 10019 | camphoreous, acetone, fruity | 72 | MS,NIST,RT,STD |
| 2-Heptanone | 39417 | 104144 | 26180 | 73666 | 44724 | sweet, spicy, banana | 58 | MS,NIST,RT,STD |
| 6-Methyl-5-hepten-2-one | 10122 | 44365 | 5998 | 8090 | 2102 | musty, banana, fruity | 108 | MS,NIST,RT,STD |
| 3,5-Octadien-2-one | 112400 | 218944 | 60748 | 60168 | 36336 | fruity, green, grassy | 95 | MS,NIST |
| **AROMATIC COMPOUNDS** | | | | | | | | |
| 2-Ethylfuran | 84569 | 456487 | 256108 | 298970 | 48150 | malty, cocoa, nutty | 81 | MS,NIST,RT,STD |
| o-Xylene | nd | 40939 | nd | nd | nd | geranium | 91 | MS,NIST,RT,STD |
| Styrene | nd | nd | nd | nd | nd | sweet, plastic, floral | 104 | MS,NIST,RT,STD |
| Geosmin | nd | nd | nd | nd | nd | musty, earthy, fresh | 112 | MS,NIST,RT,STD |
| Azulene/Naphthalene | 19696 | 26053 | nd | 23733 | nd | dry, resinous, pungent | 128 | MS,NIST |
| 2-Pentyl furan | 164968 | 144049 | 134969 | 66664 | 321794 | Fruity, green, earthy beany | 81 | MS,NIST |
| **TERPENOIDS** | | | | | | | | |
| L-limonene | 1201 | 3965 | nd | nd | nd | camphoreous, herbal, terpenic | 136 | MS,NIST,RT,STD |
| **ALKANES** | | | | | | | | |
| Decane | 6171 | 7928 | nd | nd | 12347 | unknown | 71 | MS,NIST,RT,STD |
| **SULFUR COMPOUNDS** | | | | | | | | |
| Dimethyl Disulfide | nd | 57728 | 12053 | 2291747 | 54921 | vegetable, onion, cabbage | 94 | MS,NIST,RT,STD |
| Methional | nd | 2158 | nd | nd | 7006 | cabbage, pungent | 48 | MS,NIST,RT,STD |
| Methanethiol | nd | 161751 | nd | 438757 | 45821 | vegetable, sulfurous, eggy | 48 | MS,NIST |
| 1-(Methylthio)-propane | nd | 282490 | nd | nd | 4815 | garlic, acidic | 61 | MS,NIST |
| **NITROGEN COMPOUNDS** | | | | | | | | |
| 2-butyl-3,5-dimethyl pyrazine | nd | 42106 | nd | nd | nd | roasted, nut flavor | 122 | MS,NIST |
| 2,5-Dimethyl pyrazine | nd | 33783 | nd | nd | nd | nutty, peanut, musty | 108 | MS,NIST,RT,STD |
| **Otebo cv. ‘Samurai’ (2023)** | | | | | | | | |
|  | **Average Area Counts** | | | | |  |  |  |
| **Compound Name** | **NRF** | **RF** | **NRP** | **RP** | **BP** | **Odor profiles from (The Good Scents Company, 2025)** | **Unique Mass (m/z)** | **Annotation** |
| **ALDEHYDE** | | | | | | | | |
| 2-Methyl butanal | 258228 | 533725 | 22000208 | 605448 | 127305 | malty, musty, fermented | 57 | MS,NIST,RT,STD |
| Hexanal | 4167707 | 9645358 | 17800849 | 4877927 | 1217061 | vegetable, aldehydic, clean | 57 | MS,NIST,RT,STD |
| (E)-2-hexenal | 211011 | 172969 | 2939824 | 1098748 | nd | sweet, vegetable, bitter almond | 55 | MS,NIST,RT,STD |
| Heptanal | 225706 | 597428 | 391220 | 134514 | 48633 | aldehydic, fatty, herbal | 70 | MS,NIST,RT,STD |
| Benzaldehyde | 611498 | 1078370 | 3723408 | 4150822 | 263969 | sweet, cherry, nutty | 77 | MS,NIST,RT,STD |
| Octanal | 926685 | 579625 | 395199 | 261727 | 51321 | aldehydic, fatty, herbal | 44 | MS,NIST,RT,STD |
| Nonanal | 6086182 | 3003735 | 1323151 | 1297951 | 185327 | aldehydic, fatty, rose | 57 | MS,NIST,RT,STD |
| Decanal | 475002 | 608148 | 205404 | 209592 | 81785 | sweet, aldehydic, floral | 41 | MS,NIST,RT,STD |
| **ALCOHOL** | | | | | | | | |
| Butanol | 606196 | 491628 | 3474829 | 575816 | 65721 | sweet, fermented, oily | 31 | MS,NIST,RT,STD |
| 3-Methylbutanol | 449193 | 2198475 | 831763 | 880104 | nd | musty, vegetable, cocoa | 42 | MS,NIST,RT,STD |
| 1-Pentanol | 1237593 | 694181 | 2046180 | 961508 | nd | sweet, fermented, yeasty | 31 | MS,NIST,RT,STD |
| 1-Hexanol | 6454520 | 1204137 | 22236466 | 8352552 | 189985 | sweet, pungent, herbal | 56 | MS,NIST,RT,STD |
| 1-Octen-3-ol | 1111295 | 1847874 | 4268621 | 2095396 | 106381 | vegetable, mushroom, chicken | 57 | MS,NIST,RT,STD |
| Maltol | nd | 472015 | nd | nd | nd | sweet, cotton candy, caramellic | 71 | MS,NIST |
| **KETONE** | | | | | | | | |
| 2-Butanone | 229230 | 625323 | 23871 | 54492 | nd | camphoreous, acetone, fruity | 72 | MS,NIST,RT,STD |
| 2-Heptanone | 159773 | 517515 | 340146 | 272313 | 40046 | sweet, spicy, banana | 58 | MS,NIST,RT,STD |
| 6-Methyl-5-hepten-2-one | 46409 | 26210 | 79610 | 43319 | 5738 | musty, banana, fruity | 108 | MS,NIST,RT,STD |
| 3,5-Octadien-2-one | 936322 | 868568 | 923978 | 479289 | nd | fruity, green, grassy | 95 | MS,NIST |
| **AROMATIC COMPOUNDS** | | | | | | | | |
| 2-Ethylfuran | 615429 | 1358395 | 696950 | 452988 | 36166 | malty, cocoa, nutty | 81 | MS,NIST,RT,STD |
| o-Xylene | 247329 | 224926 | 58119 | nd | 153405 | geranium | 91 | MS,NIST,RT,STD |
| Styrene | nd | 200154 | nd | nd | 29801 | sweet, plastic, floral | 104 | MS,NIST,RT,STD |
| Geosmin | nd | nd | nd | nd | nd | musty, earthy, fresh | 112 | MS,NIST,RT,STD |
| Azulene/Naphthalene | 126409 | 37589 | 63314 | 62292 | nd | dry, resinous, pungent | 128 | MS,NIST |
| 2-Pentyl furan | 714501 | 983032 | 476970 | 440364 | 285099 | Fruity, green, earthy beany | 81 | MS,NIST |
| **TERPENOIDS** | | | | | | | | |
| L-limonene | 16114 | 366939 | 2062 | 2562 | nd | camphoreous, herbal, terpenic | 136 | MS,NIST,RT,STD |
| **ALKANES** | | | | | | | | |
| Decane | 28643 | 88106 | 13625 | 22665 | 21270 | unknown | 71 | MS,NIST,RT,STD |
| **SULFUR COMPOUNDS** | | | | | | | | |
| Dimethyl Disulfide | nd | 631237 | 116518 | 7855031 | 91553 | vegetable, onion, cabbage | 94 | MS,NIST,RT,STD |
| Methional | nd | 18115 | 4997 | 53961 | 4105 | cabbage, pungent | 48 | MS,NIST,RT,STD |
| Methanethiol | 1367 | 224360 | nd | 240576 | 111164 | vegetable, sulfurous, eggy | 48 | MS,NIST |
| 1-(Methylthio)-propane | nd | 762184 | nd | 269086 | 65327 | garlic, acidic | 61 | MS,NIST |
| **NITROGEN COMPOUNDS** | | | | | | | | |
| 2-butyl-3,5-dimethyl pyrazine | nd | 124552 | 109596 | 100417 | nd | roasted, nut flavor | 122 | MS,NIST |
| 2,5-Dimethyl pyrazine | nd | 37303 | nd | nd | nd | nutty, peanut, musty | 108 | MS,NIST,RT,STD |
| **White kidney cv. ‘WK 1601-1’ (2023)** | | | | | | | | |
|  | **Average Area Counts** | | | | |  |  |  |
| **Compound Name** | **NRF** | **RF** | **NRP** | **RP** | **BP** | **Odor profiles from (The Good Scents Company, 2025)** | **Unique Mass (m/z)** | **Annotation** |
| **ALDEHYDE** | | | | | | | | |
| 2-Methyl butanal | 980101 | 2830556 | 29762620 | 764076 | 73372 | malty, musty, fermented | 57 | MS,NIST,RT,STD |
| Hexanal | 13507409 | 16622968 | 19030729 | 2363567 | 462365 | vegetable, aldehydic, clean | 57 | MS,NIST,RT,STD |
| (E)-2-hexenal | 214434 | 311744 | 18634031 | 1425902 | nd | sweet, vegetable, bitter almond | 55 | MS,NIST,RT,STD |
| Heptanal | 483883 | 711598 | 415142 | 161234 | 59951 | aldehydic, fatty, herbal | 70 | MS,NIST,RT,STD |
| Benzaldehyde | 792017 | 2287858 | 8809168 | 7037358 | 770733 | sweet, cherry, nutty | 77 | MS,NIST,RT,STD |
| Octanal | 590368 | 935210 | 761974 | 378704 | 192083 | aldehydic, fatty, herbal | 44 | MS,NIST,RT,STD |
| Nonanal | 2016933 | 3351046 | 3273328 | 1856843 | 818245 | aldehydic, fatty, rose | 57 | MS,NIST,RT,STD |
| Decanal | 468517 | 627321 | 691314 | 612755 | 126165 | sweet, aldehydic, floral | 41 | MS,NIST,RT,STD |
| **ALCOHOL** | | | | | | | | |
| Butanol | 726045 | 2310032 | 172424 | 147230 | nd | sweet, fermented, oily | 31 | MS,NIST,RT,STD |
| 3-Methylbutanol | 105150 | 5795043 | 2144094 | 9417954 | nd | musty, vegetable, cocoa | 42 | MS,NIST,RT,STD |
| 1-Pentanol | 367084 | 740840 | 2851557 | 902191 | 57578 | sweet, fermented, yeasty | 31 | MS,NIST,RT,STD |
| 1-Hexanol | 999790 | 1135970 | 30693176 | 8176701 | 89651 | sweet, pungent, herbal | 56 | MS,NIST,RT,STD |
| 1-Octen-3-ol | 1706338 | 2892669 | 8618808 | 8901172 | 290539 | vegetable, mushroom, chicken | 57 | MS,NIST,RT,STD |
| Maltol | nd | 557840 | nd | 269580 | nd | sweet, cotton candy, caramellic | 71 | MS,NIST |
| **KETONE** | | | | | | | | |
| 2-Butanone | 783225 | 1878721 | 53263 | 26163 | nd | camphoreous, acetone, fruity | 72 | MS,NIST,RT,STD |
| 2-Heptanone | 263549 | 880920 | 600186 | 207648 | 45385 | sweet, spicy, banana | 58 | MS,NIST,RT,STD |
| 6-Methyl-5-hepten-2-one | 47903 | 272487 | 4 | 96223 | 11967 | musty, banana, fruity | 108 | MS,NIST,RT,STD |
| 3,5-Octadien-2-one | 910734 | 1576192 | 2118573 | 800942 | nd | fruity, green, grassy | 95 | MS,NIST |
| **AROMATIC COMPOUNDS** | | | | | | | | |
| 2-Ethylfuran | 1353038 | 4161124 | 1047131 | 293805 | 434440 | malty, cocoa, nutty | 81 | MS,NIST,RT,STD |
| o-Xylene | 157287 | 232588 | nd | nd | 71014 | geranium | 91 | MS,NIST,RT,STD |
| Styrene | 209683 | 330352 | nd | nd | nd | sweet, plastic, floral | 104 | MS,NIST,RT,STD |
| Geosmin | nd | nd | nd | nd | nd | musty, earthy, fresh | 112 | MS,NIST,RT,STD |
| Azulene/Naphthalene | 47110 | 103987 | 77458 | 93382 | nd | dry, resinous, pungent | 128 | MS,NIST |
| 2-Pentyl furan | 928363 | 971437 | 677011 | 444757 | 554267 | Fruity, green, earthy beany | 81 | MS,NIST |
| **TERPENOIDS** | | | | | | | | |
| L-limonene | 10776 | 164140 | 2247 | 3598 | nd | camphoreous, herbal, terpenic | 136 | MS,NIST,RT,STD |
| **ALKANES** | | | | | | | | |
| Decane | 70840 | 91904 | 65388 | 20317 | 8766 | unknown | 71 | MS,NIST,RT,STD |
| **SULFUR COMPOUNDS** | | | | | | | | |
| Dimethyl Disulfide | nd | 837963 | 90758 | 2559976 | 73026 | vegetable, onion, cabbage | 94 | MS,NIST,RT,STD |
| Methional | nd | 37128 | nd | 66396 | 10267 | cabbage, pungent | 48 | MS,NIST,RT,STD |
| Methanethiol | 84519 | 602517 | 40087 | 407053 | 232405 | vegetable, sulfurous, eggy | 48 | MS,NIST |
| 1-(Methylthio)-propane | nd | 2019627 | 182613 | 733109 | 65359 | garlic, acidic | 61 | MS,NIST |
| **NITROGEN COMPOUNDS** | | | | | | | | |
| 2-Butyl-3,5-dimethyl pyrazine | nd | 438465 | nd | 189835 | nd | roasted, nut flavor | 122 | MS,NIST |
| 2,5-Dimethyl pyrazine | nd | 272947 | nd | 271301 | nd | nutty, peanut, musty | 108 | MS,NIST,RT,STD |

Table S2 (cont’d)
